# Novel Hit Compounds Against a Neglected Sexually Transmitted Infection: Synthesis and Trichomonacidal Activity of 1,3-Thiazolidin-4-One Derivatives

**DOI:** 10.3390/pharmaceutics18010110

**Published:** 2026-01-15

**Authors:** Alexia Brauner de Mello, Melinda G. Victor, Wilson Cunico, Jorge Fernández-Villalba, Frederico Schmitt Kremer, Lucas Mocellin Goulart, Juan José García-Rodríguez, Camila Belmonte Oliveira, Alexandra Ibáñez-Escribano

**Affiliations:** 1 Parasitology Laboratory, Department of Microbiology and Parasitology, Federal University of Pelotas, Campus S/N, Capão do Leão 96160-000, RS, Brazil; alexiabraunermello@gmail.com; 2 Laboratory of Chemistry Applied to Bioactives, Center for Chemical, Pharmaceutical and Food Sciences, Federal University of Pelotas, Campus S/N, Capão do Leão 96160-000, RS, Brazil; melindagv@gmail.com (M.G.V.); wjcunico@yahoo.com.br (W.C.); 3 Department of Microbiology and Parasitology, Faculty of Pharmacy, Complutense University of Madrid, 28040 Madrid, Spain; jorgef15@ucm.es (J.F.-V.); alexandraibanez@ucm.es (A.I.-E.); 4 Laboratory of Bioinformatics-Omixlab, Technological Development Center, Federal University of Pelotas, Campus S/N, Capão do Leão 96160-000, RS, Brazil; fred.s.kremer@gmail.com (F.S.K.); lmocellingoulart@gmail.com (L.M.G.)

**Keywords:** trichomoniasis, thiazolidines, antiprotozoal agents, *Trichomonas vaginalis*, molecular docking

## Abstract

**Background**: Infections caused by the protozoan *Trichomonas vaginalis* affect millions of people worldwide and are responsible for one of the most common sexually transmitted diseases. Despite the efficacy of 5-nitroimidazoles like metronidazole, concerns regarding widespread resistance and the absence of viable alternatives for specific patient populations necessitate the development of structurally diverse pharmacological agents. In this study, we investigated the antiparasitic activity of 1,3-thiazolidin-4-one derivatives against *T. vaginalis*. **Methods**: Thiazolidines were synthesized via multicomponent reaction (MCR) using one-pot methodology and tested in vitro against the parasite and mammalian cell lines. **Results**: Seventy percent of the compounds showed more than 80% antiparasitic activity at 100 μM, with compounds **4a**, **4b**, and **4f** exhibiting IC_50_ ≤ 20 µM. None of the molecules exhibited cytotoxic against Vero CCL-81 and HeLa cells. Evaluation of the structure–activity relationship (SAR) indicates that the substituent at the nitrogen position of the heterocycle may be involved in the antiparasitic effect of these compounds. In silico studies also revealed that the three compounds possess adequate oral bioavailability and do not present mutagenic, tumorigenic or irritating risks. Finally, molecular docking predicted strong interactions of compounds **4a**, **4b**, and **4f** with *T. vaginalis* enzymes lactate dehydrogenase and purine nucleoside phosphorylase; compound **4f** also interacted with methionine Ƴ-lyase. **Conclusions**: These preliminary results suggest that 1,3-thiazolidin-4-ones are promising scaffolds for developing new trichomonacidal agents.

## 1. Introduction

Trichomoniasis is associated with a broad spectrum of clinical manifestations, ranging from asymptomatic cases to severe presentations characterized by inflammatory processes and clinical signs [1] that could be mistaken for other genitourinary conditions. In women, this sexually transmitted infection (STI) is frequently associated with adverse outcomes including preterm births, low-birth-weight newborns, infertility, and cervical cancer [2,3]. In men, the disease is mainly asymptomatic [4], although can be manifested as nongonococcal urethritis and infertility [5]. Furthermore, *T. vaginalis* infection augments other STIs including HIV acquisition [6].

Despite its widespread use, metronidazole (MTZ) presents relevant limitations, including adverse gastrointestinal and neurological effects, contraindications during certain stages of pregnancy, drug–drug interactions, and intolerance in a subset of patients, which may compromise treatment adherence and clinical outcomes [1]. These limitations highlight the importance of exploring new trichomonacidal compounds with distinct structural features and adequate safety and tolerability profiles, even when evaluated using MTZ-sensitive isolates [7].

Heterocyclic compounds have gained considerable attention due to their wide range of medical and biological applications, with more than 90% of new drugs containing these structures [8]. Several studies have demonstrated their diverse therapeutic potential, including antifungal, antibacterial, antiviral, anthelmintic, antioxidant, anti-inflammatory, anticonvulsant, antipyretic, antiallergic, anticancer or antihypertensive potential, among others [9].

1,3-Thiazolidin-4-ones have gained significant attention in medicinal chemistry due to their broad pharmacological properties, particularly as potential antiparasitic agents [10]. This scaffold features a thiazolidine ring that provides structural flexibility, facilitating the optimization of biological activity [11]. Furthermore, the availability of multiple substitution sites offers opportunities to enhance selectivity and reduce toxicity, making these scaffolds promising candidates for the development of new antiparasitic agents [12,13]. Furthermore, their relative ease of synthesis and cost-effectiveness further support their continued investigation in preclinical and clinical research.

Several FDA-approved medicines include sulfur heterocycles, like clopidogrel and raloxifene, among others, highlighting the clinical relevance of this class of compounds across a wide diverse range of medical disorders [8]. Numerous bioactive molecules bearing thiazole- or thiazolidine-related motifs have been explored for diverse biological activities, including thiazolidine-2,4-diones such as pioglitazone and rosiglitazone for the treatment of diabetes [8,10], as well as CP-060S, which exhibits calcium overload inhibition and antioxidant activity [14]. Although CP-060S ultimately failed in later-stage clinical trials, its structure contains a bulky phenolic substitution closely resembling sterically hindered phenols such as di(*tert*-butyl)phenol (BHT), a feature that contributed significantly to its antioxidant properties [14,15].

In parallel, the BHT fragment has been explored for its role in oxidative stress modulation. Beyond its antioxidant properties, BHT is a lipophilic, redox-stable, and membrane-interacting motif that has demonstrated additional biological activities, including dual 5-lipoxygenase and cyclooxygenase inhibition [15]. These characteristics make BHT particularly attractive for the design of hybrid scaffolds that combine distinct pharmacophores [16].

**Figure 1:**
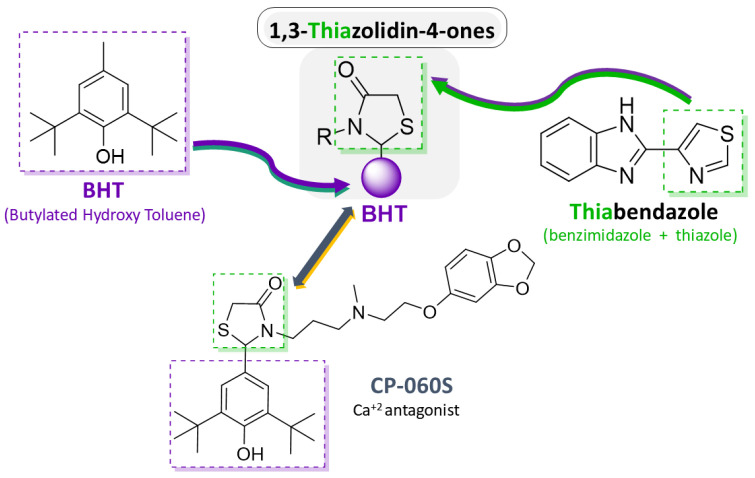
Structural comparison of novel hybrid 1,3-thiazolidin-4-one compounds, rationally designed based on thiabendazole and CP-060S.

In this context, the development of hybrid molecules incorporating BHT-like moieties and 1,3-thiazolidin-4-one cores is supported by the independent pharmacological precedents of each motif in antiparasitic and antioxidant research. The incorporation of the BHT fragment is complemented by the role of the thiazolidin-4-one ring as a privileged antiprotozoal scaffold [17] and as a conformationally restricted bioisosteric analog of thiazole-containing pharmacophores [10]. Notably, the thiazolidin-4-one ring serves as a partially saturated analog of thiazole systems present in clinical antiparasitic agents like thiabendazole [8,9]. While thiabendazole is a benzimidazole derivative known to inhibit microtubule assembly by affecting the colchicine binding-site of β-tubulin monomers, it has specifically demonstrated the ability to arrest parasite cytokinesis in *Tritrichomonas foetus* [18]. This structural analogy (Figure 1) further supports the continued exploration of thiazolidin-4-one derivatives as relevant frameworks for antiparasitic drug discovery.

Therefore, this study describes the synthesis and comprehensive in vitro evaluation of seven novel thiazolidin-4-one derivatives against *T. vaginalis* parasites. The biological assessment included evaluation of nonspecific cytotoxicity profile using VERO CCL-81 and HeLa cell lines. In addition, in silico studies were conducted to estimate oral bioavailability, predicted toxicity, and potential interactions with parasitic proteins through molecular docking.

## 2. Materials and Methods

### 2.1. Chemistry

#### 2.1.1. General Methodology

All solvents and reagents were purchased from commercial suppliers at high purity (>95%) and used without further purification, except for toluene, which was distilled and dried prior to use. Thin-layer chromatography (TLC) analyses were performed on silica gel 60 aluminum plates with fluorescent indicator (Merck, Darmstadt, Germany), and flash column chromatography was conducted on silica gel 60 (Merck, particle size 0.040–0.063 mm). Melting points of solid compounds were determined using a Fisatom apparatus (model 430; Fisatom Equipamentos Científicos Ltda, São Paulo, SP, Brazil) equipped with three capillary tubes and a thermometer with a maximum range of 360 °C. Low-resolution mass spectra (MS) and chromatograms were obtained using a GCMS-QP2010 Plus instrument (Shimadzu Scientific Instruments, Columbia, MD, USA) equipped with a Na-1MS capillary column (30 m × 0.25 mm × 0.25 µm). Electron ionization (EI) was used as the ionization mode. The GC oven program began at 50 °C (held for 2 min), followed by heating at 20 °C/min to 280 °C, where the temperature was maintained for 20 min. Samples were introduced through a split/splitless injector (mode selected according to analyte concentration). Injector and detector temperatures were set at 300 °C and 200 °C, respectively. Helium was used as the carrier gas at a constant flow rate of 0.99 mL/min. Retention times (RTs) were measured according to the specified conditions. Nuclear Magnetic Resonance (NMR) spectra were recorded using a Bruker Avance III HD spectrometer (Bruker Corporation; Billerica, MA, USA) operating at 400 MHz for ^1^H and 101 MHz for ^13^C. CDCl_3_ containing tetramethylsilane (TMS) served as the internal standard. Chemical shifts (δ, ppm) are reported relative to TMS, and coupling constants (J, Hz) are given for ^1^H-^1^H couplings. High-resolution mass spectrometry (HRMS) analyses were performed on a Thermo Scientific Q Exactive Focus Quadrupole-Orbitrap mass spectrometer (Thermo Fisher Scientific; Waltham, MA, USA) equipped with a HESI (Heated Electrospray Ionization) source. Data acquisition and processing were carried out using TraceFinder software 4.1 (Thermo Fisher Scientific; Waltham, MA, USA). Full MS scans were recorded in positive ionization mode at a resolution of 70,000 full width at half maximum (FWHM) at m/z 200). The HESI source parameters were as follows: sheath gas flow rate = 25 (arbitrary units), auxiliary gas = 10 (arbitrary units), spray voltage = 3.0 kV, and capillary temperature = 330 °C. All spectral data were processed using MestReNova 12.0.1-20560 (Mestrelab Research S.L., Santiago de Compostela, Spain). Chemical structures and associated physicochemical data were drawn and/or generated using ChemDraw Professional 22.2.0 (PerkinElmer Informatics).

#### 2.1.2. Synthesis of 2-(3,5-di-*tert*-butyl-4-hydroxyphenyl)-3-substituted-thiazolidin-4-ones (**4**)

**Scheme 1:**
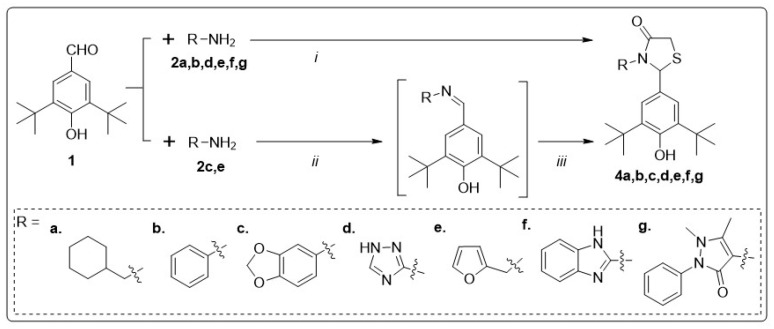
Synthesis of 1,3-thiazolidin-4-ones (**4**) via one-pot multicomponent reactions. Conditions: (i) HSCH_2_COOH (**3**), dry toluene, reflux, 24 h, 29–92%; (ii) dry toluene, 80–110 °C, 3 h; (iii) HSCH_2_COOH (**3**), dry toluene, reflux, 21 h, 37–80%. Yields refer to isolated and purified products.

The target 2-(3,5-di-*tert*-butyl-4-hydroxyphenyl)-3-substituted-thiazolidin-4-ones (**4**) were synthesized via one-pot multicomponent reactions under conventional thermal heating in a silicone oil bath, as shown in Scheme 1. As all procedures employ a single vessel in which two or more reactants are combined either concurrently or sequentially to afford the final product, they are classified as multicomponent one-pot reactions (MCRs). After completion of reactions, extraction, and purification, final products were obtained in moderate to high yields (29–92%).

Characterization data for all compounds are presented below, and complete analyses are provided in the Supplementary Material.

3-(Cyclohexylmethyl)-2-(3,5-di-*tert*-butyl-4-hydroxyphenyl)thiazolidin-4-one **4a**

C_24_H_37_NO_2_S, M.W.: 403.63 g/mol, CLogP: 6.98, light orange solid, yield: 0.1993 g (49%), m.p.: 132–134 °C.
TLC System: 7:3 Hexane:Ethyl Acetate. Rf: 0.81
^1^H NMR (400 MHz, CDCl_3_): δ (ppm, *J*_H-H_ = Hz): 7.07 (s, 2H, H2.2, H2.6), 5.56 (s, 1H, H2), 5.33 (s, 1H, H2.7), 3.79 (dd, ^2^*J* = 15.48, ^4^*J* = 1.93, 1H, H5a), 3.69 (d, ^2^*J* = 15.46, 1H, H5b), 3.42 (dd, ^2^*J* = 13.72, ^3^*J* = 8.32, 1H, H3.1a), 2.55 (dd, ^2^*J* = 13.72, ^3^*J* = 6.52, 1H, H3.1b), 1.73–1.52 (m, 6H, cyclohexyl), 1.43 (s, 18H, H2.10–H2.15), 1.18–1.07 (m, 3H, cyclohexyl), 0.97–0.81 (m, 2H, cyclohexyl).
^13^C NMR (101 MHz, CDCl_3_) δ (ppm): 171.5 (C4), 154.5 (C2.4), 136.6 (2C, C2.3, C2.5), 129.7 (C2.1), 124.0 (2C, C2.2, C2.6), 65.1 (C2), 49.2 (C3.1), 35.8 (1C, cyclohexyl), 34.5 (2C, C2.8, C2.9), 33.1 (C5), 31.1 (1C, cyclohexyl), 30.6 (1C, cyclohexyl), 30.4 (6C, C2.10-C2.15), 26.4 (1C, cyclohexyl), 26.0 (1C, cyclohexyl), 25.8 (1C, cyclohexyl).
GC: RT = 14.7 min.
MS (70 eV): m/z (%) = 403 (M^+^, 15), 370 (36), 328 (17), 307 (9), 260 (52), 250 (13), 234 (9), 219 (9), 110 (16), 102 (10), 67 (10), 57 (100), 55 (66), 43 (11), 41 (50).
HRMS (ESI) m/z: [M + H]^+^ calculated exact mass (Trace Finder) for C24H37NO2S = 404.2617, found = 404.2615.

2-(3,5-Di-*tert*-butyl-4-hydroxyphenyl)-3-phenylthiazolidin-4-one **4b**

C_23_H_29_NO_2_S, M.W.: 383.55 g/mol, CLogP: 6.37, light orange solid, yield: 0.2177 g (57%), m.p.: 141–143 °C.
TLC System: 7:3 Hexane:Ethyl Acetate. Rf: 0.70
^1^H NMR (400 MHz, CDCl_3_): δ (ppm, *J*_H-H_ = Hz): 7.29–7.24 (m, 2H, aryl), 7.18–7.13 (m, 1H, H3.4), 7.11–7.06 (m, 2H, aryl), 7.04 (s, 1H, H2.2, H2.6), 6.05 (s, 1H, H2), 5.21 (s, 1H, H2.7), 3.95 (dd, ^2^*J* = 15.77, ^4^*J* = 1.58 Hz, 1H, H5a), 3.86 (dd, ^2^*J* = 15.77, ^4^*J* = 0.86 Hz, 1H, H5b), 1.35 (s, 18H, H2.10–H2.15).
^13^C NMR (101 MHz, CDCl_3_) δ (ppm): 171.2 (C4), 154.3 (C2.4), 137.8 (C3.1), 136.2 (2C, C2.3, C2.5), 129.2 (C2.1), 129.1 (2C, aryl), 127.2 (C3.4), 126.4 (2C, aryl), 124.1 (2C, C2.2, C2.6), 66.6 (C2), 34.4 (2C, C2.8, C2.9), 33.8 (C5), 30.3 (6C, C2.10–C2.15).
GC: RT = 14.3 min.
MS (70 eV): m/z (%) = 383 (M^+^, 17), 261 (10), 260 (51), 249 (9), 207 (27), 104 (41), 77 (36), 57 (100), 41 (23).
HRMS (ESI) m/z: [M + H]^+^ calculated exact mass (Trace Finder) for C23H29NO2S = 384.1991, found = 384.1990.

3-(Benzo[*d*][1,3]dioxol-5-yl)-2-(3,5-di-*tert*-butyl-4-hydroxyphenyl)thiazolidin-4-one **4c**

C_24_H_29_NO_4_S, M.W.: 427.56 g/mol, CLogP: 6.33, dark red solid, yield: 0.3432 g (80%), m.p.: 135–137 °C.
TLC System: 7:3 Hexane:Ethyl Acetate. Rf: 0.59
^1^H NMR (400 MHz, CDCl_3_): δ (ppm, *J*_H-H_ = Hz): 7.04 (s, 2H, H2.2, H2.6), 6.68 (d, ^3^*J* = 8.14, 1H, aryl), 6.53 (d, ^4^*J* = 2.04, 1H, aryl), 6.50 (dd, ^3^*J* = 8.19, ^4^*J* = 2.07, 1H, aryl), 5.91–5.89 (m, 3H, H2, H3.2), 5.25 (s, 1H, H2.7), 3.93 (dd, ^2^*J* = 15.77, ^4^*J* = 1.69, 1H, H5a), 3.84 (d, ^2^*J* = 15.77, 1H, H5b), 1.37 (s, 18H, H2.10–H2.15).
^13^C NMR (101 MHz, CDCl_3_) δ (ppm): 171.3 (C4), 154.4 (C2.4), 148.0 (1C, aryl), 146.8 (1C, aryl), 136.3 (2C, C2.3, C2.5), 131.5 (1C, aryl), 129.1 (C2.1), 124.3 (2C, C2.2, C2.6), 120.4 (1C, aryl), 108.3 (1C, aryl), 108.2 (1C, aryl), 101.6 (C3.2), 66.9 (C2), 34.4 (2C, C2.8, C2.9), 33.6 (C5), 30.3 (6C, C2.10–C2.15).
GC: RT = 17.5 min.
MS (70 eV): m/z (%) = 427 (M^+^, 26), 353 (4), 261 (18), 260 (100), 207 (10), 148 (8), 57 (31), 41 (5).
HRMS (ESI) m/z: [M + H]^+^ calculated exact mass (Trace Finder) for C24H29NO4S = 428.1890, found = 428.1890.

2-(3,5-di-*tert*-butyl-4-hydroxyphenyl)-3-(*1H*-1,2,4-triazol-3-yl)thiazolidin-4-one **4d**

C_19_H_26_N_4_O_2_S, M.W.: 374.50 g/mol, CLogP: 3.94, white solid, yield: 0.1391 g (37%), m.p.: 229–231 °C.
TLC System: 7:3 Hexane:Ethyl Acetate. Rf: 0.20
^1^H NMR (400 MHz, CDCl_3_): δ (ppm, *J*_H-H_ = Hz): 7.77 (s, 1H, H3.3), 7.13 (s, 2H, H2.2, H2.6), 6.48 (s, 1H, H2), 5.27 (s, 1H, H2.7), 4.05 (d, ^2^*J* = 16.59, 1H, H5a), 3.77 (d, ^2^*J* = 16.54, 1H, H5b), 1.39 (s, 18H, H2.10–H2.15).
^13^C NMR (101 MHz, CDCl_3_) δ (ppm): 172.4 (C4), 154.3 (C2.4), 149.2 (1C, triazolyl), 148.8 (1C, triazolyl), 136.4 (2C, C2.3, C2.5), 130.0 (C2.1), 122.8 (2C, C2.2, C2.6), 63.0 (C2), 34.5 (2C, C2.8, C2.9), 33.0 (C5), 30.3 (6C, C2.10–C2.15).
GC: RT = 14.8 min.
MS (70 eV): m/z (%) = 375 (M^+1^, 14), 374 (M^+^, 64), 332 (59), 306 (22), 249 (44), 207 (16), 169 (100), 141 (16), 115 (66), 95 (27), 57 (79), 41 (28).
HRMS (ESI) m/z: [M + H]^+^ calculated exact mass (Trace Finder) for C19H26N4O2S = 375.1849, found = 375.1847.

2-(3,5-di-*tert*-butyl-4-hydroxyphenyl)-3-(furan-2-ylmethyl)thiazolidin-4-one **4e**

C_22_H_29_NO_3_S, M.W.: 387.54 g/mol, CLogP: 5.06, light orange solid, yield: 0.1421 g (37%), m.p.: 112–114 °C.
TLC System: 7:3 Hexane:Ethyl Acetate. Rf: 0.85
^1^H NMR (400 MHz, CDCl_3_): δ (ppm, *J*_H-H_ = Hz): 7.34 (s, 1H, furyl), 7.10 (s, 2H, H2.2, H2.6), 6.28 (dd, ^4^*J* = 3.21, ^4^*J* = 1.86 Hz, 1H, furyl), 6.06 (d, ^4^*J* = 3.17 Hz, 1H), 5.49 (s, 1H, H2), 5.35 (s, 1H, H2.7), 4.89 (d, ^2^*J* = 15.35 Hz, 1H, H3.1a), 3.82 (dd, ^2^*J* = 15.63, ^4^*J* = 1.97 Hz, 1H, H5a), 3.72 (d, ^2^*J* = 15.67 Hz, 1H, H5b), 3.71 (d, ^2^*J* = 15.27 Hz, 1H, H3.1b), 1.43 (s, 18H, H2.10–H2.15).
^13^C NMR (101 MHz, CDCl_3_) δ (ppm): 171.1 (C4), 154.7 (C2.4), 149.5 (1C, furyl), 142.7 (1C, furyl), 136.5 (2C, C2.3, C2.5), 128.5 (C2.1), 124.7 (2C, C2.2, C2.6), 110.4 (1C, furyl), 109.1 (1C, furyl), 64.2 (C2), 39.1 (C3.1), 34.5 (2C, C2.8, C2.9), 33.3 (C5), 30.3 (6C, C2.10–C2.15).
GC: RT = 13.8 min.
MS (70 eV): m/z (%) = 388 (M^+1^, 19), 387 (M^+^, 68), 313 (22), 312 (75), 250 (10), 251 (45), 235 (14), 138 (12), 137 (77), 109 (14), 96 (11), 81 (100), 57 (34), 41 (14).
HRMS (ESI) m/z: [M + H]^+^ calculated exact mass (Trace Finder) for C22H29NO3S = 388.1940, found = 388.1939.

3-(*1H*-benzo[d]imidazol-2-yl)-2-(3,5-di-*tert*-butyl-4-hydroxyphenyl)thiazolidin-4-one **4f**

C_24_H_29_N_3_O_2_S, M.W.: 423.58 g/mol, CLogP: 5.79, light yellow flakes, yield: 0.1245 g (29%), m.p.: 216–218 °C.
TLC System: 7:3 Hexane:Ethyl Acetate. Rf: 0.72
^1^H NMR (400 MHz, CDCl_3_): δ (ppm, *J*_H-H_ = Hz): 11.04 (s, 1H, H3.1, NH), 7.62–7.55 (m, 1H, H3.5), 7.43–7.36 (m, 1H, H3.6), 7.26 (s, 2H, H2.2, H2.6), 7.25–7.17 (m, 1H, H3.7), 7.20–7.18 (m, 1H, H3.4), 6.76 (s, 1H, H2), 5.23 (s, 1H, H2.7), 4.12 (d, ^2^*J* = 16.45, 1H, H5a), 3.80 (d, ^2^*J* = 16.49, 1H, H5b), 1.38 (s, 18H, H2.10–H2.15).
^13^C NMR (101 MHz, CDCl_3_) δ (ppm): 172.8 (C4), 154.2 (C2.4), 145.3 (C3.2), 140.4 (C3.7a), 136.1 (2C, C2.3, C2.5), 131.6 (C3.3a), 130.6 (C2.1), 123.2 (2C, C2.2, C2.6), 122.6 (C3.7), 122.5 (C3.4), 118.8 (C3.5), 110.8 (C3.6), 63.2 (C2), 34.5 (2C, C2.8, C2.9), 33.7 (C5), 30.3 (6C, C2.10–C2.15).
GC: RT = 17.0 min.
MS (70 eV): m/z (%) = 424 (M^+1^, 25), 423 (M^+^, 91), 382 (20), 381 (74), 377 (18), 348 (12), 249 (38), 218 (14), 207 (26), 176 (34), 164 (100), 160 (36), 146 (22), 133 (16), 119 (13), 57 (53), 41 (15).
HRMS (ESI) m/z: [M + H]^+^ calculated exact mass (Trace Finder) for C24H29N3O2S = 424.2053, found = 424.2052.

2-(3,5-di-*tert*-butyl-4-hydroxyphenyl)-3-(1,5-dimethyl-3-oxo-2-phenyl-2,3-dihydro-*1H*-pyrazol-4-yl)thiazolidin-4-one **4g**

C_28_H_35_N_3_O_3_S, M.W.: 493.67 g/mol, CLogP: 4.03, light orange solid, yield: 0.4588 g (92%), m.p.: 223–225 °C.
TLC System: 7:3 Hexane:Ethyl Acetate. Rf: 0.0625
^1^H NMR (400 MHz, CDCl_3_): δ (ppm, *J*_H-H_ = Hz): 7.36 (t, ^3^*J* = 7.80 Hz, 2H, H3.2.3, H3.2.5), 7.24–7.17 (m, 3H, H3.2.2, H3.2.4, H.3.2.6), 7.15 (s, 2H, H2.2, H2.6), 6.22 (s, 1H, H2), 5.21 (s, 1H, H2.7), 3.84 (d, ^2^*J* = 15.71, 1H, H5b), 3.76 (dd, ^2^*J* = 15.77, ^4^*J* = 1.59 Hz, 1H, H5a), 2.87 (s, 3H, H3.1.1), 1.77 (s, 3H, H3.5.1), 1.30 (s, 18H, H2.10–215).
^13^C NMR (101 MHz, CDCl_3_) δ (ppm): 170.9 (C4), 161.4 (C3.3), 154.5 (C2.4), 153.6 (C3.5), 136.0 (2C, C2.3, C2.5), 134.7 (1C, aryl), 129.3 (2C, aryl), 128.9 (C2.1), 127.1 (1C, aryl), 125.4 (2C, aryl), 124.3 (2C, C2.2, C2.6), 107.7 (C3.4), 63.6 (C2), 35.9 (C3.1.1), 34.5 (2C, C2.8, C2.9), 33.4 (C5), 30.4 (6C, C2.10–C2.15), 11.1 (C3.5.1).
GC: RT = 28, 5 min.
MS (70 eV): m/z (%) = 494 (M^+1^, 10), 493 (M^+^, 29), 419 (11), 418 (23), 327 (33), 248 (10), 246 (10), 233 (37), 207 (23), 189 (18), 188 (56), 96 (14), 77(12), 57 (42), 56 (100).
HRMS (ESI) m/z: [M + H]^+^ calculated exact mass (Trace Finder) for C28H35N3O3S = 494.2471, found = 494.2470.

#### 2.1.3. Synthesis Route A

Compounds **4a**, **4b**, **4d**, **4f**, and **4g** were synthesized via one-pot multicomponent reactions by combining 3,5-di-*tert*-butyl-4-hydroxybenzaldehyde (**1**, 1 mmol), a substituted primary amine (**2**, 1 mmol), and mercaptoacetic acid (**3**, 2 mmol) in dry toluene (35–40 mL) in a 50 mL round-bottom flask. Mercaptoacetic acid was added at the beginning of all reactions except for those leading to compounds **4c** and **4e** (synthesis route B). A Dean–Stark apparatus was used for azeotropic removal of water. Reaction progress was monitored by TLC on silica gel using hexane:ethyl acetate (7:3) as the eluent.

#### 2.1.4. Synthesis Route B

The synthesis of compound **4e** required a modified procedure due to the low boiling point of its corresponding amine. An initial lower-temperature step facilitated formation of the imine intermediate before addition of mercaptoacetic acid. In contrast, compound **4c** was prepared under reflux following the conditions previously reported by Masteloto et al. (2015) [19]. 3,5-di-*tert*-butyl-4-hydroxybenzaldehyde (**1**, 1 mmol) and either furfurylamine (**2e**, 1 mmol) or 3,4-(methylenedioxy)aniline (**2c**, 1 mmol) were combined in dry toluene (35–40 mL) in a 50 mL round-bottom flask. For the stepwise one-pot process, mercaptoacetic acid (3) was added after 3 h of heating with stirring (80 °C for **4e**; 110 °C for **4c**). The reaction mixture was then heated for an additional 21 h at reflux for both compounds (increase in temperature for **4e**). Total reaction time was 24 h, with a Dean–Stark apparatus employed for water removal.

Following reaction completion in routes, the solvent was evaporated under reduced pressure and the crude product was washed with saturated aqueous NaHCO_3_. If a precipitate formed, it was isolated by vacuum filtration; otherwise, liquid–liquid extraction was performed using a separatory funnel and an organic solvent (ethyl acetate or dichloromethane). The organic layer was dried over anhydrous MgSO_4_, filtered, and concentrated under reduced pressure to give the crude product.

Purification was achieved by recrystallization from hexane:ethyl acetate (9:1), removal of impurities/starting reactants via trituration with hot hexane or hexane:ethyl acetate (9:1), or flash column chromatography on silica gel with hexane:ethyl acetate gradients (9:1 to 1:1). Pure compounds (≥98%) were characterized by MS and NMR (^1^H and ^13^C). HRMS analyses confirmed the structures with mass errors < 3 ppm. Melting points were determined in triplicate using capillary tubes.

### 2.2. In Vitro Tests

#### 2.2.1. Trichomonacidal Assay

Antiparasitic activity was evaluated against the metronidazole-sensitive *T. vaginalis* JH31A#4 isolate (American Type Culture Collection). Trophozoites were cultured in TYM modified medium containing 10% heat-inactivated fetal bovine (FBS) serum and antibiotic solution, at 37 °C and 5% CO_2_. Log-phase parasite cultures were incubated in Pyrex tubes at a concentration of 10^5^ parasites/mL with compounds for 24 h at 37 °C/5% CO_2_. The 1,3-thiazolidin-4-ones, which were dissolved in dimethyl sulfoxide (DMSO), were evaluated at different concentrations ranging from 100 µM to 3.125 µM. The cultures were then transferred to sterile 96-well plates, after which the trichomonacidal activity was determined using the redox dye resazurin (Sigma-Aldrich, St. Louis, MO, USA), as previously described [20]. The experiments were performed at least twice in triplicate. Metronidazole was used in all experiments as positive control. IC_50_ values and 95% confidence intervals were calculated using Probit analysis (SPSS v.20, IBM, Armonk, NY, USA).

#### 2.2.2. Non-Specific Cytotoxic

Cytotoxicity assays were performed on the CCL-81 and HeLa cell lines. Both mammalian cells were maintained in RPMI-1640 medium, supplemented with 10% FBS and antibiotic solution at 37 °C and 5% CO_2_. To determine the non-specific cytotoxicity of thiazolidin-4-ones, the cells were diluted to 100 µL per well and plated in 96-well plates. After a 6 h attachment period, 100 µL of the different compounds were added to the wells. Cell viability was determined after 24 hours’ incubation using the resazurin (stock solution 1 mM) fluorescence method [21]. The cytotoxic profile of the compounds was evaluated at the same concentrations used for antiparasitic determinations. The experiments were performed at least twice in triplicate.

### 2.3. Computational Analysis: In Silico Studies

#### 2.3.1. Bioavailability and Potential Risk Parameters

To predict the oral bioavailability of the molecules, we used the “rule of five” described by Lipinski and colleagues [22] which states that molecules with a Molecular Weight (MW) ≤ 500 Da, an octanol-water coefficient of partition (CLogP) ≤ 5 and no more than 5 hydrogen bond donors (Hd) and 10 hydrogen bond acceptors (Ha) present high oral bioavailability. Molecular weight (MW), H-bond acceptors (Ha), H-bond donors (Hd) and number of rotatable bonds (Rotb) were calculated by Molinspiration Online Property Calculation Toolkit (http://molinspiration.com, accessed on 20 October 2025), while topological polar surface area (PSA), CLogP and distribution coefficient for ionizable (CLogD) molecules were executed using ChemAxon Calculator Plugins (http://chemaxon.com, accessed on 20 October 2025).

The prediction of potential risk associated with mutagenic, tumorigenic, irritant and reproductive side effects was calculated using the computational toxicology approach OSIRIS Property Explorer (https://www.organic-chemistry.org/prog/peo/ accessed on 04 November 2025). Drug-likeness and drug-score were also evaluated using the same tool, which compares the chemical compounds with commercialized drugs [23].

#### 2.3.2. Molecular Docking

All molecular docking and analysis procedures were performed using established open-source software (Supplementary Material). Seven target proteins were selected for this study [24,25,26,27,28,29]. Four structures were obtained from the Protein Data Bank (PDB): Lactate dehydrogenase (LDH) (PDB ID: 5a1t), Triosephosphate isomerase (TPI) (PDB ID: 3qst), Methionine Ƴ-lyase (MGL) (PDB ID: 1e5e), and Purine nucleoside phosphorylase (PNP) (PDB ID: 1z36). Three additional protein structures were modeled using the I-TASSER 5.0 server [17]: Cathepsin-like cysteine protease (CPC), Papain-like cysteine protease (PCP), and Thioredoxin reductase (TrxR).

The prepare receptor script from ADT was used to process each PDB file. This procedure involved removing solvent molecules, adding polar hydrogen atoms, and assigning Gasteiger partial charges. The processed receptors were saved in the PDBQT file format, which includes atom type information required by AutoDock Vina version 1.2.6. [30].

The chemical library with the seven small molecules, identified as compounds **4a**, **4b**, **4c**, **4d**, **4e**, **4f**, and **4g**, were prepared from their initial SDF format. First, Open Babel was used to convert each SDF file into a PDB structure. Subsequently, the prepare ligand script from ADT was employed to generate the final PDBQT files. This step assigned Gasteiger charges, defined rotatable bonds (torsions), and merged non-polar hydrogens to prepare the ligands for flexible docking.

Blind docking was performed for each of the seven ligands against each of the seven receptors using AutoDock Vina. To define the simulation search space, the autobox (https://github.com/omixlab/autobox/ accessed on 8 October 2025) utility was used with the blind flag, which automatically generates a grid box that encompasses the entire surface of the receptor. This approach allows for an unbiased search for potential binding sites. Docking was performed using the default exhaustiveness setting of 8. For each simulation, Vina generated multiple binding poses ranked by their predicted binding affinity (Kcal/mol).

The top-ranked (lowest energy) binding pose for each ligand-receptor complex was selected for detailed interaction analysis. A complex containing the receptor and the top ligand pose was generated using Open Babel. This complex was then analyzed with PLIP to identify and classify all non-covalent interactions, including hydrogen bonds, hydrophobic contacts, salt bridges, and π-stacking. PLIP generated a detailed report in XML format for each complex. A custom Python (version 3.9) script was developed to parse the docking results and PLIP reports. The script extracted the binding energy for the top pose directly from Vina’s output PDBQT file and calculated the Ligand Efficiency (LE) by dividing the binding energy by the number of heavy atoms [31,32,33].

The most specific interactions between compounds and enzymes were illustrated with Discovery Studio version v25.1. (Dassault Systèmes BIOVIA; San Diego, CA, USA).

## 3. Results and Discussion

The novel thiazolidinones reported in this work were synthesized via MCR using a one-pot methodology, starting from 3,5-di-*tert*-butyl-4-hydroxybenzaldehyde (an analog of the industrial antioxidant BHT), as shown in Scheme 1. Reaction progress was monitored by TLC. The structures of the obtained compounds were confirmed by MS and ^1^H/^13^C NMR spectroscopy.

The compounds were detected by HRMS; however, the predominant signals correspond to combinations of fragment ions rather than intact molecular ions. This indicates that in-source fragmentation may occur, and that the major observable ions are formed through fragment–fragment associations generated during ionization. This behavior was observed for all compounds derived from 3,5-di-*tert*-butyl-4-hydroxybenzaldehyde, suggesting a structural influence on ion stability under the experimental conditions.

Regarding the in vitro antiparasitic effect, compounds **4a**, **4c**, **4f**, and **4g** exhibited activity of >90% at the highest tested concentration. Meanwhile, **4a**, **4b** and **4f** maintained remarkable trichomonacidal activity, with IC_50_ values of 18.89, 16.13 and 22.04 µM, respectively (Table 1).

None of the derivatives showed cytotoxicity against the two cell lines (Vero CCL-81 and HeLa) in cytotoxicity tests, so a value of >100 µM was taken as the CC_50_ for determining the selectivity index, depicted in Table 1.

Although any of the compounds of this series showed a better activity than metronidazole, compounds with SI > 4.5 were submitted to virtual screening analysis to determine their suitability to be orally administered. In addition to a given compound’s antiparasitic and specific activity, it is essential to understand its other properties, such as those related to its absorption, distribution, metabolism and excretion (ADME). Many molecules that show excellent results in vitro subsequently fail in vivo due to impaired bioavailability and toxicity. Consequently, the in silico study of these characteristics can offer interesting information for the rational synthesis of new derivatives with better characteristics and using an inexpensive tool. The Lipinski’s “rule of five” states that a poor absorption or permeability are present in molecules with a molecular weight (MW) > 500 Da, a log partition coefficient (cLogP) > 5, more than five hydrogen bond donor groups and more than ten hydrogen bond acceptor groups [22]. If one of these conditions is not complied with, the permeability and oral absorption is considered acceptable; however, if two or more properties are out of range, this indicates poor bioavailability. The physicochemical characteristics of the most active synthetic derivatives against *T. vaginalis* (Table 1) have been determined. In addition, the 1,3-thiazolidin-4-one scaffold, from which this series has been synthetized (Figure 1), has also been calculated. As depicted in Table 2, the 1,3-thiazolidin-4-one scaffold is characterized by its low molecular weight and high hydrophilicity, as demonstrated by its cLogP value < 0. Moreover, the absence of rotatable bonds gives rise to a remarkable degree of structural rigidity, thereby minimizing the entropic penalty experienced during receptor binding. These attributes render it an excellent scaffold for the development of the current chemical series. Moreover, compounds **4a** and **4b** exhibit cLogP values of 6.98 and 6.37, respectively, which exceed the recommended threshold and indicate high lipophilicity. Elevated lipophilicity has been associated with reduced aqueous solubility, altered pharmacokinetics, and increased risk of toxicity [34,35]. This property is probably one of the reasons why these compounds have low drug-score values (Table 3) and may pose challenges such as reduced aqueous solubility and potential alterations in absorption and distribution; it does not preclude their development as drug candidates. Instead, it highlights the need for careful optimization strategies, such as formulation approaches or structural modifications, to balance potency with pharmacokinetic properties [36]. Moreover, the determination of Polar surface area (PSA), which is related to the drug’s capacity to form hydrogen bonds, shows values PSA < 140 Å^2^ which are also related with adequate absorption in human intestine [37]. Therefore, despite the elevated lipophilicity, other parameters remain within acceptable ranges, suggesting that these compounds could serve as promising leads for further refinement.

Compliance with the “rule of five” and an adequate number of rotatable bonds (RotB ≤ 10) suggest that these molecules present an ideal balance of size, polarity, fat solubility and flexibility, enabling them to be successfully absorbed and interact efficiently with their target [38].

In terms of computational studies, toxicity prediction shows that there are no mutagenic, tumorigenic or irritant risks. However, compound **4f** presents a high risk of reproductive effects (Table 3). Compound **4f** differs from **4b** not only by replacement of a phenyl ring with a benzimidazole moiety, but also by the introduction of additional heteroatoms and hydrogen-bonding functionality, which substantially alters its physicochemical profile [22,37]. These differences are reflected in the predicted drug-likeness parameters. Although several derivatives exhibit negative drug-likeness scores, reflecting some structural divergence from currently approved drugs, this observation is consistent with the exploratory nature of early-stage antiparasitic lead discovery. Importantly, these results highlight valuable opportunities for rational structural optimization. Overall, the predicted profiles support further refinement of the most promising ligands, aiming to enhance both biological activity and drug-like properties.

In the molecular docking analysis, the number of heavy atoms (non-hydrogen atoms), interaction residues, binding energy (where more negative values indicate stronger binding) and binding efficiency per heavy atom (useful for comparing compounds of different sizes), were evaluated. Table 4 shows all the interactions between the seven 1,3-thiazolidin-4-ones and the seven parasitic enzymes.

**Figure 2:**
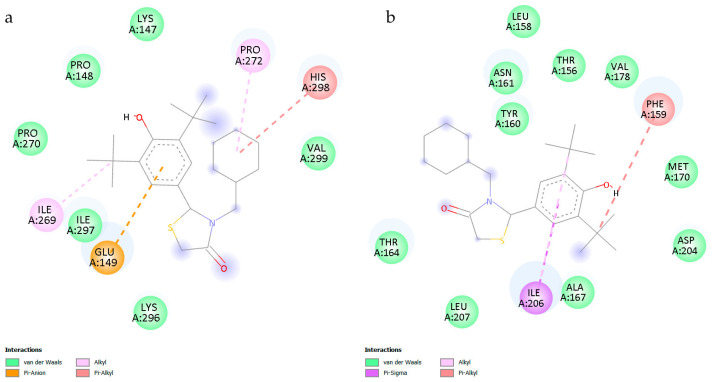
Representation of 2D projection of main interactions between compound **4a** with the *T. vaginalis* enzymes LDH (**a**) and PNP (**b**).

In general, the molecular docking analyses were performed as an exploratory, hypothesis-generating approach, and the compounds demonstrated a clear preference for the most relevant evaluated targets in *T. vaginalis*, except for thioredoxin reductase (TrxR). Compound **4c** stood out in its interaction with lactate dehydrogenase (LDH) (−7.19 kcal/mol), engaging with critical residues, such as Glu149 within the catalytic pocket, involved in energy metabolism [39]. It also engages with Ile269 and Pro272, which are essential for the functionality of the catalytic site, contributing to a stable binding pose within the active site [40,41] (Figure 2a). Lactate dehydrogenase (LDH) catalyzes the conversion of lactate to pyruvate and the conversion of NAD^+^ to NADH. It is a key enzyme in glycolysis and is found in almost all living cells. TvLDH from *T. vaginalis* is essential for the parasite’s survival; however, it differs significantly from human LDH, making it a promising target for drug discovery [39,42]. Nevertheless, the docking results reported here should be interpreted only as indicative of a possible molecular interaction, and not as direct evidence of enzymatic inhibition.

Furthermore, in the PNP, **4a** interacts with both Phe159 and Ile206 (Figure 2b). These interactions provide stability and contribute to maintaining the ligand within the active site of purine nucleoside phosphorylase (PNP). This enzyme catalyzes the phosphorolysis of N-glycosidic bonds in purine nucleosides, producing α-ribose-1-phosphate and the purine base. It acts in the salvage pathway, which is essential for obligated parasitic protozoa [43,44]. While these results suggest that PNP represents a plausible molecular target, any interference with the purine salvage pathway should be regarded as a hypothesis and requires experimental validation [44].

**Figure 3:**
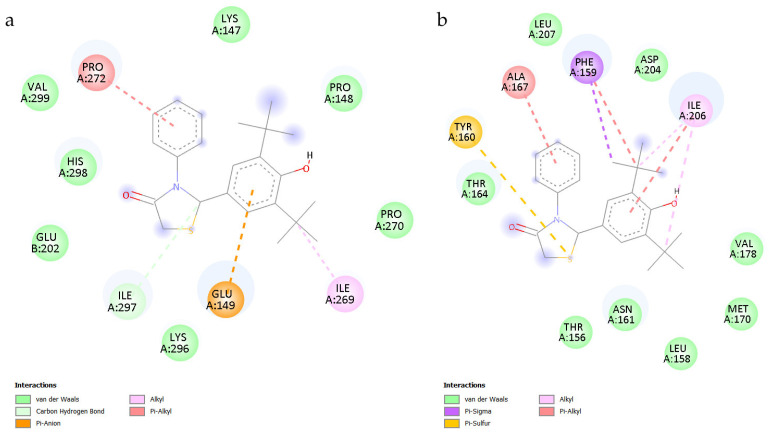
Representation of 2D projection of main interactions between compound 4b with the *T. vaginalis* enzymes LDH (**a**) and PNP (**b**).

Compound **4b** also showed robust interactions with parasitic metabolic enzymes PNP and LDH, showing adequate ΔG (−7.5 kcal/mol and −7.16 kcal/mol, respectively). This derivative interacts with the same residues of LDH as **4a** (Figure 3a) while in PNP, **4b** interacts not only with Phe159 and Ile206, but also with Tyr160 and Ala167 (Figure 3b). These similarities reinforce the consistency of the predicted binding modes among structurally related derivatives.

**Figure 4:**
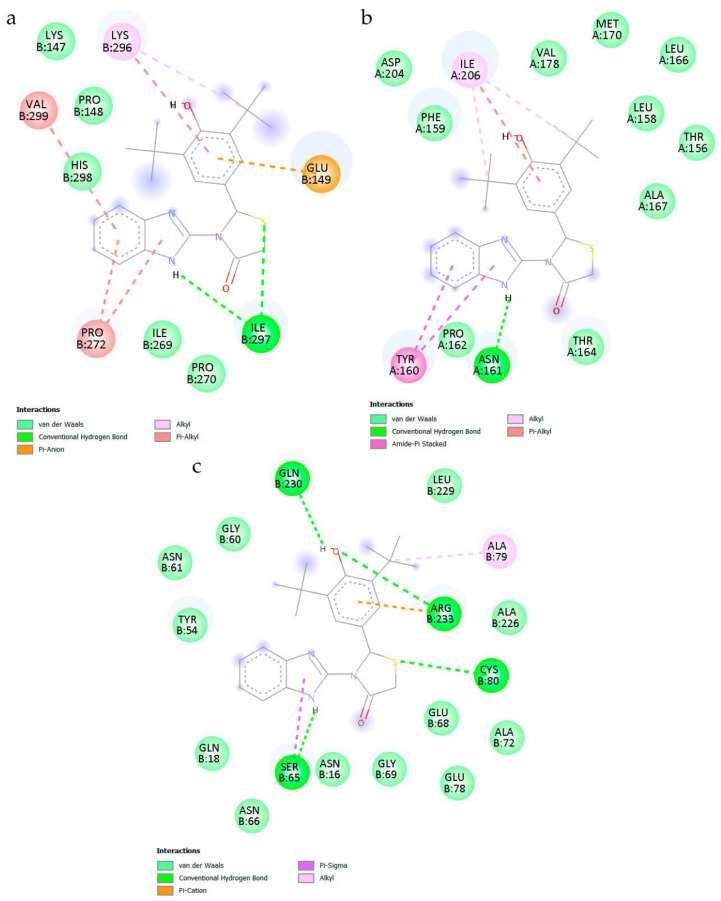
Representation of 2D projection of main interactions between compound **4f** with the *T. vaginalis* enzymes LDH (**a**) PNP (**b**) and MGL (**c**).

Compound **4f** emerged as the most promising ligand, showing remarkable binding energies against PNP (−8.44 kcal/mol), methionine Ƴ-lyase (MGL) (−7.68 kcal/mol) and LDH (−7.53 kcal/mol) (Figure 4). It is worth mentioning the strong interaction with MGL, through interactions with Gln230, Ala79, Arg233, Cys80 and Ser65. MGL is an enzyme involved in sulfur-containing amino acid metabolism and has been associated with *Trichomonas* physiology and virulence; however, the present docking results should be interpreted as preliminary and hypothesis-driven, rather than as confirmation of protease-dependent virulence modulation. Furthermore, this enzyme is an interesting target related to *Trichomonas* virulence, given that it is not present in mammals [27,45,46].

Docking applied to *T. vaginalis* targets, including LDH and PNP, has demonstrated that derivatives with similar structure tend to establish similar interactions. In this case, **4a**, **4b** and **4f** are effectively positioned within the active site through hydrogen and hydrophobic bonding, which may contribute to stable binding within the catalytic pocket, thereby supporting a potential multi-target interaction profile rather than a single dominant mechanism of action [47].

Molecular hybridization is an effective strategy for generating compounds with complementary biological properties and has been widely applied in drug design to improve efficacy while reducing development costs [16]. Accordingly, combining multiple heterocyclic motifs within a single molecule, such as the 1,3-thiazolidin-4-one core, represents a promising approach for the development of novel pharmaceuticals. A few interactions observed with the sulfur atom were identified in the present docking analysis, like π-sulfur interactions and conventional hydrogen bonding (Figure 3b and Figure 4a,c). Clinically, antiparasitic agents such as thiabendazole (Figure 1) demonstrate that thiazole-based systems can contribute significantly to biological activity through scaffold and orientation effects rather than isolated binding interactions [18]. Docking evaluation of thiabendazole bound with mitochondrial succinate:ubiquinone oxidoreductase shows that both nitrogens present in the benzimidazole structure form interactions, and its thiazole group interacts with the protein [48]. Compound **4f** also shows this pattern for LDH (Figure 4a) as well as interactions with benzimidazole structure (Figure 4b,c).

In the present study, incorporation of a BHT-derived moiety was not intended to explicitly generate dual-action antioxidant–antiparasitic agents, but rather to probe the impact of a bulky, lipophilic phenolic substituent [14,15] on the antiparasitic performance of a thiazolidin-4-one scaffold. This molecular hybridization strategy was aimed at modulating physicochemical properties such as lipophilicity, membrane affinity, and conformational behavior. Within this framework, the thiazolidin-4-one ring was employed as (i) a privileged scaffold, (ii) a structural organizer, and (iii) a bioisosteric analog of thiazole-containing pharmacophores, such as those found in thiabendazole (Figure 1) [10,17].

A few studies have reported thiazolidine–BHT hybrids displaying dual biological activity. Examples include iminothiazolidinones and thiazolidinediones acting as 5-lipoxygenase and cyclooxygenase inhibitors with anti-inflammatory activity [15]. Likewise, new calcium antagonists with combined calcium-overload inhibition and antioxidant properties have been synthesized using rational design strategies such as molecular hybridization [14]. In this context, the compounds synthesized in the present study incorporate both 1,3-thiazolidin-4-one and BHT motifs, BHT being a well-known synthetic antioxidant extensively studied for its free-radical scavenging properties [49]. Regarding antiparasitic activity, thiazolidin-4-ones are well documented for their promising antiprotozoal properties. Multiple reports describe this chemical class evaluated against *Toxoplasma gondii* and *Trypanosoma* species [17,50,51,52]. The structure–activity relationship (SAR) observed in this work supports the intrinsic bioactivity of the thiazolidin-4-one scaffold, while mechanistic interpretations remain hypothesis-driven in the absence of functional validation.

Although the limited size of the compound set precludes definitive SAR conclusions, the observed differences in trichomonacidal activity among compounds **4a**–**g** suggest that substitution at the nitrogen position of the 1,3-thiazolidin-4-one core may influence biological performance. However, these effects cannot be attributed to a single structural parameter, as the N-substituents examined in this study differ simultaneously in size, polarity, aromaticity, and conformational flexibility, making it difficult to isolate the contribution of individual factors [10,13,17,53]. Consequently, the present SAR analysis should be regarded as preliminary and qualitative, providing directional insights that may guide the design of more systematically varied analogs in future studies. Nevertheless, these findings highlight the potential of the thiazolidin-4-one scaffold as a promising framework for the development of new trichomonacidal agents.

This is illustrated by the trichomonacidal activity of compounds **4b** and **4a**, which bear a simple aromatic (phenyl) and aliphatic (cyclohexylmethyl) substituent, respectively, and exhibit IC_50_ values below 20 μM. The activity of **4b** is consistent with previous studies showing that aromatic rings can enhance biological interactions through π-based or hydrophobic interactions with molecular targets [10,53]. In contrast, the comparable activity observed for **4a** indicates that nonpolar aliphatic chains do not necessarily diminish the biological performance of thiazolidine-4-one derivatives and may even contribute favorably. This effect may be related to the increased conformational flexibility imparted by the aliphatic group, which could facilitate adaptation to the binding environment [54]. Similar findings have been reported for aliphatic 1,3-thiazolidin-4-one derivatives displaying antifungal activity with low cytotoxicity [55].

Compound **4c**, bearing a benzodioxole (3,4-methylenedioxyphenyl) substituent, represents an aromatic but non-nitrogen-containing N-substitution pattern. Non-aromatic and aromatic cyclic substituents have been shown to influence bioactivity by modulating hydrophobicity, conformational dynamics, and intermolecular interactions, as commonly observed in heterocycle-based medicinal chemistry optimization studies [10,51,56]. For example, Dofuor et al. (2019) evaluated norlignan-derived structures against *Trypanosoma* species that share partial structural similarity (3,4-methylenedioxy motif) with compound **4c** [57]. Related chemical motifs have also been explored in thiosemicarbazone derivatives [58], and comparable behavior was observed for **4c** in the present study. When following the conditions reported by Masteloto et al. (2015) [19] for the synthesis of the 3,4-(methylenedioxy)aniline-derived thiazolidine-4-one **4c**, its yield increased significantly from 27% to 80%. The improved yield resulted from adjust ting the stoichiometry (aldehyde:amine:mercaptoacetic acid = 1:1:3) and adding the acid after imine formation instead of at the start.

Compound **4f** (IC_50_ = 22.04 μM) contains a benzimidazole substituent and is structurally similar to thiabendazole (Figure 1). Benzimidazoles are widely recognized antiparasitic pharmacophores; thiabendazole is a classical example, in which the thiazole serves as the aromatic analog of the thiazolidine-4-one ring fused to a benzimidazole [59]. However, substitution with other azoles (compounds **4d** or **4g**) markedly reduced antiparasitic activity. Compound **4f** would benefit from further optimization to improve yield (29%), as its low solubility—precipitating even in hot toluene—likely contributed to reduced synthetic efficiency. Due to its structural similarity to thiabendazole, this compound remains of particular interest [18].

Sawant et al. (2020) also synthesized benzimidazole-substituted thiazolidin-4-ones, reporting moderate to low yields (67–40%) using microwave irradiation in ethanol as a polar solvent, with a reaction time of only 30 min [60]. In contrast to the present study, those derivatives required flash chromatography for purification, whereas **4f** precipitated naturally upon workup. These observations suggest that microwave-assisted synthesis in the presence of mercaptoacetic acid and a polar solvent could provide a more efficient route to **4f** in future work, especially given its limited solubility in toluene.

Future studies will focus on expanding the compound library with more systematically varied N-substituents, evaluating antioxidant properties where relevant, and exploring alternative synthetic conditions to improve yields and solubility, particularly for benzimidazole-containing derivatives such as **4f**.

## 4. Conclusions

Finally, considering the docking results, it can be concluded that strong binding affinity is combined with relevant interactions with essential enzyme residues of *T. vaginalis* by compound **4f,** while greater efficiency is offered by **4b** and a balanced profile is presented by **4a**, targeting both energy metabolism and nucleotide recovery pathways.

The structural versatility, promising antiparasitic activity, adequate physicochemical properties and ease of synthesis of 1,3-thiazolidin-4-ones make them a compelling class of interesting compounds for parasitic disease research. This study is the first to report the in vitro and in silico activity of 1,3-thiazolidin-4-one derivatives against *T. vaginalis*. The promising in vitro activity results observed for compounds **4b**, **4a** and **4f** (showing IC_50_ < 20 µM) and the favorable docking interactions with essential parasitic enzymes highlight the potential of these molecules for further development.

## 5. Patents

The chemical compounds published in this study are covered by the patent “New effective compounds against *Trichomonas* spp.; synthesis and trichomonacidal activity of 1,3-thiazolidin-4-ones” in compliance with Decree No. 7,724/2012, registered under process number 233110041124/2025-60.

## Figures and Tables

**Table 1:** Trichomonacidal activity (effect at 100 µM and IC_50_), non-specific cytotoxicity against Vero and HeLa cells (% cytotoxicity at 100 µM) and selectivity indexes (SI) of the 1,3-thiazolidin-4-ones.

Comp.	Antiparasitic Effect		Non-Specific Cytotoxicity ^b^		SI ^c^
	% (100 µM)	IC_50_ (µM) ^a^	Vero CCL-81	HeLa	
**4a**	93.85 ± 4.57	18.89 ± 1.56 [13.45–26.87]	0	0.50 ± 0.70	>5
**4b**	82.74 ± 9.30	16.13 ± 0.79 [13.06–19.82]	13.40 ± 0.89	0	>6
**4c**	95.51 ± 2.45	26.32 ± 0.70 [23.58–29.53]	0	0	>3.8
**4d**	37.71 ± 7.94	>100	ND	0	
**4e**	70.52 ± 6.71	30.43 ± 1.57 [24.80–38.33]	0.42 ± 0.49	7.14 ± 1.39	>3.3
**4f**	94.19 ± 4.10	22.04 ± 1.85 [16.00–30.95]	0	2.69 ± 0.27	>4.5
**4g**	95.06 ± 1.01	55.47 ± 3.88 [42.81–76.20]	0	0	>1.8
MTZ	100	0.8 ± 0.3 [0.66–0.99]	0	0	>100

^a^ Mean value, in square brackets, 95% confidence intervals. ^b^ Mean values and standard deviation of three independent experiments. ^c^ Selectivity indexes (SI = CC_50_ mammalian cells/IC_50_ *T. vaginalis*).

**Table 2:** Bioavailability based on in silico determination of physicochemical properties of the scaffold 1,3-thiazolidin-4-one and its most active derivatives.

Comp.	MW (g/mol)	cLogP	Hd	Ha	Lipinski’s Violation	PSA	RotB
1,3-thiazolidin-4-one	103.15	–0.21	1	1	0	29.10	0
**4a**	403.63	6.98	1	3	1	40.54	4
**4b**	383.55	6.37	1	3	1	40.54	5
**4f**	423.58	5.79	2	5	1	69.22	4

MW: molecular wight; cLogP: octanol/water partition coefficient; Hd: hydrogen donors; Ha: hydrogen acceptors; PSA: topological polar surface area; RotB: rotatable bonds.

**Table 3:** Toxicity risk profile, drug-likeness and drug-score parameters.

Comp.	Toxicity Risk Profile				Drug-Likeness	Drug-Score
	Mut.	Tum.	Irrit.	Reprod.eff		
**4a**	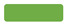	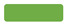	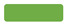	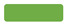	−16.02	0.21
**4b**	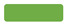	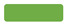	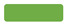	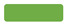	−11.82	0.21
**4f**	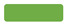	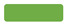	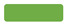	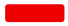	−11.55	0.11

Mut.: mutagenic; Tum.: tumorigenic; Irrit.: irritant; Reprod.eff.: reproductive effect. Green: no risk; red: high risk.

**Table 4:** Binding affinity and aminoacidic interactions of the 1,3-thiazolidin-4-one derivatives at the active site of different *T. vaginalis* enzymes.

Compound	Receptor	Number of Heavy Atoms	Binding Energy (Kcal/mol)	Ligand Efficiency ΔG (Kcal/mol)	Interactions
**4a**	CPC	29	−5.46	−0.188	Ala10, Trp34, Lys38, Val41, Ala54, Leu55
	TPI	29	−6.71	−0.231	Phe73, Glu76, Glu103, Phe107
	LDH	29	−7.19	−0.248	Pro148, Ile269, Pro270, Lys296, Ile297, Val299
	MGL	29	−6.83	−0.236	Gln18, Tyr30, Phe35, Tyr54, Pro62
	PCP	29	−5.79	−0.200	Arg47, Phe52, Met54, Asp229
	PNP	29	−7.15	−0.247	Phe159, Tyr160, Asn161, Val178, Ile206
	TrxR	29	−3.69	−0.127	
**4b**	CPC	28	−6.12	−0.219	Ala10, Trp14, Trp34, Lys38, Leu55
	TPI	28	−6.54	−0.234	Lys11, Ala12, Pro43, Glu96, Arg97
	LDH	28	−7.16	−0.256	Pro148, Ile269, Pro272, Ile297
	MGL	28	−6.35	−0.227	Gln18, Asp20, Pro28, Tyr30, Phe35, Tyr54, Pro62
	PCP	28	−6.46	−0.231	Pro82, Lys84, Asn85
	PNP	28	−7.50	−0.268	Thr156, Phe159, Thr164, Ala167, Val178, Ile206
	TrxR	28	−3.28	−0.117	
**4c**	CPC	31	−6.33	−0.204	Ala10, Trp14, Trp34, Lys38, Leu55
	TPI	31	−7.21	−0.233	Asn9, Ala12, Phe44, Glu63, His94, Glu96, Arg97, Leu101
	LDH	31	−7.74	−0.249	Pro148, Glu149, Ile269, Pro272, Ile297, Val299
	MGL	31	−6.90	−0.223	Gln18, Asp20, Pro28, Tyr30, Phe35, Tyr54, Pro62
	PCP	31	−6.83	−0.220	Pro82, Lys84, Asn85, Phe95, Tyr157
	PNP	31	−7.59	−0.245	Phe159, Val178, Ile206
	TrxR	31	−3.43	−0.111	
**4d**	CPC	28	−5.99	−0.214	Ala10, Trp34, Lys38, Leu55
	TPI	28	−6.25	−0.223	Lys11, Ala12, Phe44, Glu63, His94, Glu96, Arg97, Leu101
	LDH	28	−6.91	−0.247	Lys175, Gln250, Glu263, Val264
	MGL	28	−7.21	−0.257	Tyr288, Glu296
	PCP	28	−6.51	−0.232	Glu23, His24, Ala27, Leu29, Trp208, Ser209, Glu220
	PNP	28	−7.56	−0.270	Thr156, Phe159, Val178, Ile206
	TrxR	28	−3.18	−0.114	
**4e**	CPC	28	−5.40	−0.193	Ala10, Trp34, Lys38, Val41, Ala54, Leu55
	TPI	28	−6.67	−0.238	Lys11, Phe44, Glu63, His94, Glu96, Arg97, Leu101
	LDH	28	−6.93	−0.247	Glu149, Lys296, Ile297, His298, Val299
	MGL	28	−6.72	−0.24	Ala135, Thr160, Lys162, Tyr288
	PCP	28	−6.62	−0.236	Pro82, Lys84, Glu152
	PNP	28	−7.07	−0.252	Phe159, Tyr160, Val178, Ile206
	TrxR	28	−3.19	−0.114	
**4f**	CPC	32	−7.01	−0.219	Leu55, Asn56, Lys57
	TPI	32	−7.01	−0.219	Lys11, Pro43, Glu63, His94, Glu96, Arg97, Leu101
	LDH	32	−7.54	−0.235	Lys147, Pro148, Glu149, Pro272, Lys296, Val299
	MGL	32	−7.68	−0.240	Asn16, Ala79, Ala226, Gln230, Arg233
	PCP	32	−7.11	−0.222	Pro82, Val83, Lys84, Glu254
	PNP	32	−8.44	−0.264	Thr156, Phe159, Tyr160, Asn161, Val178, Ile206
	TrxR	32	−3.88	−0.121	
**4g**	CPC	36	−6.69	−0.186	Glu78, Lys80, Ala157, Asp160, Lys164, Tyr199
	TPI	36	−7.15	−0.199	Lys11, Pro43, Phe44, Val45, His94, Glu96, Arg97
	LDH	36	−8.09	−0.225	Pro148, Ile269, Pro270, Pro272, Val299
	MGL	36	−7.49	−0.208	Asn16, Gly53, Tyr54, Ile55, Leu59
	PCP	36	−7.29	−0.202	Pro82, Lys84, Phe95, Glu152, Tyr157
	PNP	36	−7.60	−0.211	Thr111, Asn116, Arg117, Phe173, Phe175
	TrxR	36	−3.58	−0.099	

CPC: Cathepsin cysteine protease, TPI: Triosephosphate isomerase, LDH: lactate dehydrogenase, MGL: methionine Ƴ-lyase, PCP: papain-like cysteine proteinase, PNP: purine nucleoside phosphorylase, TrxR: thioredoxin reductase.

## Data Availability

The raw data supporting the conclusions of this article will be made available by the authors on request.

## Supplementary Materials

The following supporting information can be downloaded at: https://www.mdpi.com/article/10.3390/pharmaceutics18010110/s1, Figure S1: Chromatogram, mass spectra and fragmentation spectra of compound **4a**; Figure S2: ^1^H and ^13^C NMR spectra of compound **4a**; Figure S3: HRMS (ESI^+^) of compound **4a** showing the [M + H]^+^ ion and adducts from fragmentation; Figure S4: Chromatogram, mass spectra and fragmentation spectra of compound **4b**; Figure S5: ^1^H and ^13^C NMR spectra of compound **4b**; Figure S6: HRMS (ESI^+^) of compound **4b** showing the [M + H]^+^ ion and adducts from fragmentation; Figure S7: Chromatogram, mass spectra and fragmentation spectra of compound **4c**; Figure S8: ^1^H and ^13^C NMR spectra of compound **4c**; Figure S9: HRMS (ESI^+^) of compound **4c** showing the [M + H]^+^ ion and adducts from fragmentation; Figure S10: Chromatogram, mass spectra and fragmentation spectra of compound **4d**; Figure S11: ^1^H and ^13^C NMR spectra of compound **4d**; Figure S12: HRMS (ESI^+^) of compound **4d** showing the [M + H]^+^ ion and adducts from fragmentation; Figure S13: Chromatogram, mass spectra and fragmentation spectra of compound **4e**; Figure S14: ^1^H and ^13^C NMR spectra of compound **4e**; Figure S15: HRMS (ESI^+^) of compound **4e** showing the [M + H]^+^ ion and adducts from fragmentation; Figure S16: Chromatogram, mass spectra and fragmentation spectra of compound **4f**; Figure S17: ^1^H and ^13^C NMR spectra of compound **4f**; Figure S18: COSY, HSQC and HMBC NMR spectra of compound **4f**; Figure S19: HRMS (ESI^+^) of compound **4f** showing the [M + H]^+^ ion and adducts from fragmentation; Figure S20: Chromatogram, mass spectra and fragmentation spectra of compound **4g**; Figure S21: ^1^H and ^13^C NMR spectra of compound **4g**; Figure S22: HRMS (ESI^+^) of compound **4g** showing the [M + H]^+^ ion and adducts from fragmentation.
