# Supplementary material for: Novel Hit Compounds Against a Neglected Sexually Transmitted Infection: Synthesis and Trichomonacidal Activity of 1,3-Thiazolidin-4-One Derivatives
Source: Pharmaceutics. 2026 Jan 15;18(1):110. doi: 10.3390/pharmaceutics18010110 (PMC12845368; doi:10.3390/pharmaceutics18010110)
Supplement: Supplementary file 1 [file pharmaceutics-18-00110-s001.zip › pharmaceutics-4043407-supplementary.pdf]

Novel Hit Compounds Against a Neglected Sexually Transmitted Infection: Synthesis and Trichomonacidal Activity of 1,3-Thiazolidin-4-One Derivatives

SUPPLEMENTARY MATERIAL

*1. GC, MS, ^{1}H and ^{13}C NMR, and HRMS spectra of compounds **4**.*

1.1. 3-(Cyclohexylmethyl)-2-(3,5-di-tert-butyl-4-hydroxyphenyl)thiazolidin-4-one **4a**

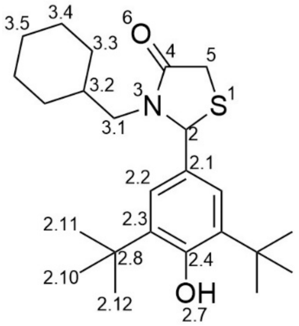

$C_{24}H_{37}NO_2S$, M.W.: 403.63 g/mol, CLogP: 6.98, light orange solid, yield: 0.1993 g (49%), m.p.: 132-134ºC.

TLC System: 7:3 Hexane:Ethyl Acetate. Rf: 0.81

^{1}H NMR (400 MHz, $CDCl_3$): δ (ppm, $J_{H\text{–}H}$ = Hz): 7.07 (s, 2H, H2.2, H2.6), 5.56 (s, 1H, H2), 5.33 (s, 1H, H2.7), 3.79 (dd, 2J = 15.48, 4J = 1.93, 1H, H5a), 3.69 (d, 2J = 15.46, 1H, H5b), 3.42 (dd, 2J = 13.72, 3J = 8.32, 1H, H3.1a), 2.55 (dd, 2J = 13.72, 3J = 6.52, 1H, H3.1b), 1.73 – 1.52 (m, 6H, cyclohexyl), 1.43 (s, 18H, H2.10-H2.15), 1.18 – 1.07 (m, 3H, cyclohexyl), 0.97 – 0.81 (m, 2H, cyclohexyl).

^{13}C NMR (101 MHz, $CDCl_3$) δ (ppm): 171.5 (C4), 154.5 (C2.4), 136.6 (2C, C2.3, C2.5), 129.7 (C2.1), 124.0 (2C, C2.2, C2.6), 65.1 (C2), 49.2 (C3.1), 35.8 (1C, cyclohexyl), 34.5 (2C, C2.8, C2.9), 33.1 (C5), 31.1 (1C, cyclohexyl), 30.6 (1C, cyclohexyl), 30.4 (6C, C2.10-C2.15), 26.4 (1C, cyclohexyl), 26.0 (1C, cyclohexyl), 25.8 (1C, cyclohexyl).

GC: T_R = 14.7 minutes.

MS (70 eV): m/z (%) = 403 (M^+, 15), 370 (36), 328 (17), 307 (9), 260 (52), 250 (13), 234 (9), 219 (9), 110 (16), 102 (10), 67 (10), 57 (100), 55 (66), 43 (11), 41 (50).

HRMS (ESI) m/z: $[M + H]^+$ calculated exact mass (Trace Finder) for $C_{24}H_{37}NO_2S$ = 404.2617, found = 404.2615.

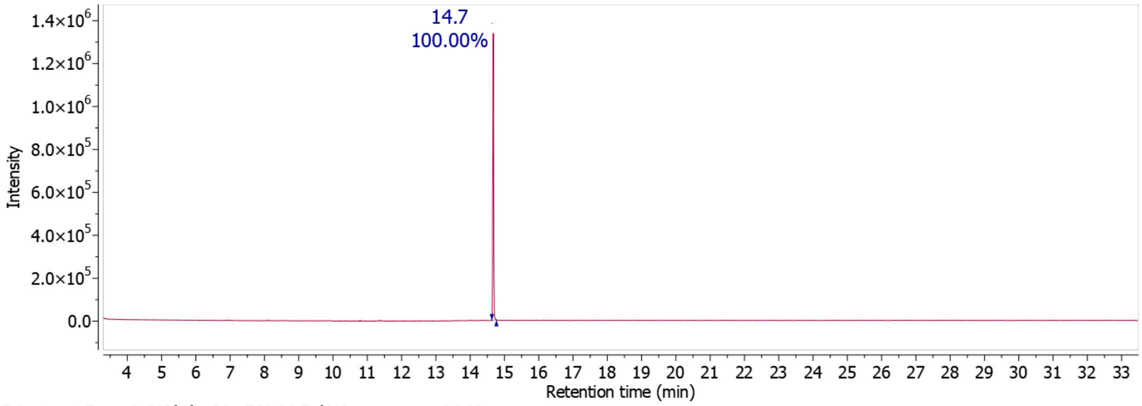
14.7
100.00%
Intensity
Retention time (min)

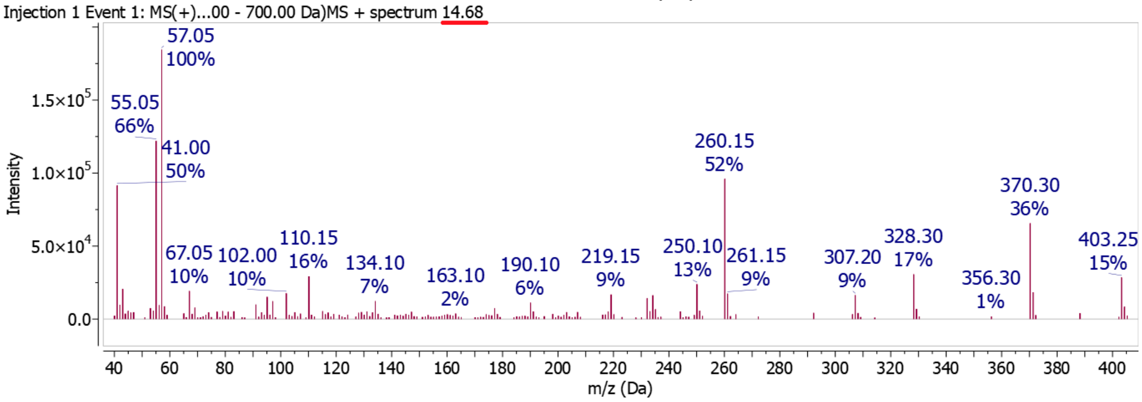
Injection 1 Event 1: MS(+)...00 - 700.00 Da)MS + spectrum 14.68
57.05 100%
55.05 66%
41.00 50%
67.05 10%
102.00 10%
110.15 16%
134.10 7%
163.10 2%
190.10 6%
219.15 9%
250.10 13%
260.15 52%
261.15 9%
307.20 9%
328.30 17%
356.30 1%
370.30 36%
403.25 15%
Intensity
m/z (Da)

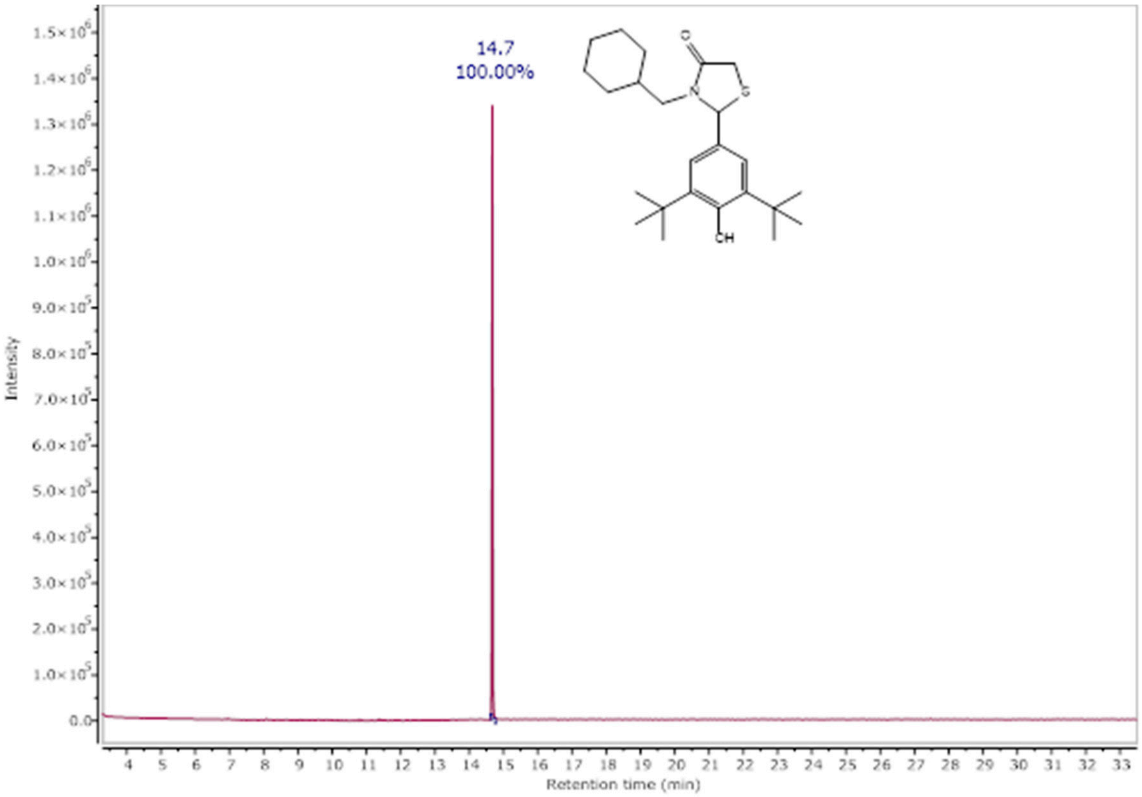
14.7
100.00%
Intensity
Retention time (min)

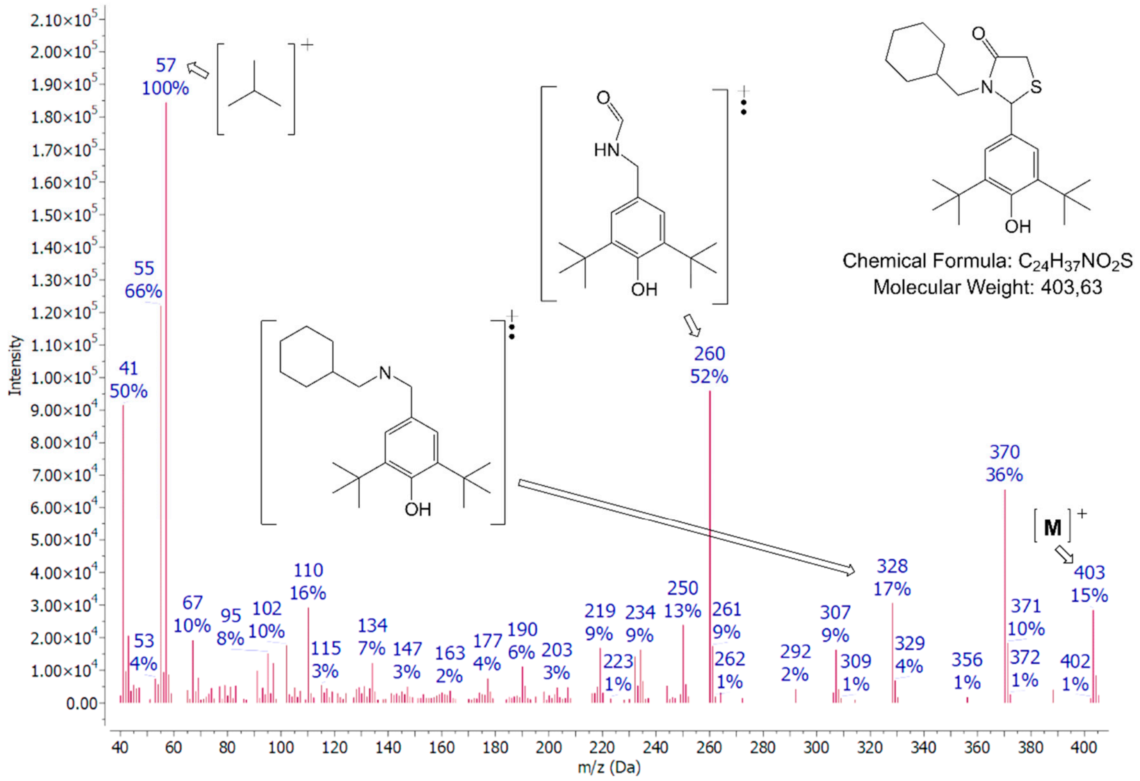

Figure S1. Chromatogram, mass spectra and fragmentation spectra of compound **4a**.

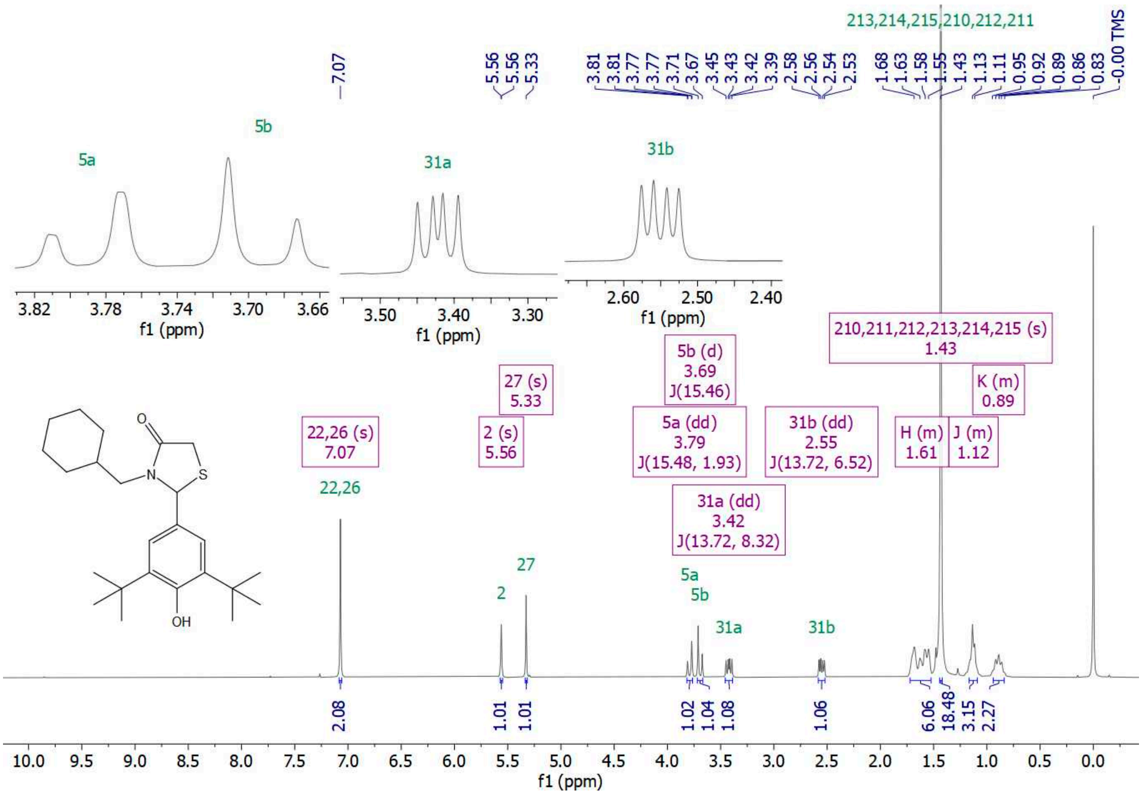

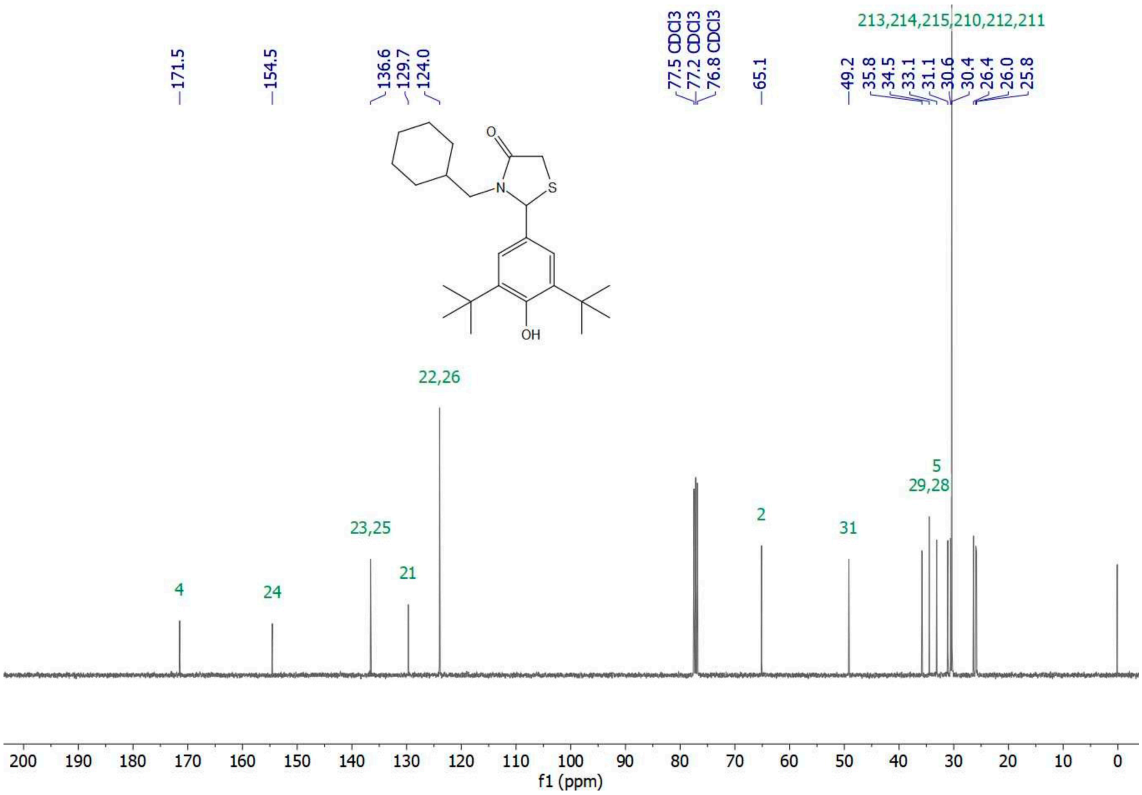

Figure S2. ^{1}H and ^{13}C NMR spectra of compound **4a**.

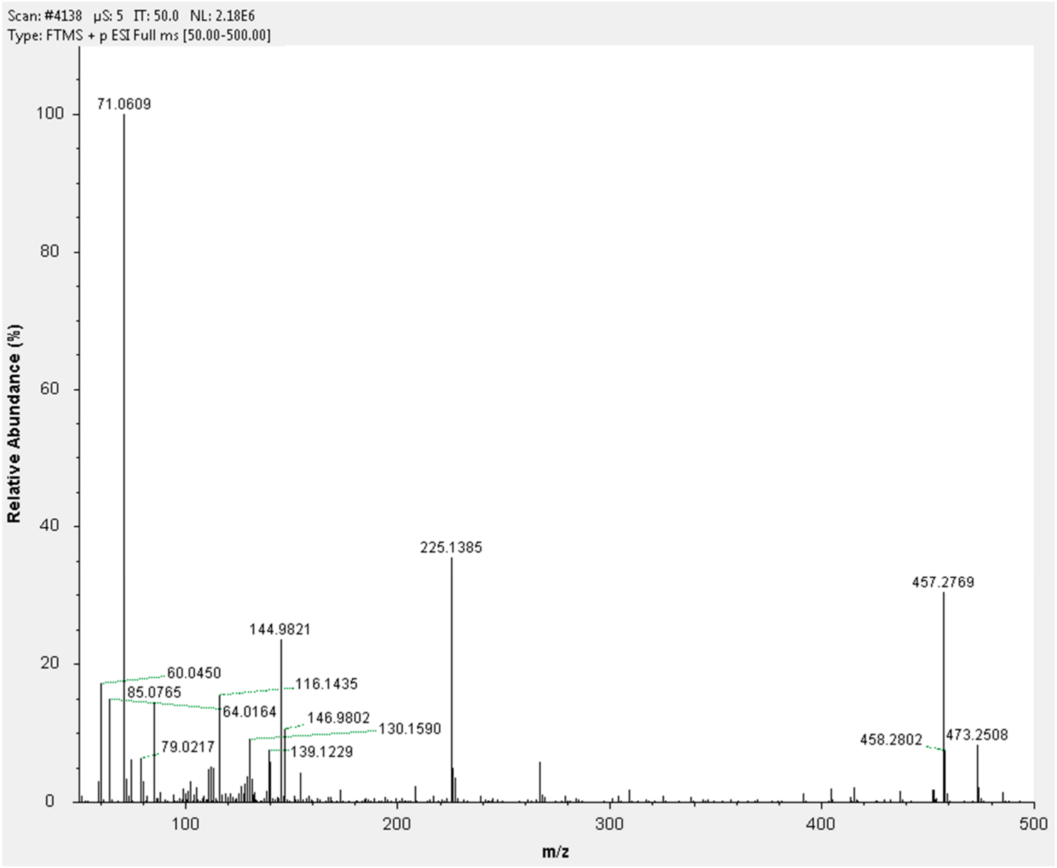

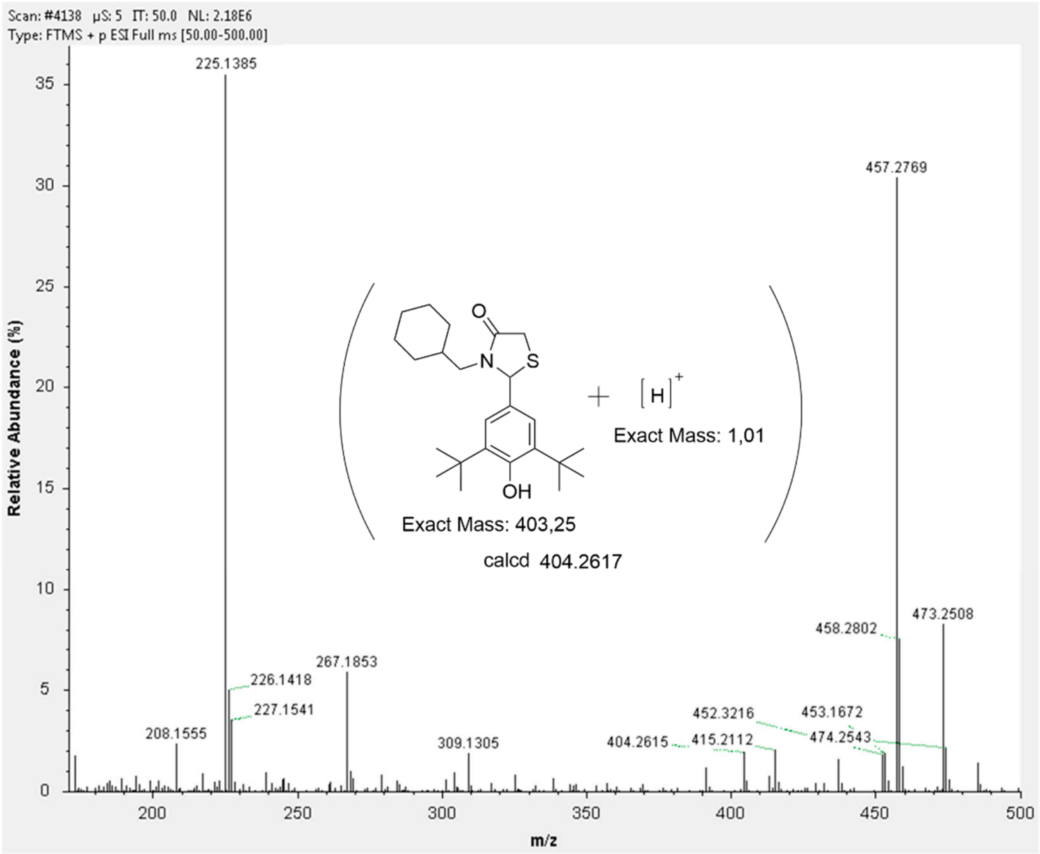

Figure S3. HRMS (ESI+) of compound **4a** showing the [M + H]+ ion and adducts from fragmentation.

1.2. 2-(3,5-Di-tert-butyl-4-hydroxyphenyl)-3-phenylthiazolidin-4-one **4b**

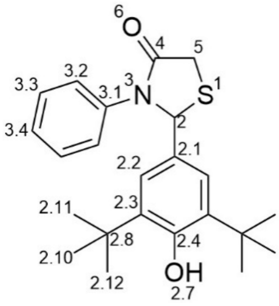

$C_{23}H_{29}NO_2S$, M.W.: 383.55 g/mol, CLogP: 6.37, light orange solid, yield: 0.2177 g (57%), m.p.: 141-143ºC.

TLC System: 7:3 Hexane:Ethyl Acetate. Rf: 0.70

^{1}H NMR (400 MHz, $CDCl_3$): δ (ppm, J_{H-H} = Hz): 7.29 – 7.24 (m, 2H, aryl), 7.18 – 7.13 (m, 1H, H3.4), 7.11 – 7.06 (m, 2H, aryl), 7.04 (s, 1H, H2.2, H2.6), 6.05 (s, 1H, H2), 5.21 (s, 1H, H2.7), 3.95 (dd, ^2J = 15.77, ^4J = 1.58 Hz, 1H, H5a), 3.86 (dd, ^2J = 15.77, ^4J = 0.86 Hz, 1H, H5b), 1.35 (s, 18H, H2.10-H2.15).

^{13}C NMR (101 MHz, $CDCl_3$) δ (ppm): 171.2 (C4), 154.3 (C2.4), 137.8 (C3.1), 136.2 (2C, C2.3, C2.5), 129.2 (C2.1), 129.1 (2C, aryl), 127.2 (C3.4), 126.4 (2C, aryl), 124.1 (2C, C2.2, C2.6), 66.6 (C2), 34.4 (2C, C2.8, C2.9), 33.8 (C5), 30.3 (6C, C2.10-C2.15).

GC: T_R = 14.3 minutes.

MS (70 eV): m/z (%) = 383 (M^+, 17), 261 (10), 260 (51), 249 (9), 207 (27), 104 (41), 77 (36), 57 (100), 41 (23).

HRMS (ESI) m/z: $[M + H]^+$ calculated exact mass (Trace Finder) for $C_{23}H_{29}NO_2S$ = 384.1991, found = 384.1990.

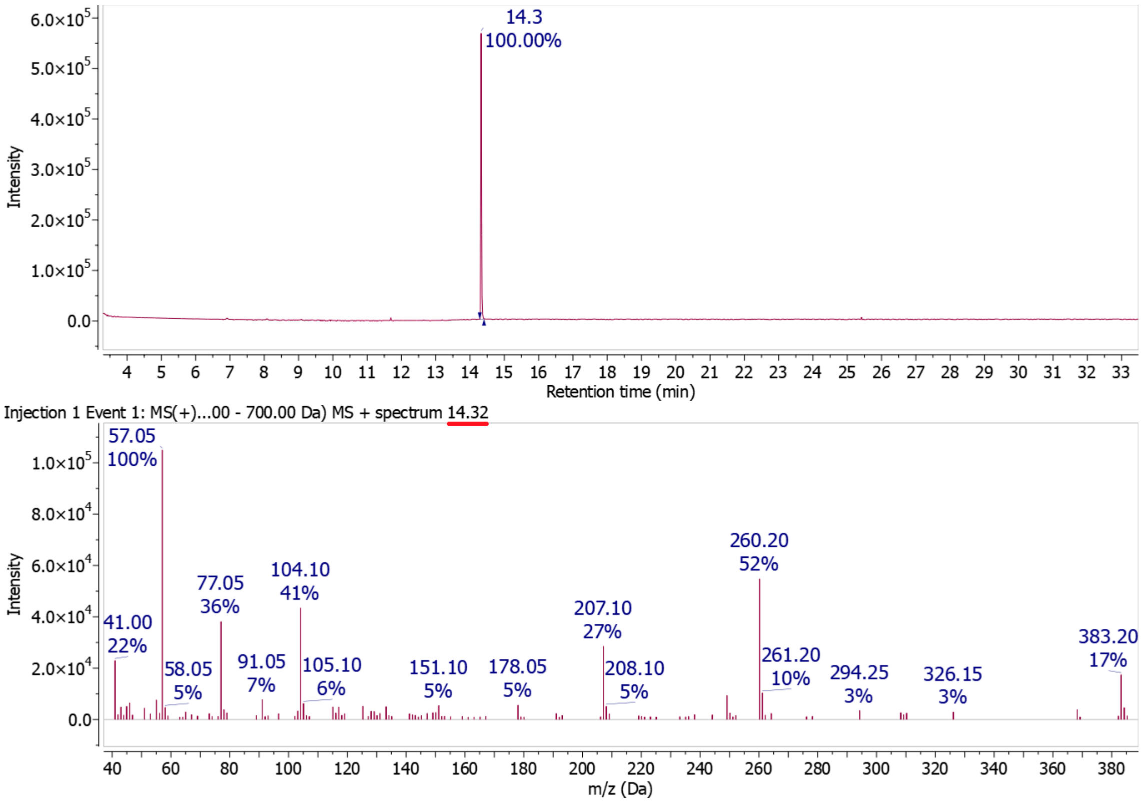

14.3
100.00%
Intensity
Retention time (min)
Injection 1 Event 1: MS(+)...00 - 700.00 Da) MS + spectrum 14.32
57.05
100%
41.00
22%
58.05
5%
77.05
36%
91.05
7%
104.10
41%
105.10
6%
151.10
5%
178.05
5%
207.10
27%
208.10
5%
260.20
52%
261.20
10%
294.25
3%
326.15
3%
383.20
17%
Intensity
m/z (Da)

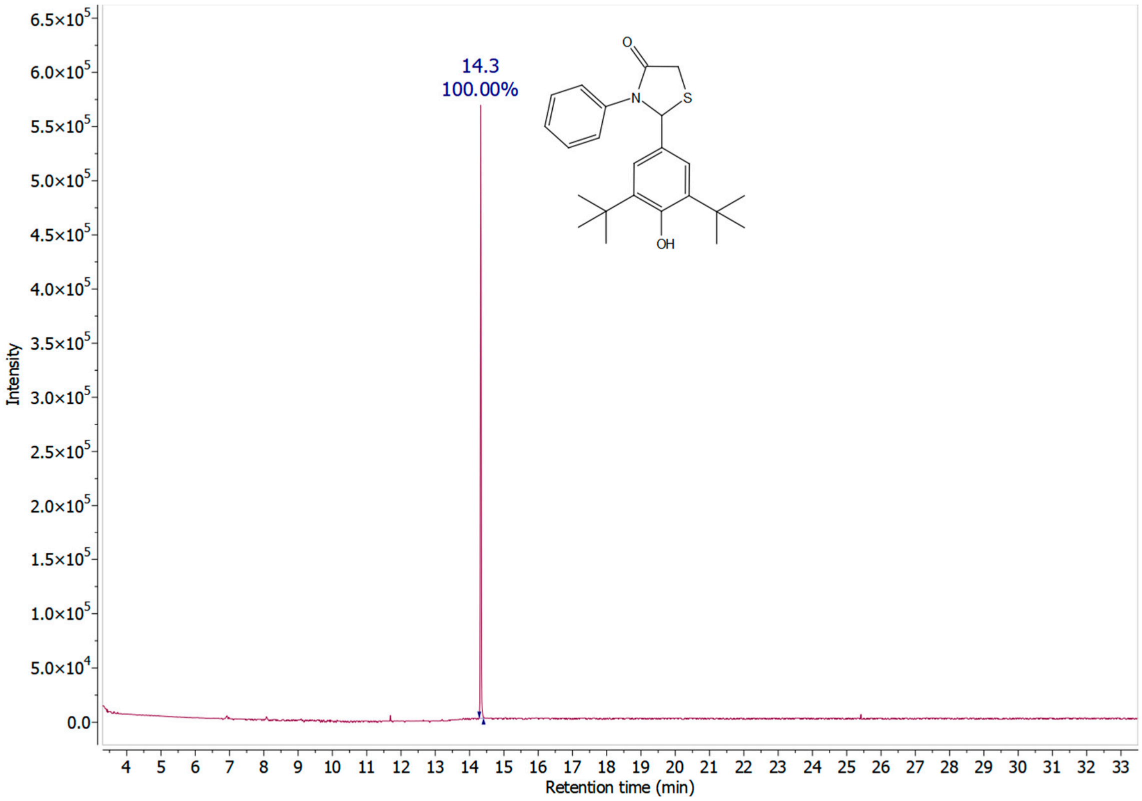

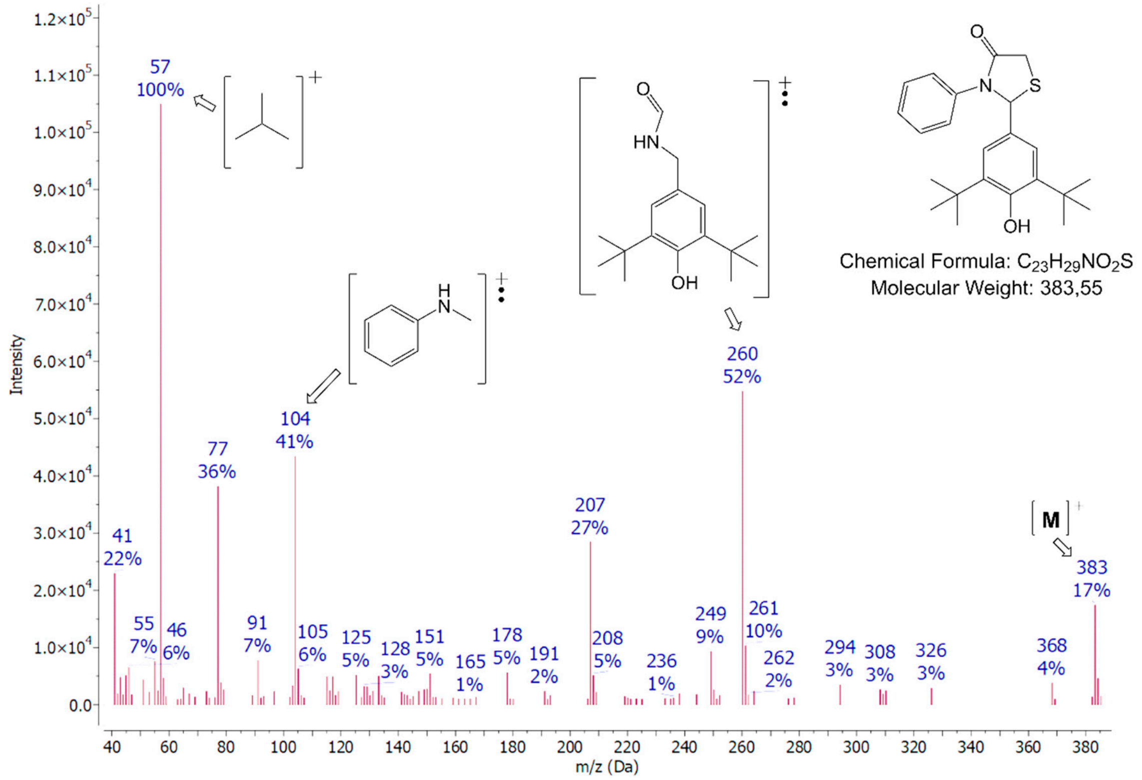

Figure S4. Chromatogram, mass spectra and fragmentation spectra of compound **4b**.

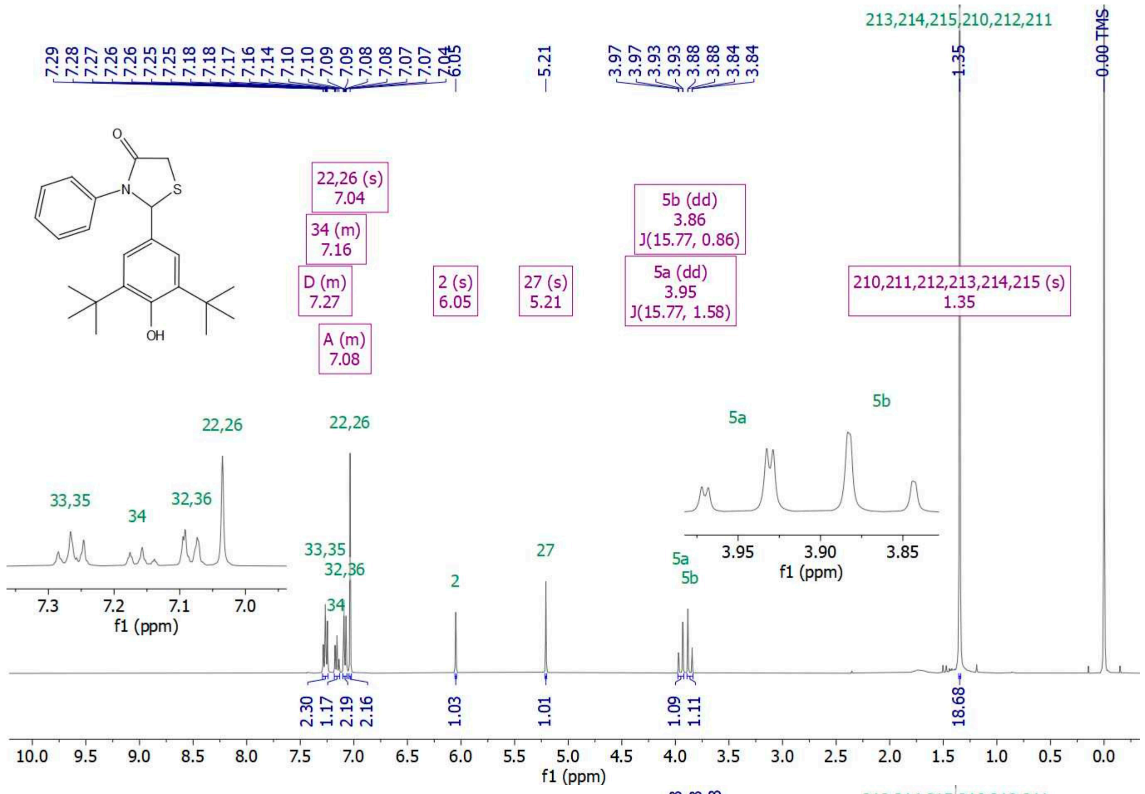

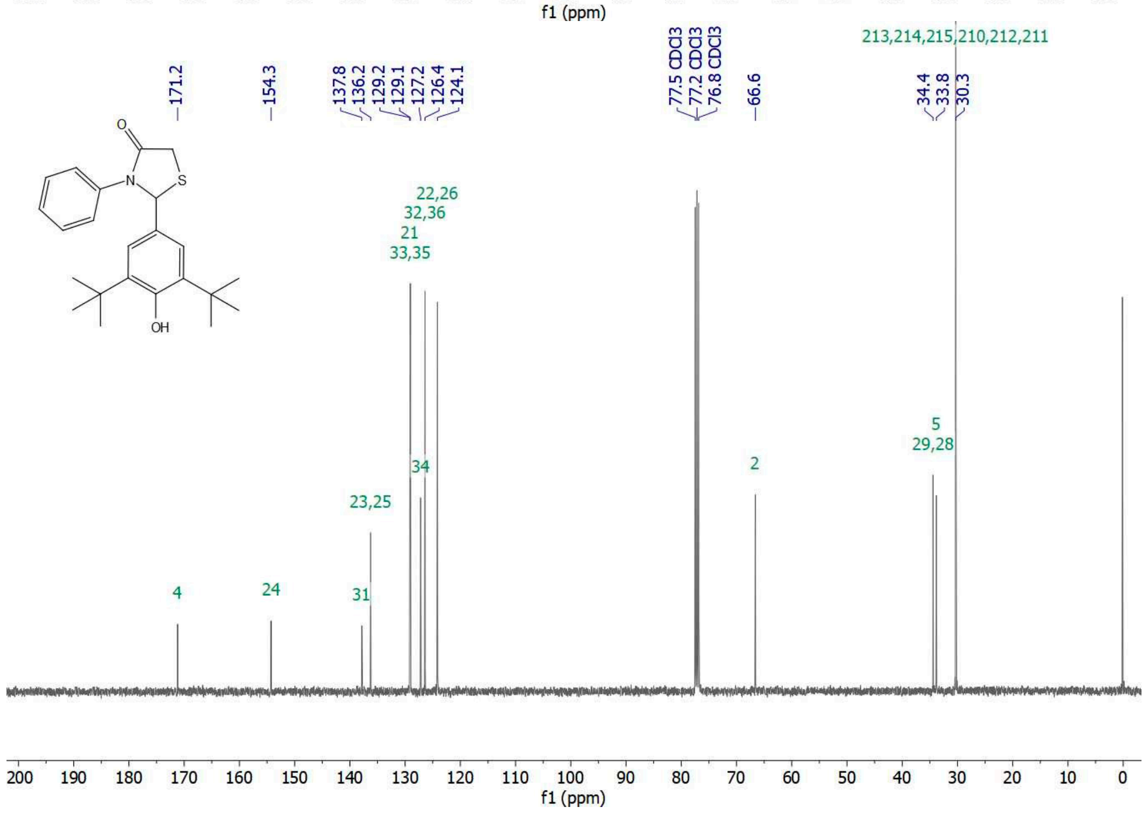

Figure S5. ^{1}H and ^{13}C NMR spectra of compound **4b**.

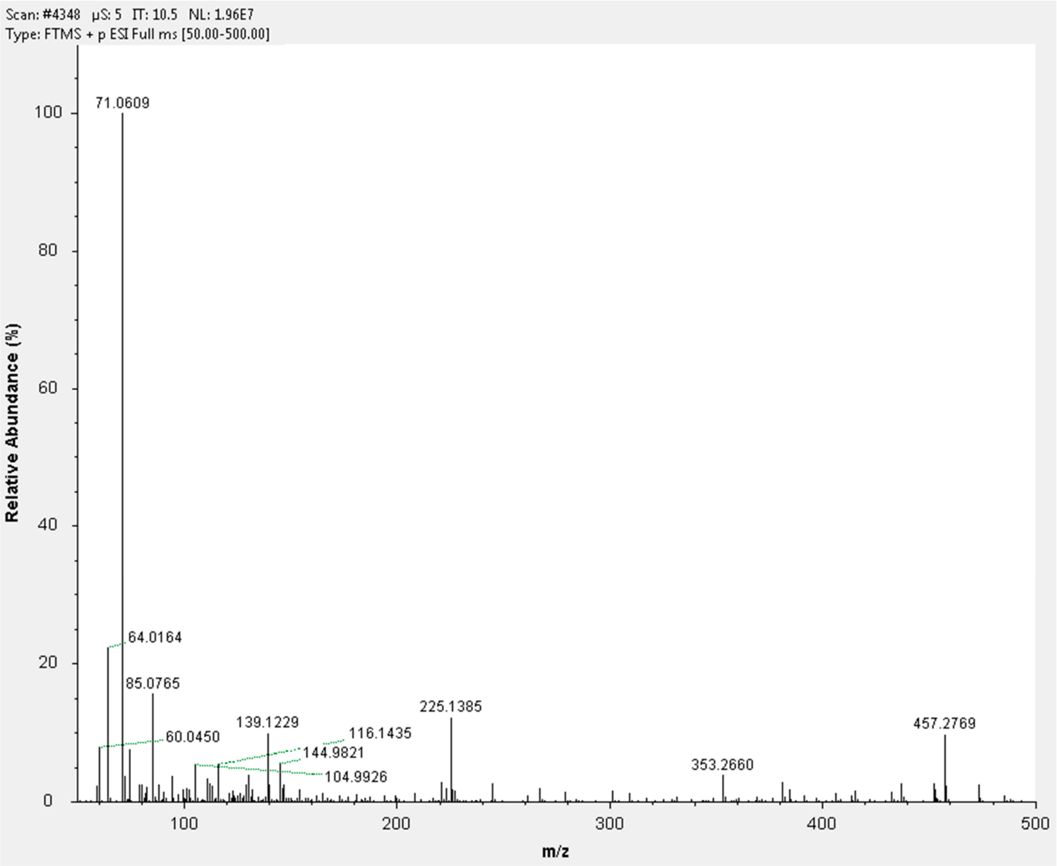

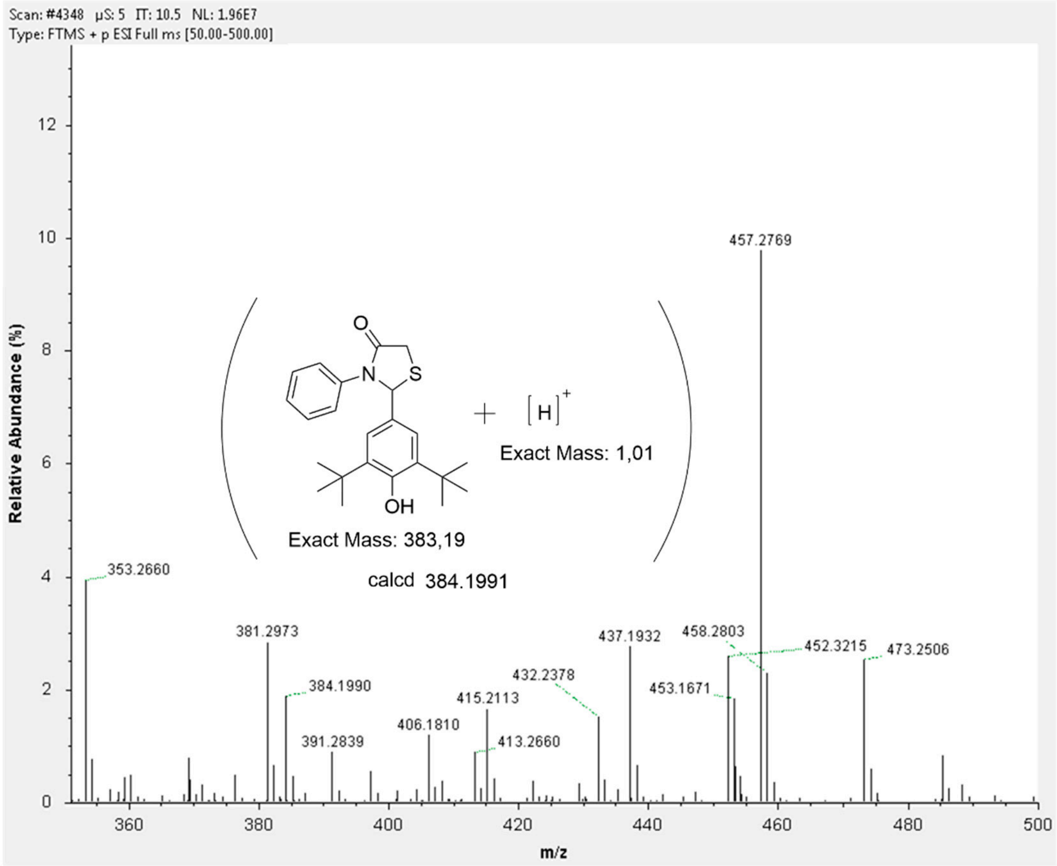

Figure S6. HRMS (ESI$^+$) of compound **4b** showing the $[M + H]^+$ ion and adducts from fragmentation.

1.3. 3-(Benzo[d][1,3]dioxol-5-yl)-2-(3,5-di-tert-butyl-4-hydroxyphenyl)thiazolidin-4-one **4c**

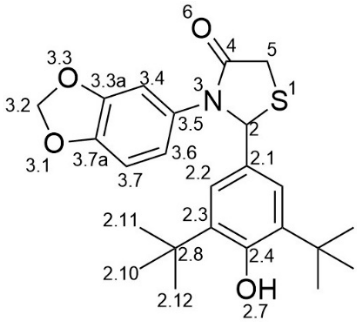

$C_{24}H_{29}NO_4S$, M.W.: 427.56 g/mol, CLogP: 6.33, dark red solid, yield: 0.3432 g (80%), m.p.: 135-137ºC.

TLC System: 7:3 Hexane:Ethyl Acetate. Rf: 0.59

^{1}H NMR (400 MHz, $CDCl_3$): δ (ppm, J_{H-H} = Hz): 7.04 (s, 2H, H2.2, H2.6), 6.68 (d, ^3J = 8.14, 1H, aryl), 6.53 (d, ^4J = 2.04, 1H, aryl), 6.50 (dd, ^3J = 8.19, ^4J = 2.07, 1H, aryl), 5.91 – 5.89 (m, 3H, H2, H3.2), 5.25 (s, 1H, H2.7), 3.93 (dd, ^2J = 15.77, ^4J = 1.69, 1H, H5a), 3.84 (d, ^2J = 15.77, 1H, H5b), 1.37 (s, 18H, H2.10-H2.15).

^{13}C NMR (101 MHz, $CDCl_3$) δ (ppm): 171.3 (C4), 154.4 (C2.4), 148.0 (1C, aryl), 146.8 (1C, aryl), 136.3 (2C, C2.3, C2.5), 131.5 (1C, aryl), 129.1 (C2.1), 124.3 (2C, C2.2, C2.6), 120.4 (1C, aryl), 108.3 (1C, aryl), 108.2 (1C, aryl), 101.6 (C3.2), 66.9 (C2), 34.4 (2C, C2.8, C2.9), 33.6 (C5), 30.3 (6C, C2.10-C2.15).

GC: T_R = 17.5 minutes.

MS (70 eV): m/z (%) = 427 (M^+, 26), 353 (4), 261 (18), 260 (100), 207 (10), 148 (8), 57 (31), 41 (5).

HRMS (ESI) m/z: $[M + H]^+$ calculated exact mass (Trace Finder) for $C_{24}H_{29}NO_4S$ = 428.1890, found = 428.1890.

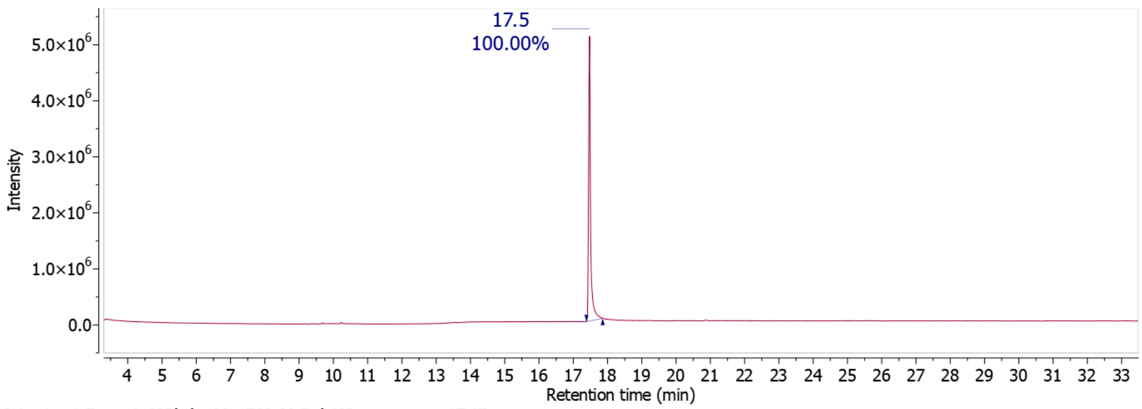

Injection 1 Event 1: MS(+)...00 - 700.00 Da) MS + spectrum 17.47

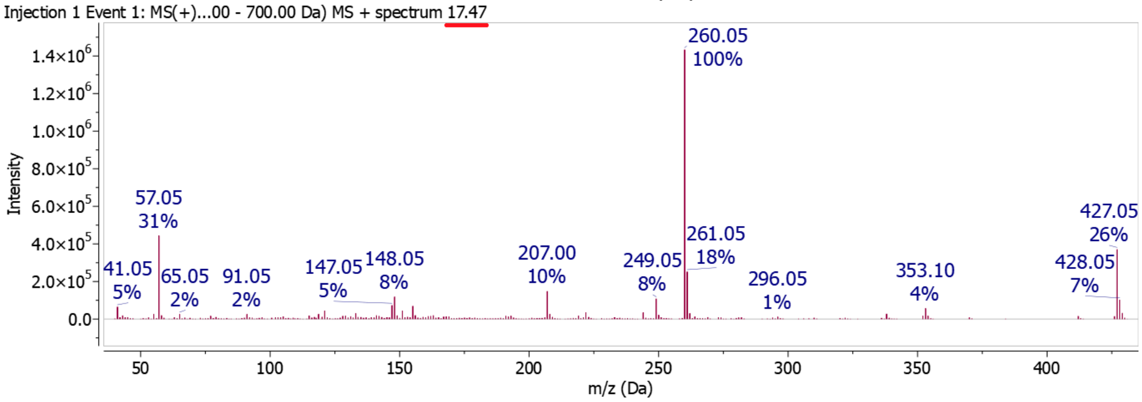

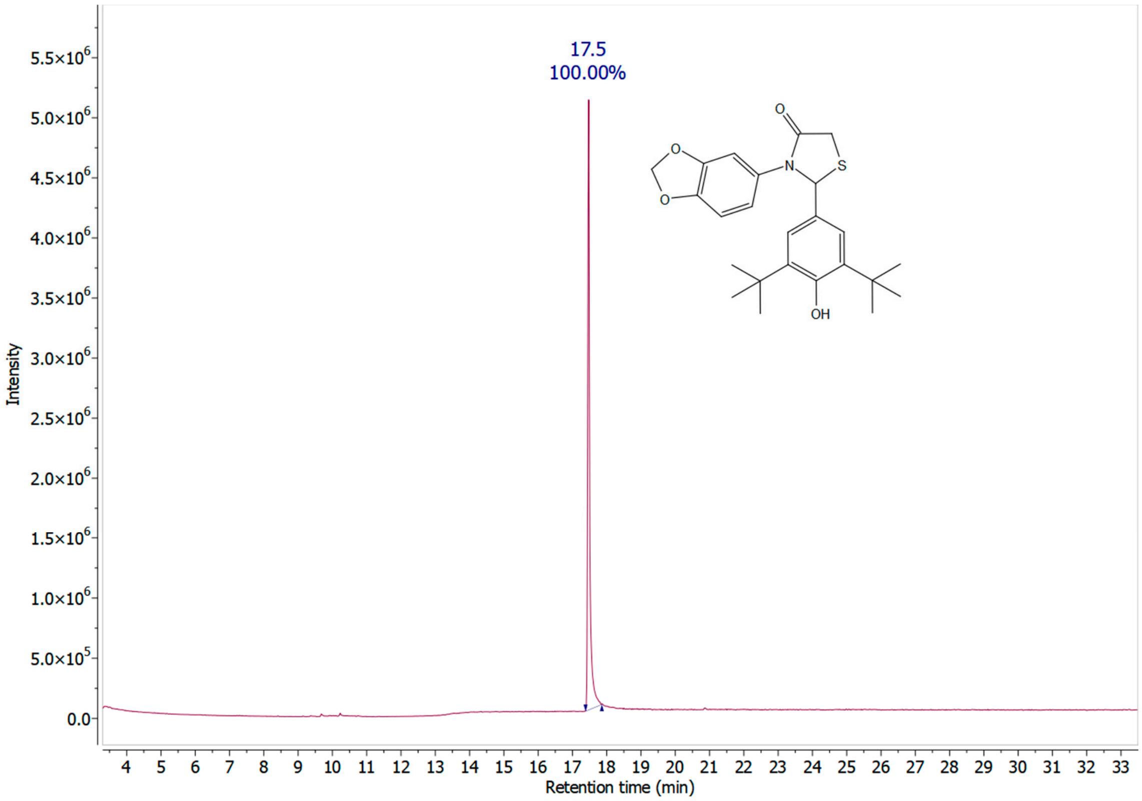

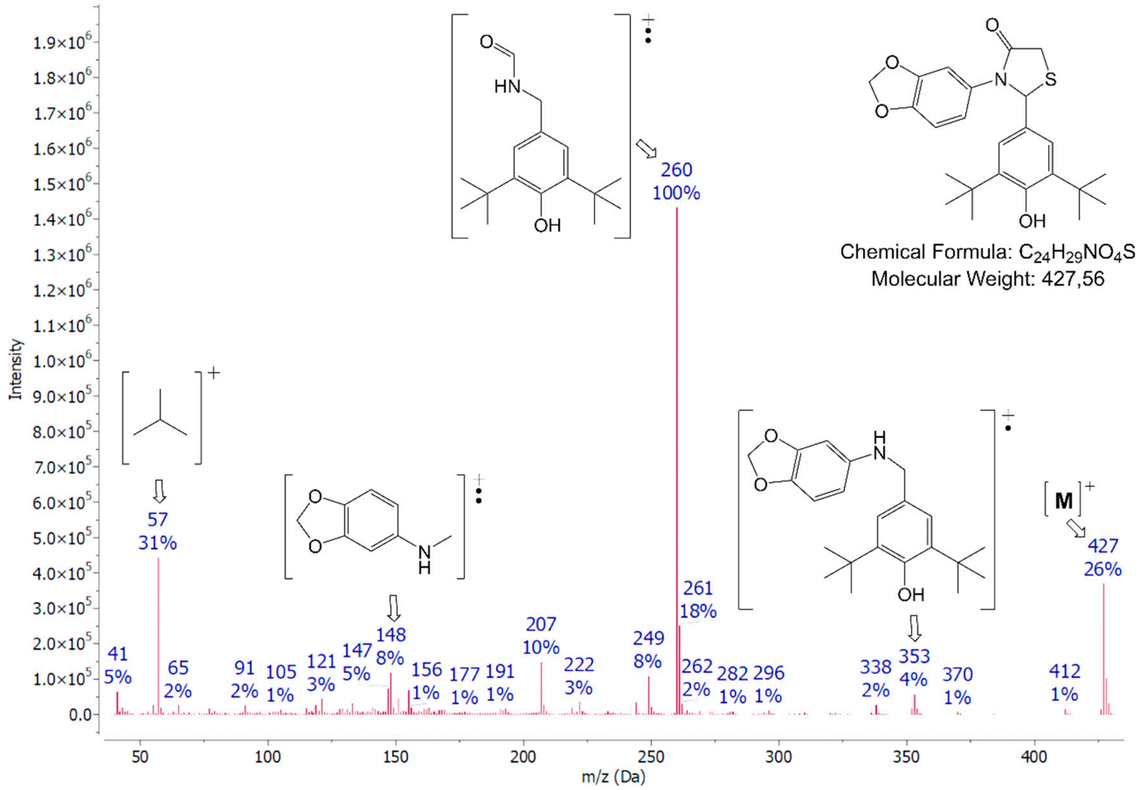

Figure S7. Chromatogram, mass spectra and fragmentation spectra of compound **4c**.

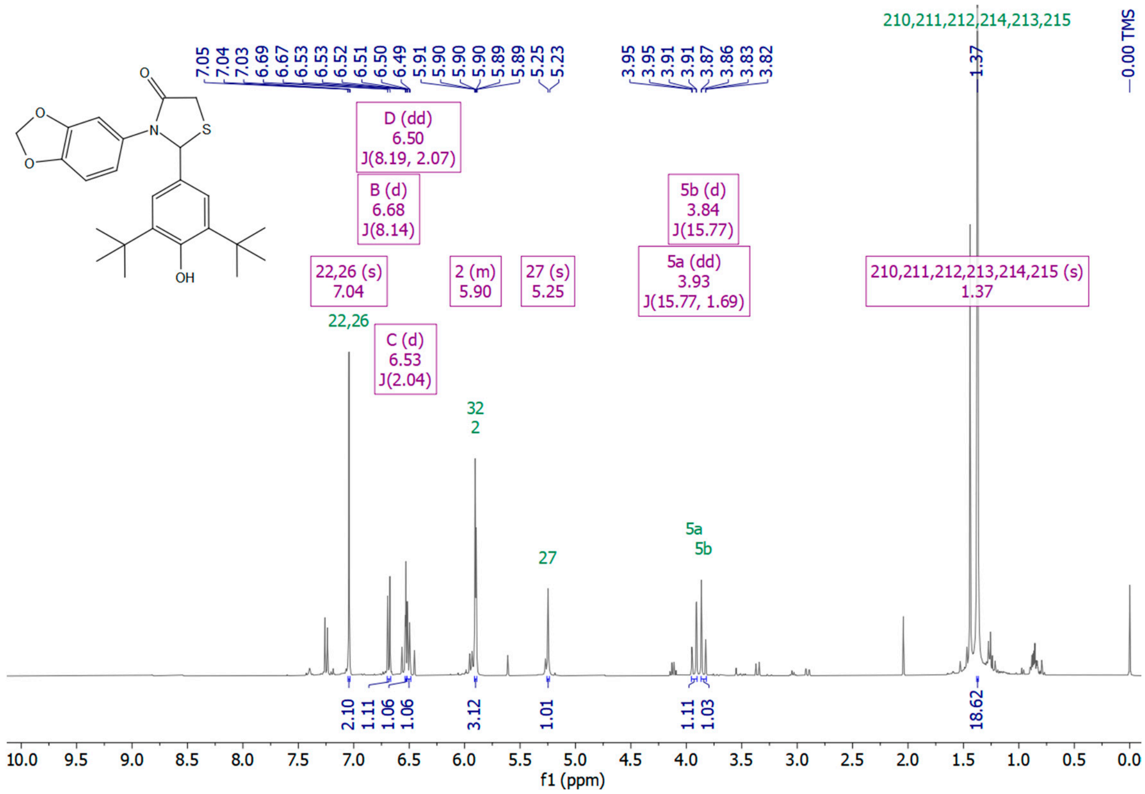

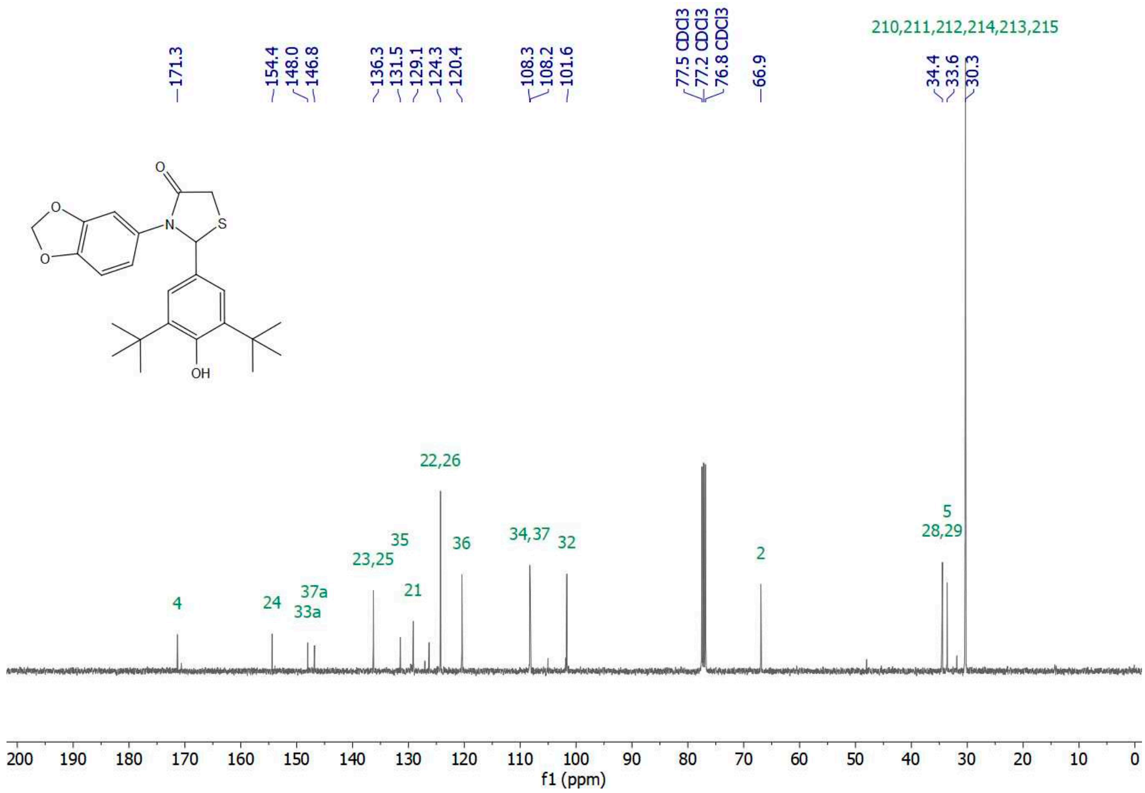

Figure S8. ^{1}H and ^{13}C NMR spectra of compound **4c**.

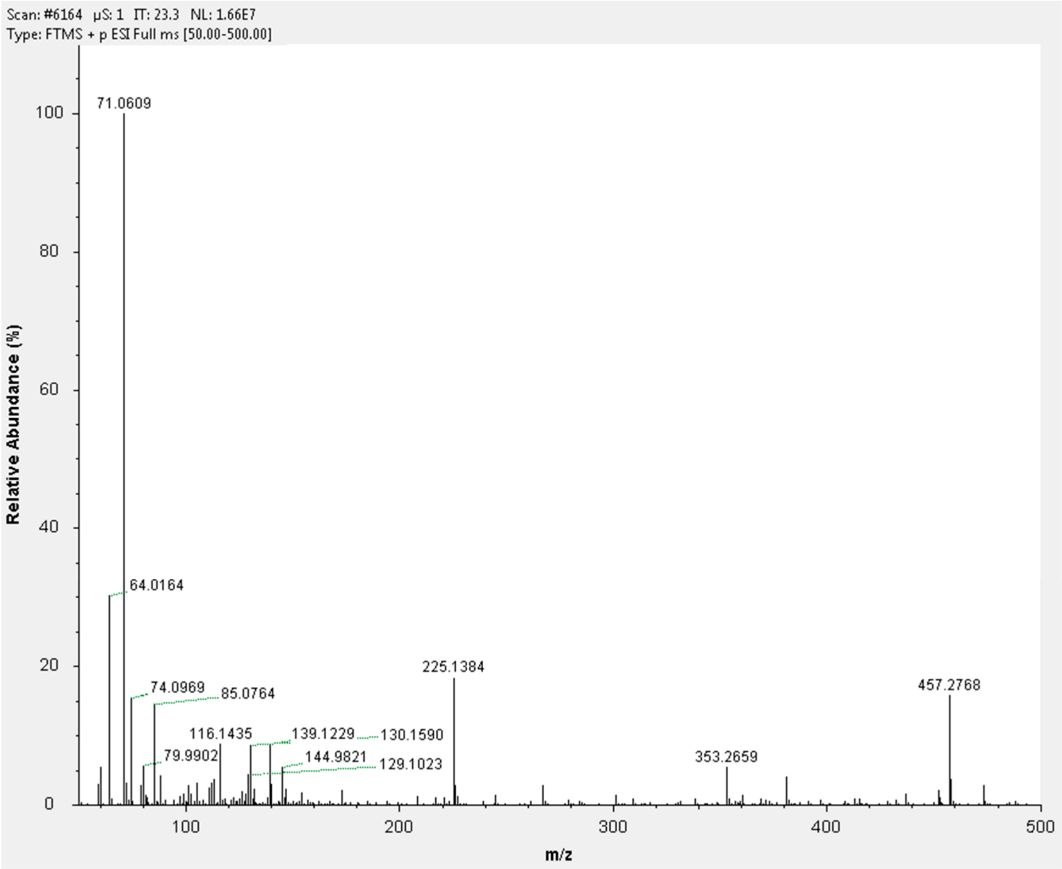

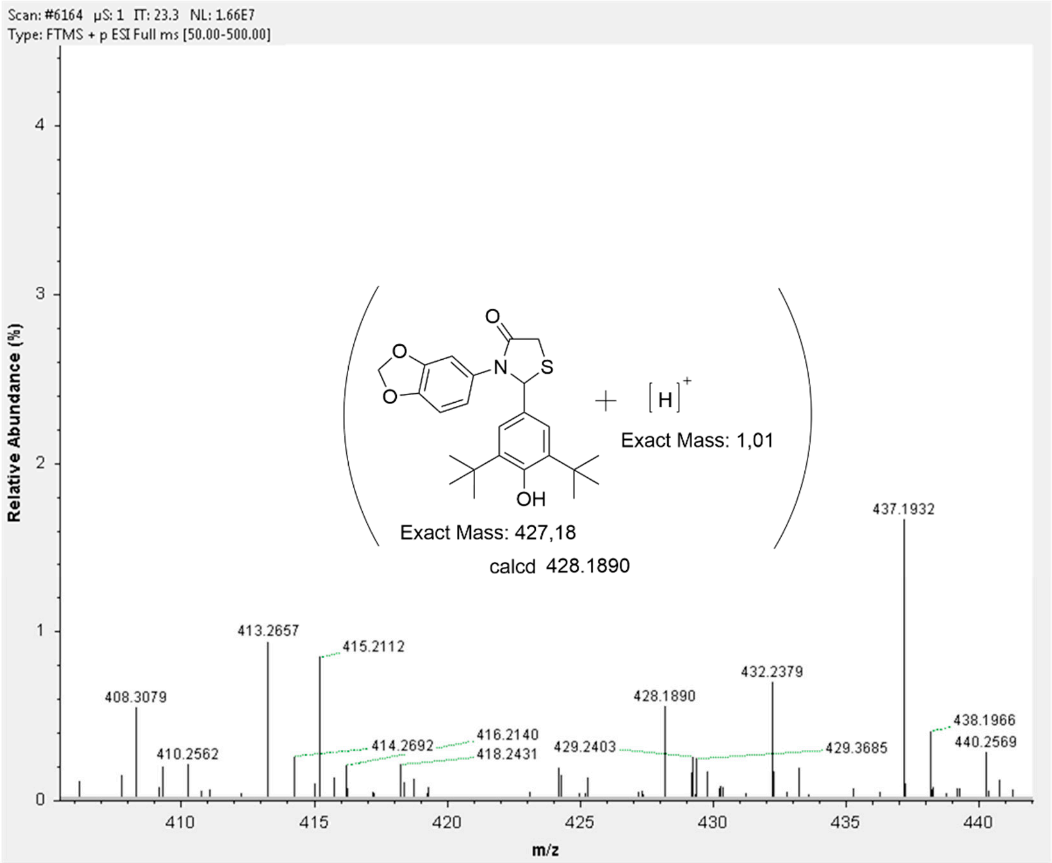

Figure S9. HRMS (ESI+) of compound **4c** showing the [M + H]+ ion and adducts from fragmentation.

1.4. 2-(3,5-di-tert-butyl-4-hydroxyphenyl)-3-(1H-1,2,4-triazol-3-yl)thiazolidin-4-one **4d**

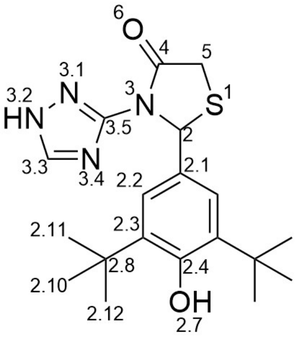

$C_{19}H_{26}N_4O_2S$, M.W.: 374.50 g/mol, CLogP: 3.94, white solid, yield: 0.1391 g (37%), m.p.: 229-231ºC.

TLC System: 7:3 Hexane:Ethyl Acetate. Rf: 0.20

^{1}H NMR (400 MHz, $CDCl_3$): δ (ppm, J_{H-H} = Hz): 7.77 (s, 1H, H3.3), 7.13 (s, 2H, H2.2, H2.6), 6.48 (s, 1H, H2), 5.27 (s, 1H, H2.7), 4.05 (d, ^2J = 16.59, 1H, H5a), 3.77 (d, ^2J = 16.54, 1H, H5b), 1.39 (s, 18H, H2.10-H2.15).

^{13}C NMR (101 MHz, $CDCl_3$) δ (ppm): 172.4 (C4), 154.3 (C2.4), 149.2 (1C, triazolyl), 148.8 (1C, triazolyl), 136.4 (2C, C2.3, C2.5), 130.0 (C2.1), 122.8 (2C, C2.2, C2.6), 63.0 (C2), 34.5 (2C, C2.8, C2.9), 33.0 (C5), 30.3 (6C, C2.10-C2.15).

GC: T_R = 14.8 minutes.

MS (70 eV): m/z (%) = 375 (M^++1, 14), 374 (M^+, 64), 332 (59), 306 (22), 249 (44), 207 (16), 169 (100), 141 (16), 115 (66), 95 (27), 57 (79), 41 (28).

HRMS (ESI) m/z: $[M + H]^+$ calculated exact mass (Trace Finder) for $C_{19}H_{26}N_4O_2S$ = 375.1849, found = 375.1847.

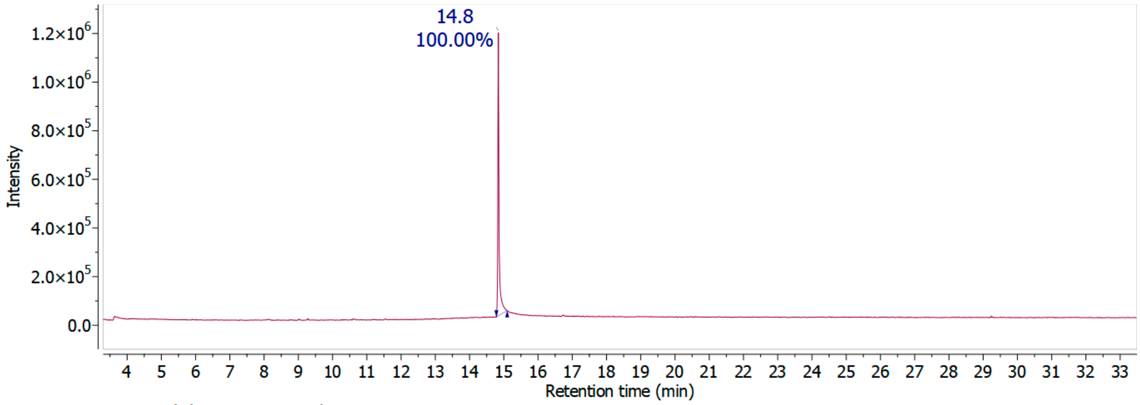

Injection 1 Event 1: MS(+)...00 - 700.00 Da) MS + spectrum 14.84

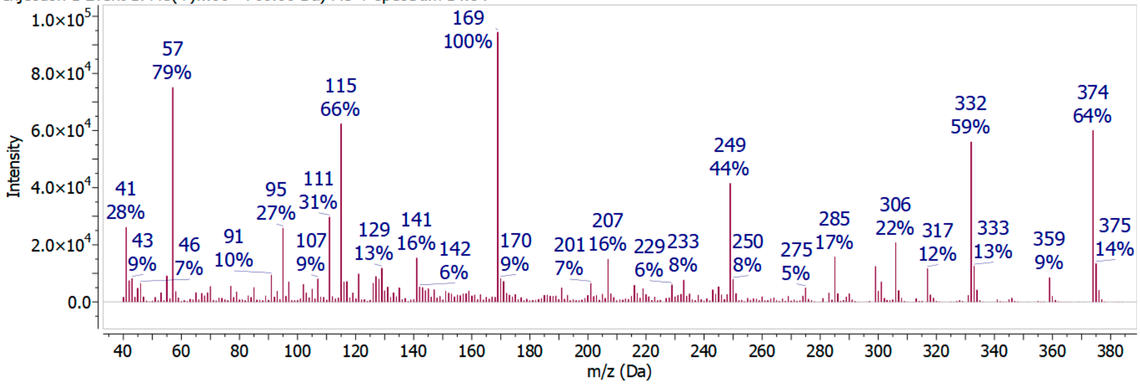

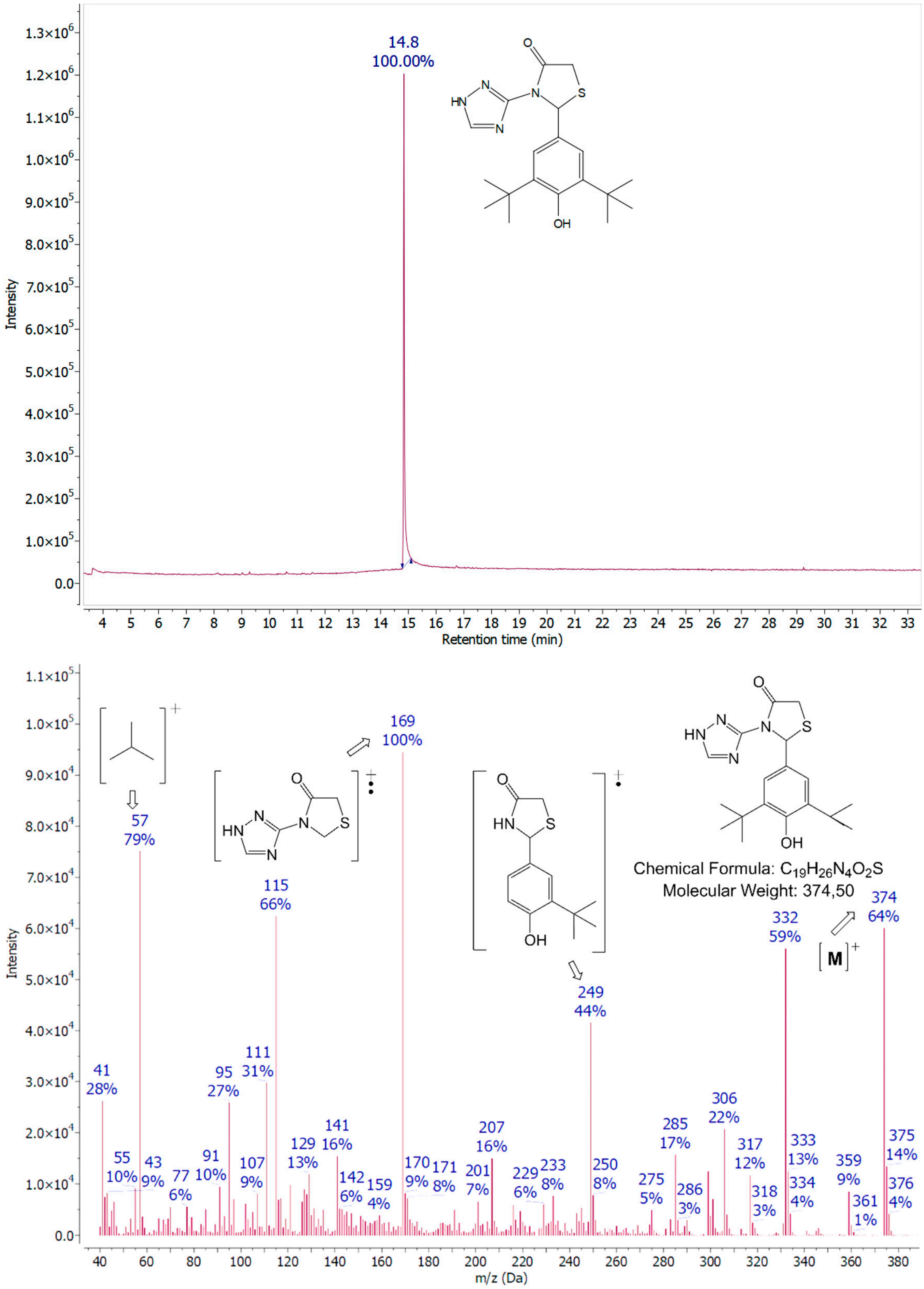

Figure S10. Chromatogram, mass spectra and fragmentation spectra of compound **4d**.

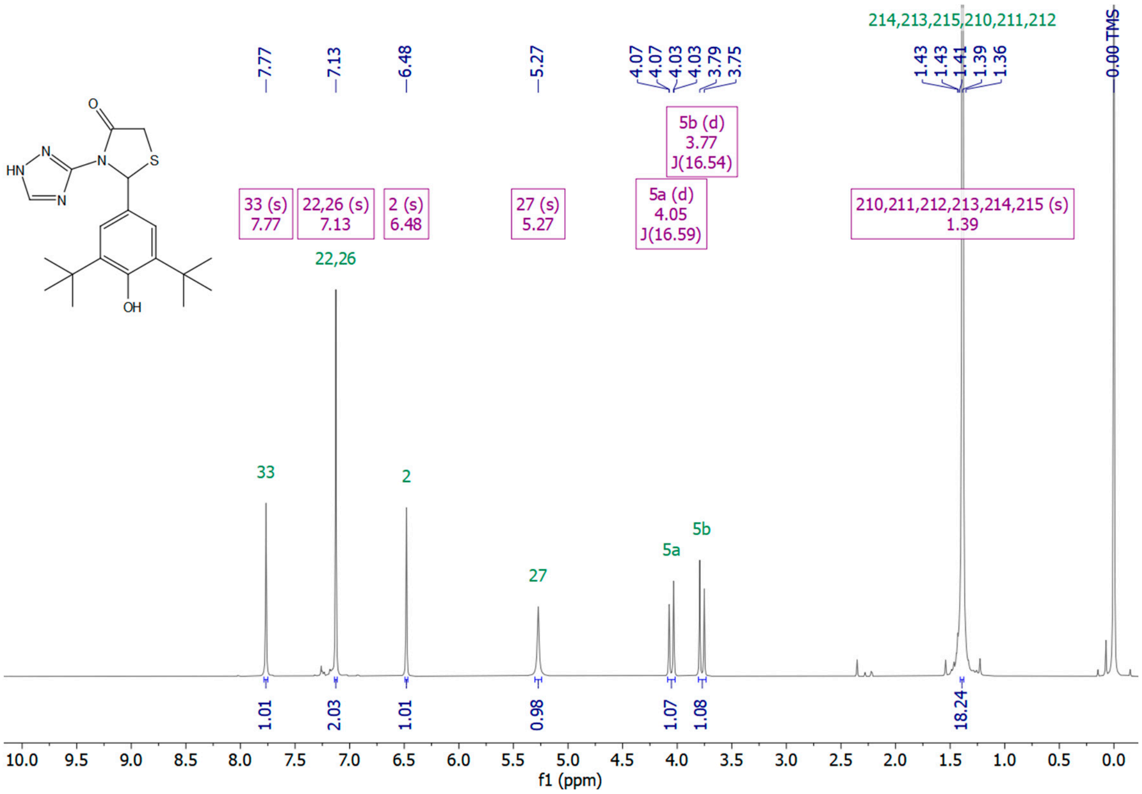

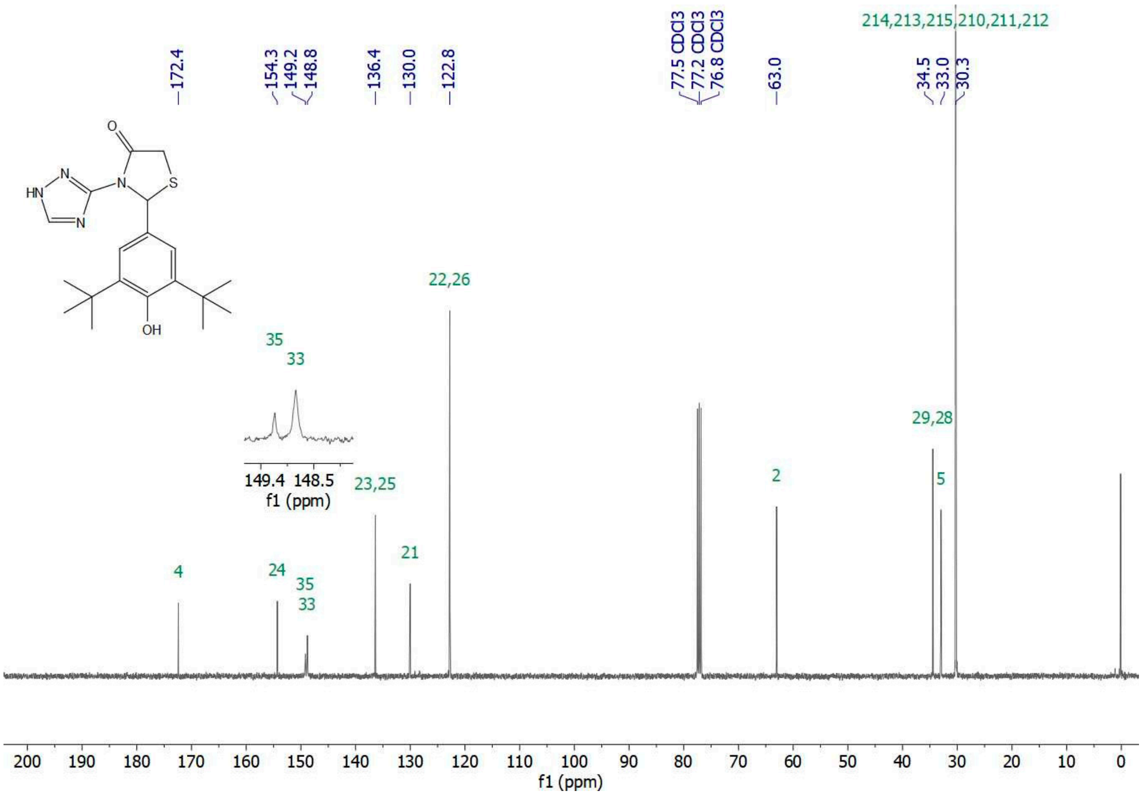

Figure S11. ^{1}H and ^{13}C NMR spectra of compound **4d**.

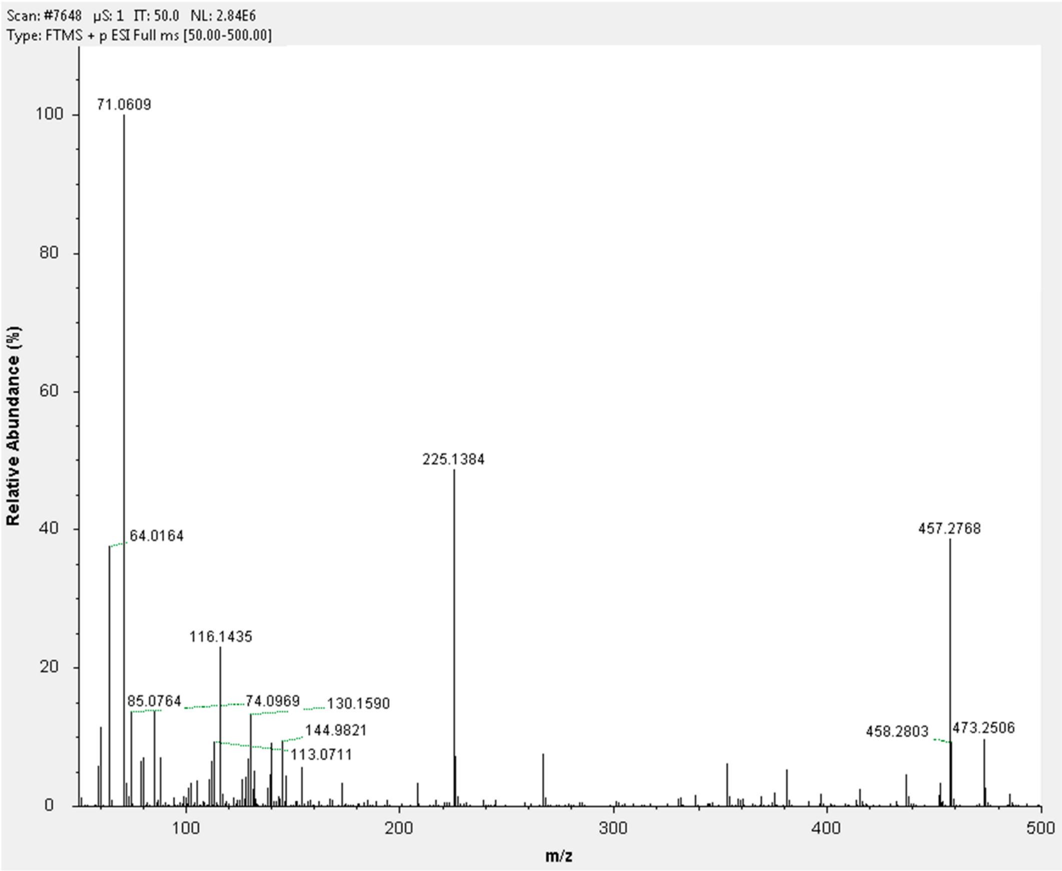

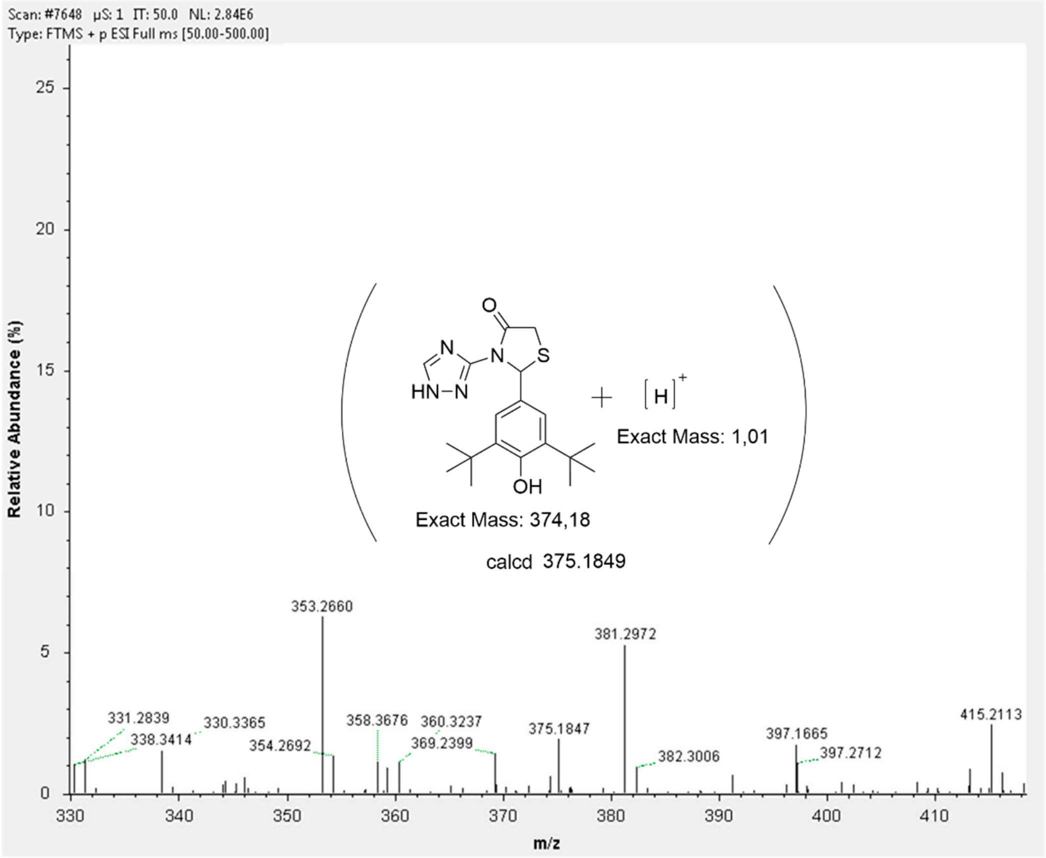

Figure S12. HRMS (ESI+) of compound **4d** showing the [M + H]+ ion and adducts from fragmentation.

1.5. 2-(3,5-di-tert-butyl-4-hydroxyphenyl)-3-(furan-2-ylmethyl)thiazolidin-4-one **4e**

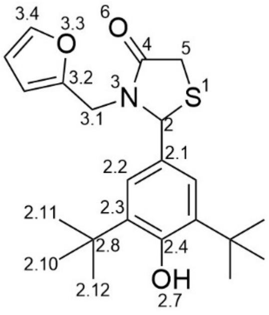

$C_{22}H_{29}NO_3S$, M.W.: 387.54 g/mol, CLogP: 5.06, light orange solid, yield: 0.1421 g (37%), m.p.: 112-114ºC.

TLC System: 7:3 Hexane:Ethyl Acetate. Rf: 0.85

^1H NMR (400 MHz, $CDCl_3$): δ (ppm, $J_{H\text{-}H}$ = Hz): 7.34 (s, 1H, furyl), 7.10 (s, 2H, H2.2, H2.6), 6.28 (dd, ^4J = 3.21, ^4J = 1.86 Hz, 1H, furyl), 6.06 (d, ^4J = 3.17 Hz, 1H), 5.49 (s, 1H, H2), 5.35 (s, 1H, H2.7), 4.89 (d, ^2J = 15.35 Hz, 1H, H3.1a), 3.82 (dd, ^2J = 15.63, ^4J = 1.97 Hz, 1H, H5a), 3.72 (d, ^2J = 15.67 Hz, 1H, H5b), 3.71 (d, ^2J = 15.27 Hz, 1H, H3.1b), 1.43 (s, 18H, H2.10-H2.15).

^{13}C NMR (101 MHz, $CDCl_3$) δ (ppm): 171.1 (C4), 154.7 (C2.4), 149.5 (1C, furyl), 142.7 (1C, furyl), 136.5 (2C, C2.3, C2.5), 128.5 (C2.1), 124.7 (2C, C2.2, C2.6), 110.4 (1C, furyl), 109.1 (1C, furyl), 64.2 (C2), 39.1 (C3.1), 34.5 (2C, C2.8, C2.9), 33.3 (C5), 30.3 (6C, C2.10-C2.15).

GC: T_R = 13.8 minutes.

MS (70 eV): m/z (%) = 388 (M^++1, 19), 387 (M^+, 68), 313 (22), 312 (75), 250 (10), 251 (45), 235 (14), 138 (12), 137 (77), 109 (14), 96 (11), 81 (100), 57 (34), 41 (14).

HRMS (ESI) m/z: $[M + H]^+$ calculated exact mass (Trace Finder) for $C_{22}H_{29}NO_3S$ = 388.1940, found = 388.1939.

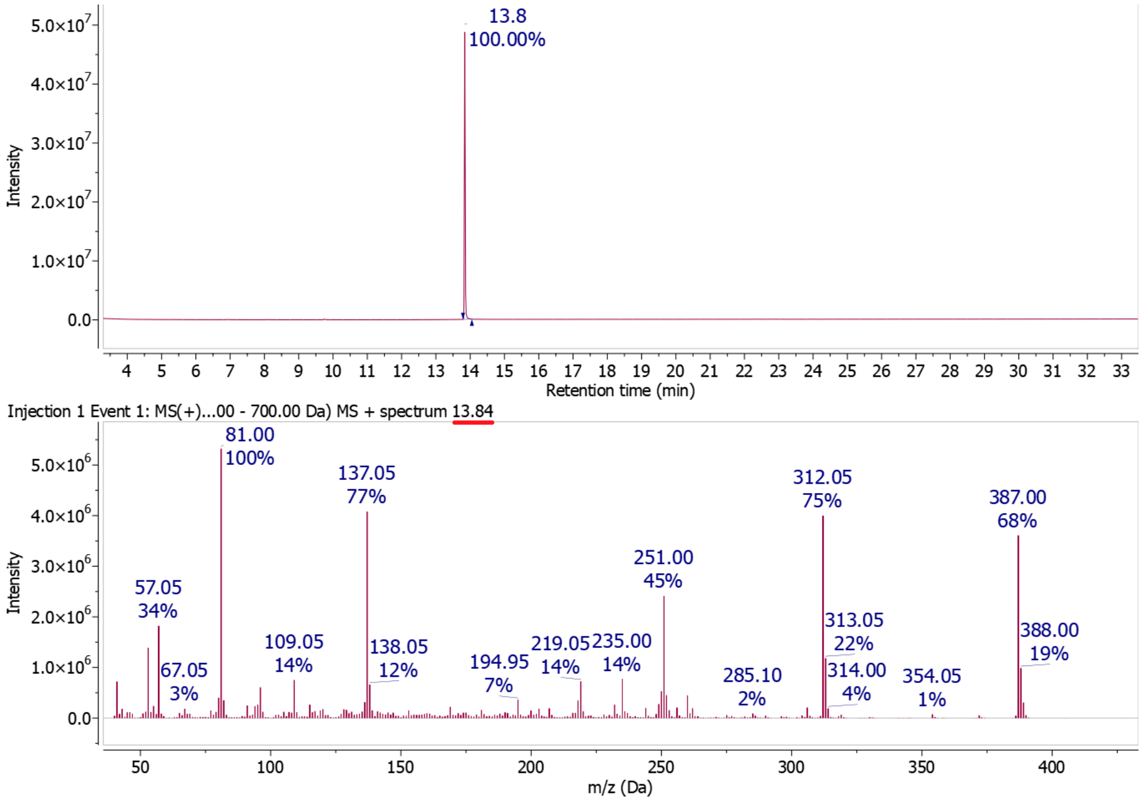
13.8
100.00%
Intensity
5.0×10^7
4.0×10^7
3.0×10^7
2.0×10^7
1.0×10^7
0.0
Retention time (min)
Injection 1 Event 1: MS(+)...00 - 700.00 Da) MS + spectrum 13.84
81.00
100%
137.05
77%
312.05
75%
387.00
68%
251.00
45%
57.05
34%
313.05
22%
388.00
19%
109.05
14%
138.05
12%
67.05
3%
194.95
7%
219.05
14%
235.00
14%
285.10
2%
314.00
4%
354.05
1%
5.0×10^6
4.0×10^6
3.0×10^6
2.0×10^6
1.0×10^6
m/z (Da)

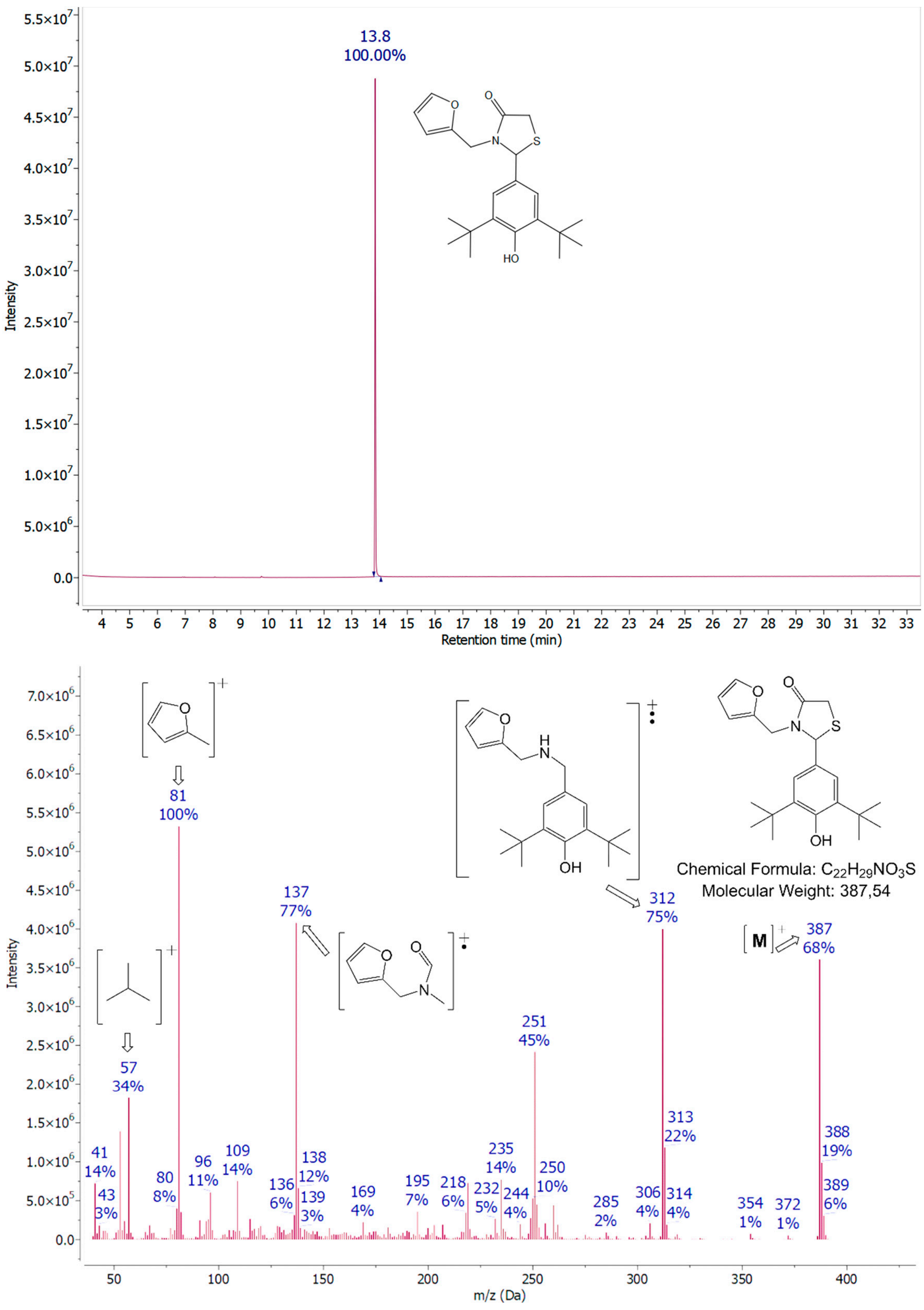

Figure S13. Chromatogram, mass spectra and fragmentation spectra of compound **4e**.

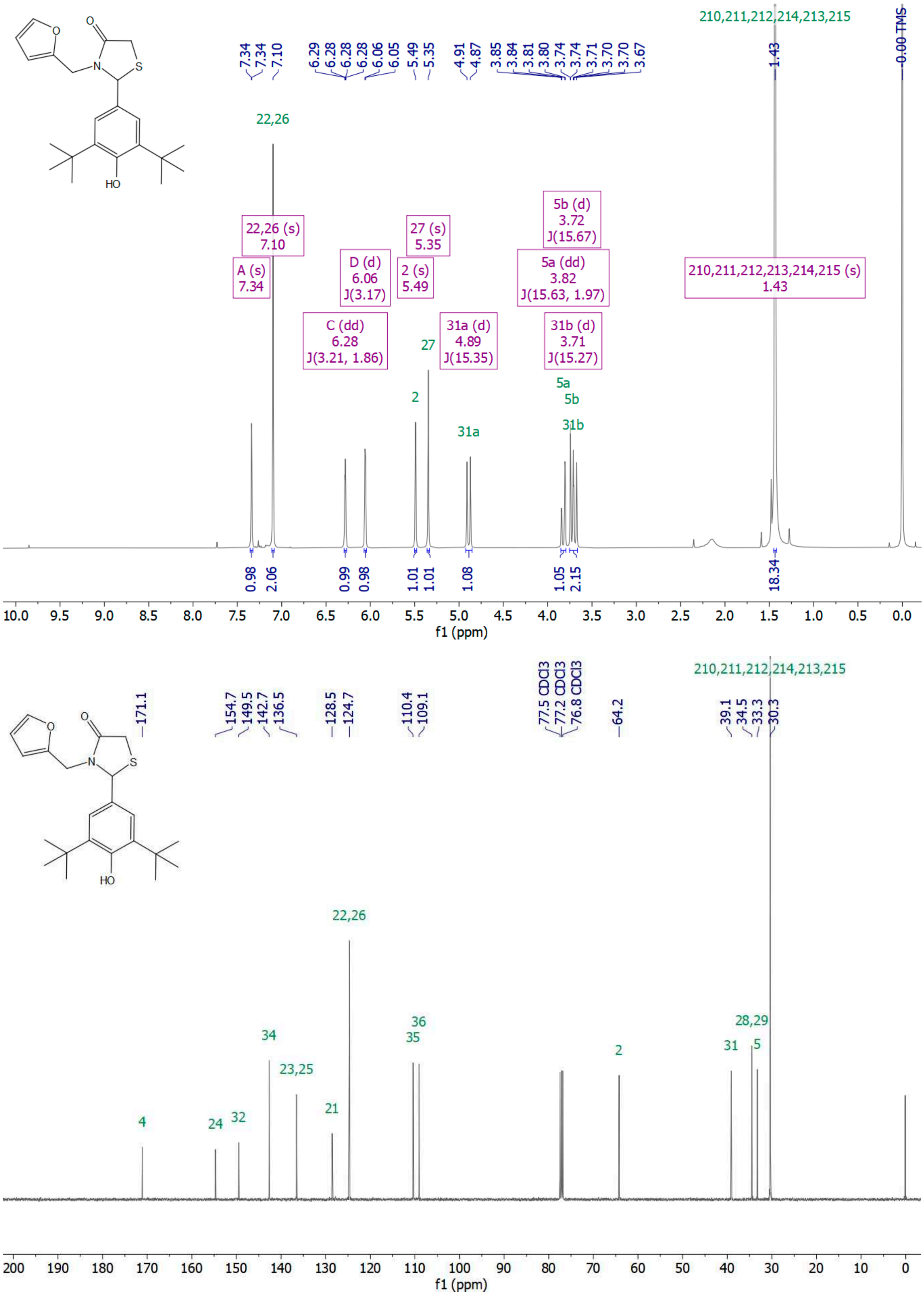

Figure S14. ^{1}H and ^{13}C NMR spectra of compound **4e**.

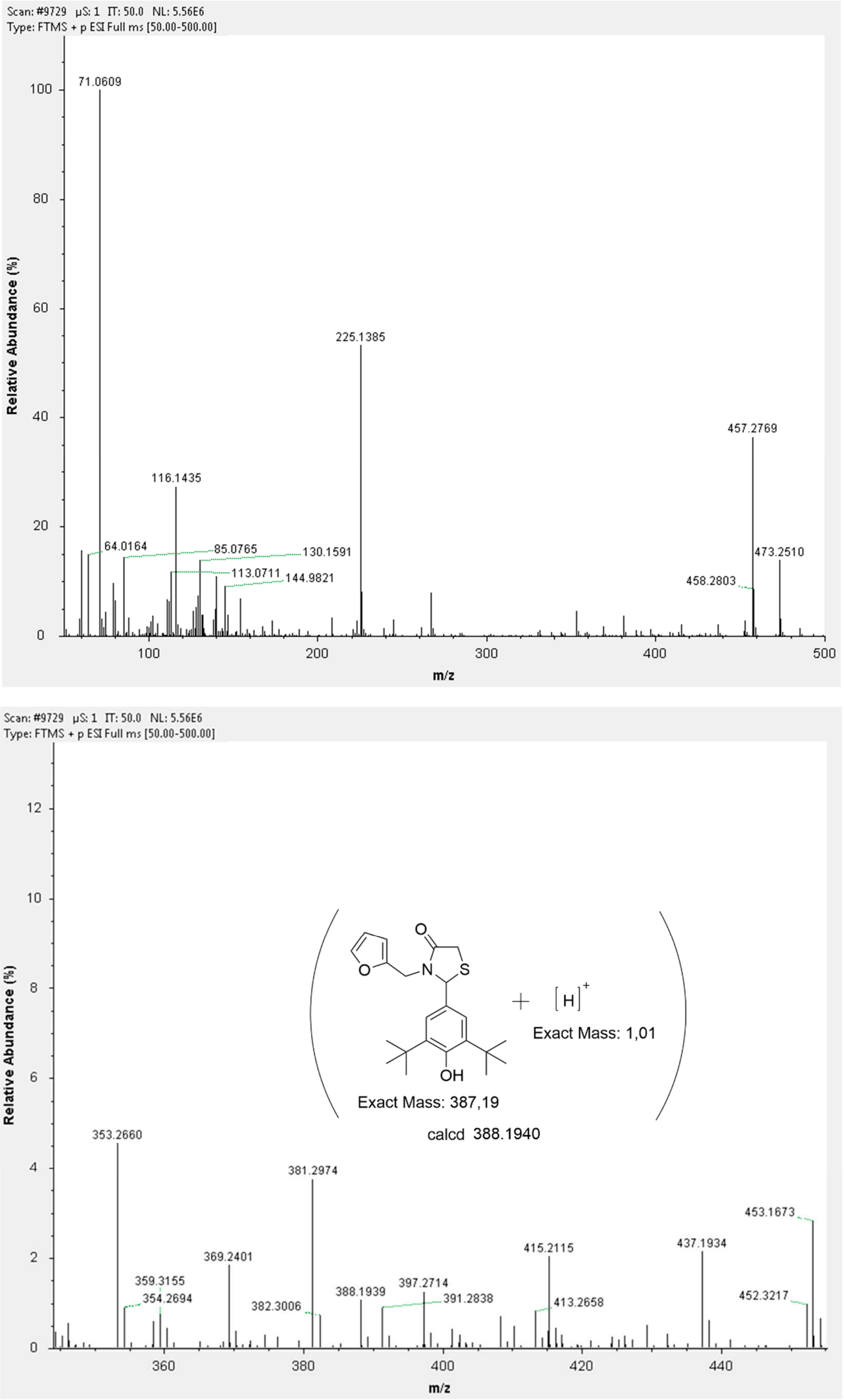

Figure S15. HRMS (ESI$^+$) of compound **4e** showing the $[M + H]^+$ ion and adducts from fragmentation.

1.6. 3-(1H-benzo[d]imidazol-2-yl)-2-(3,5-di-tert-butyl-4-hydroxyphenyl)thiazolidin-4-one **4f**

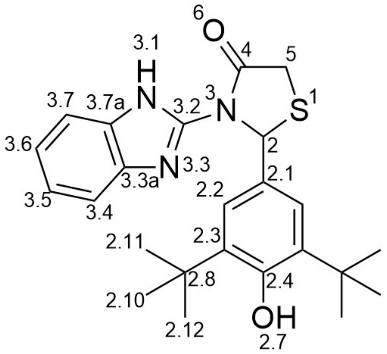

$C_{24}H_{29}N_3O_2S$, M.W.: 423.58 g/mol, CLogP: 5.79, light yellow flakes, yield: 0.1245 g (29%), m.p.: 216-218ºC.

TLC System: 7:3 Hexane:Ethyl Acetate. Rf: 0.72

^{1}H NMR (400 MHz, $CDCl_3$): δ (ppm, J_{H-H} = Hz): 11.04 (s, 1H, H3.1, NH), 7.62 – 7.55 (m, 1H, H3.5), 7.43 – 7.36 (m, 1H, H3.6), 7.26 (s, 2H, H2.2, H2.6), 7.25 – 7.17 (m, 1H, H3.7), 7.20 – 7.18 (m, 1H, H3.4), 6.76 (s, 1H, H2), 5.23 (s, 1H, H2.7), 4.12 (d, ^2J = 16.45, 1H, H5a), 3.80 (d, ^2J = 16.49, 1H, H5b), 1.38 (s, 18H, H2.10-H2.15).

^{13}C NMR (101 MHz, $CDCl_3$) δ (ppm): 172.8 (C4), 154.2 (C2.4), 145.3 (C3.2), 140.4 (C3.7a), 136.1 (2C, C2.3, C2.5), 131.6 (C3.3a), 130.6 (C2.1), 123.2 (2C, C2.2, C2.6), 122.6 (C3.7), 122.5 (C3.4), 118.8 (C3.5), 110.8 (C3.6), 63.2 (C2), 34.5 (2C, C2.8, C2.9), 33.7 (C5), 30.3 (6C, C2.10-C2.15).

GC: T_R = 17.0 minutes.

MS (70 eV): m/z (%) = 424 (M^{+1}, 25), 423 (M^+, 91), 382 (20), 381 (74), 377 (18), 348 (12), 249 (38), 218 (14), 207 (26), 176 (34), 164 (100), 160 (36), 146 (22), 133 (16), 119 (13), 57 (53), 41 (15).

HRMS (ESI) m/z: $[M + H]^+$ calculated exact mass (Trace Finder) for $C_{24}H_{29}N_3O_2S$ = 424.2053, found = 424.2052.

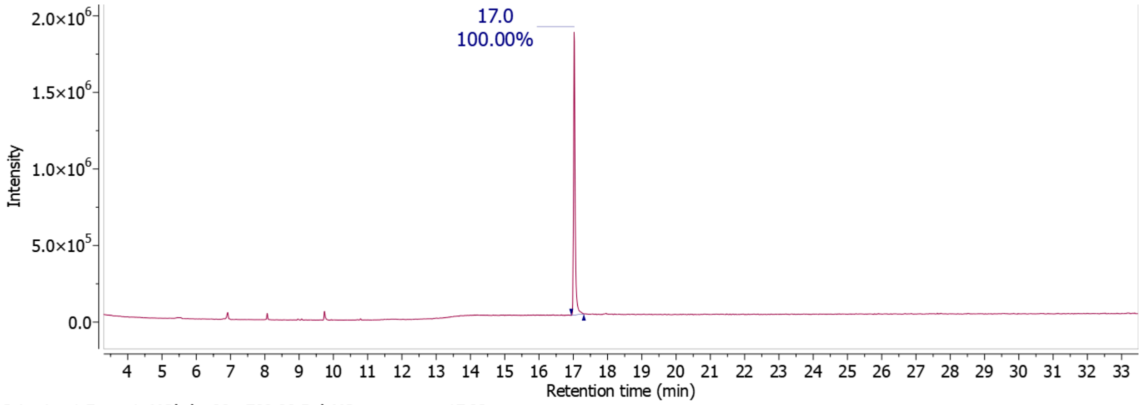

Injection 1 Event 1: MS(+)...00 - 700.00 Da) MS + spectrum 17.02

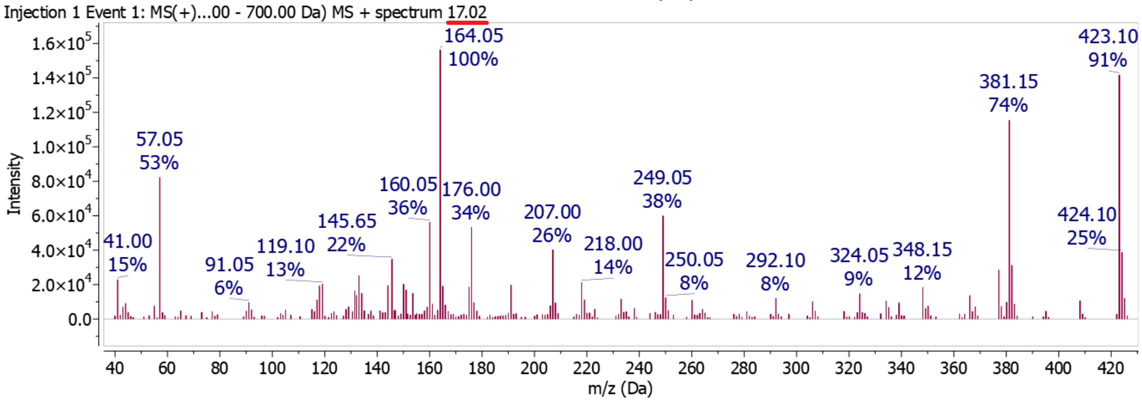

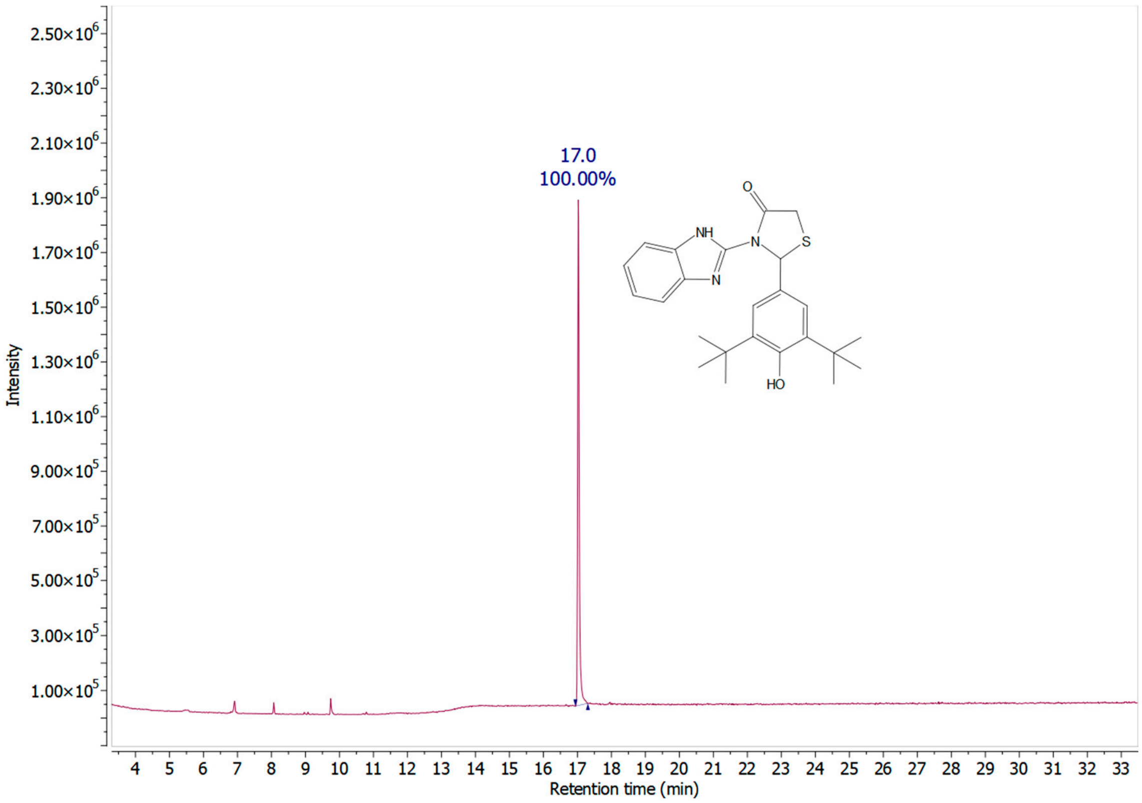

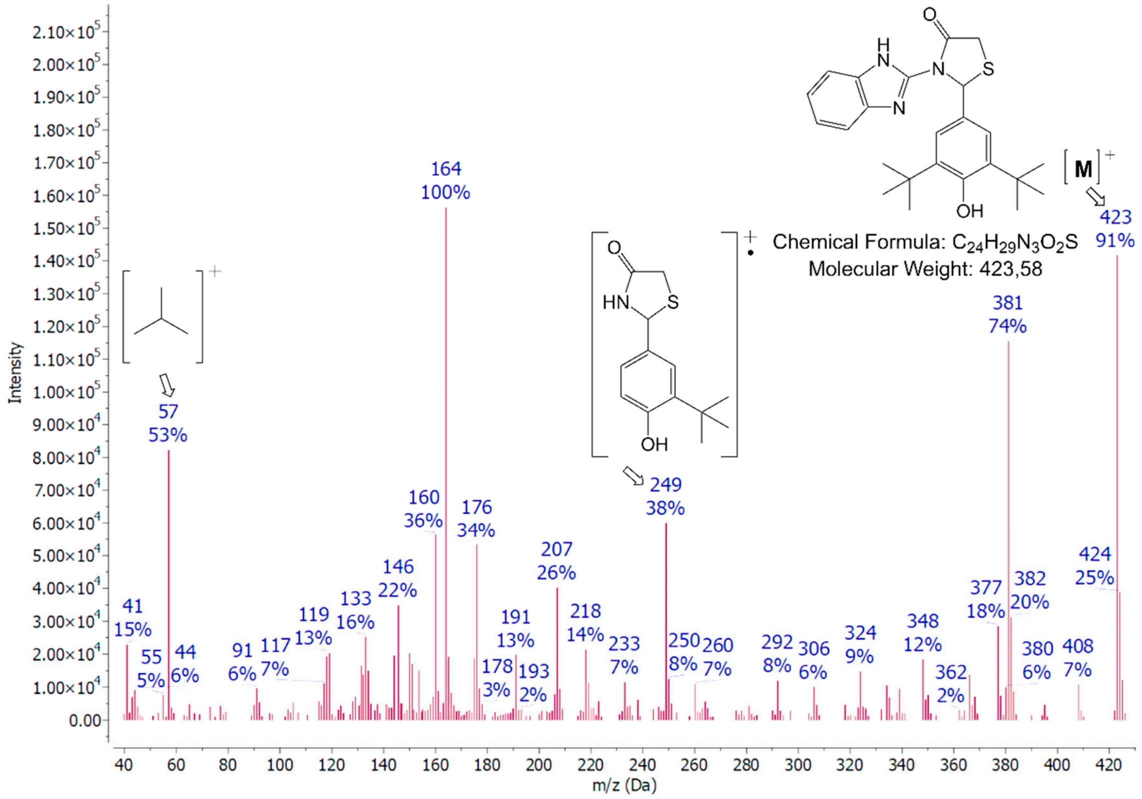

Figure S16. Chromatogram, mass spectra and fragmentation spectra of compound **4f**.

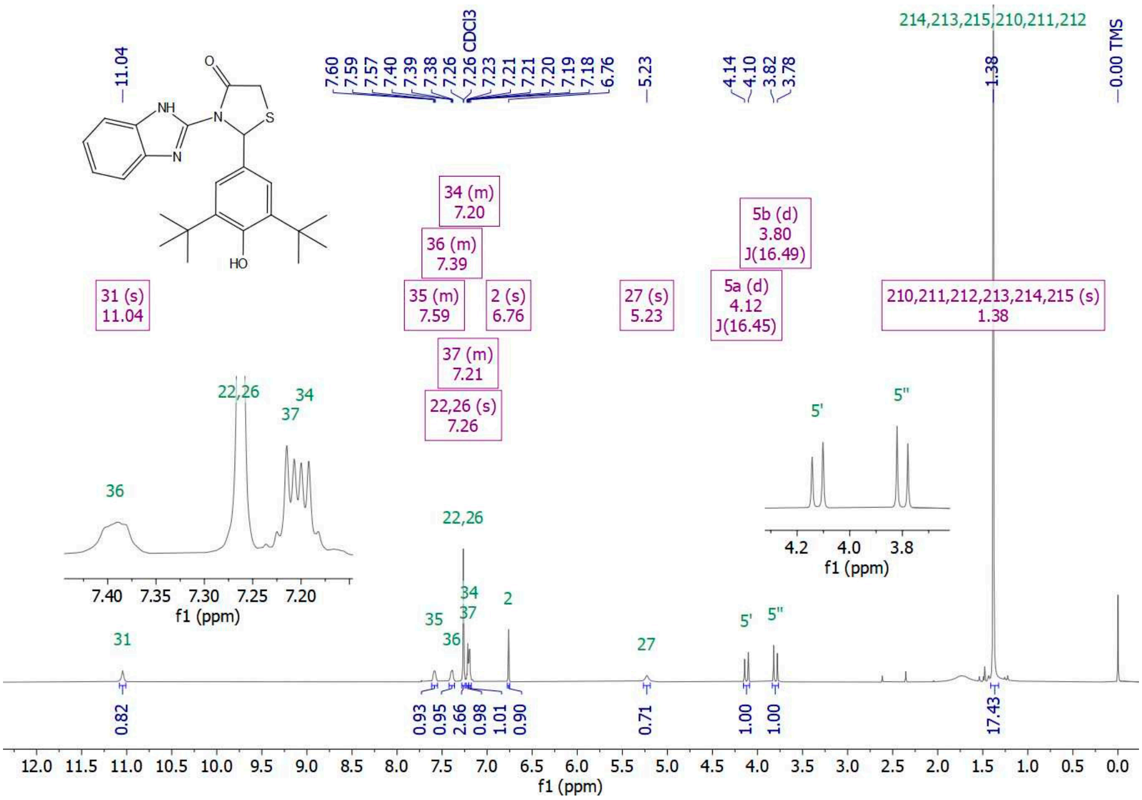

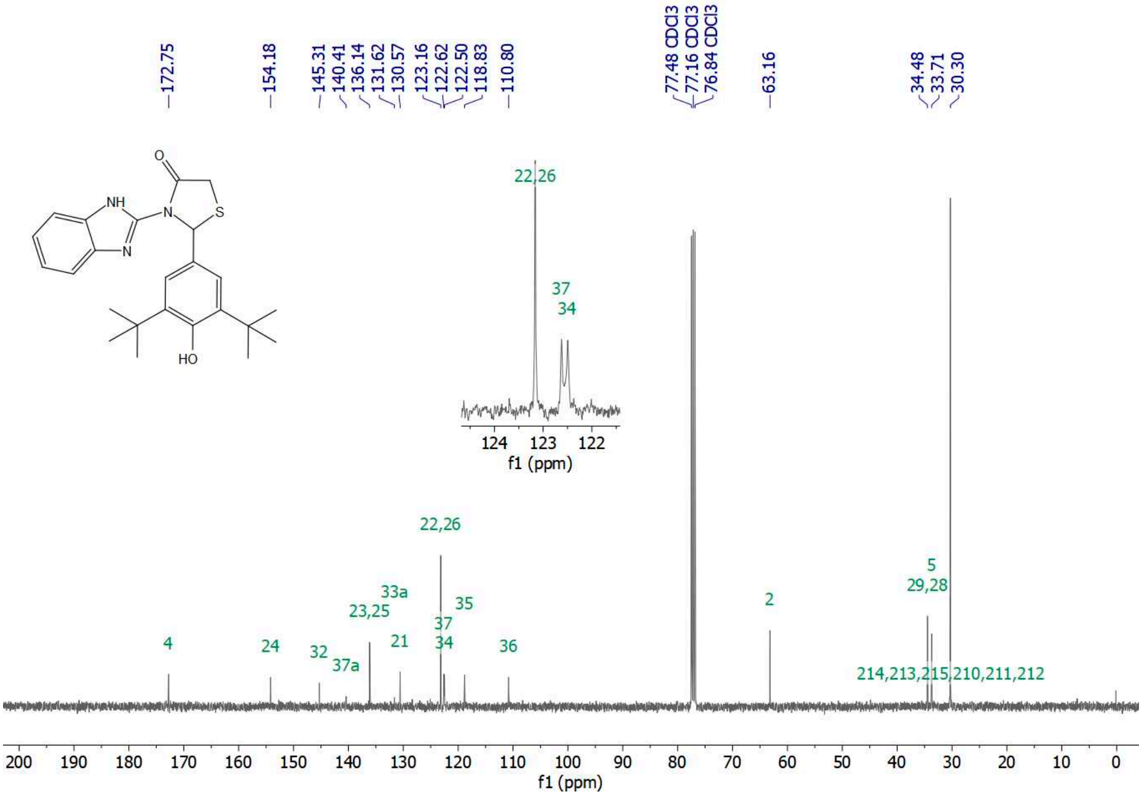

Figure S17. ^{1}H and ^{13}C NMR spectra of compound **4f**.

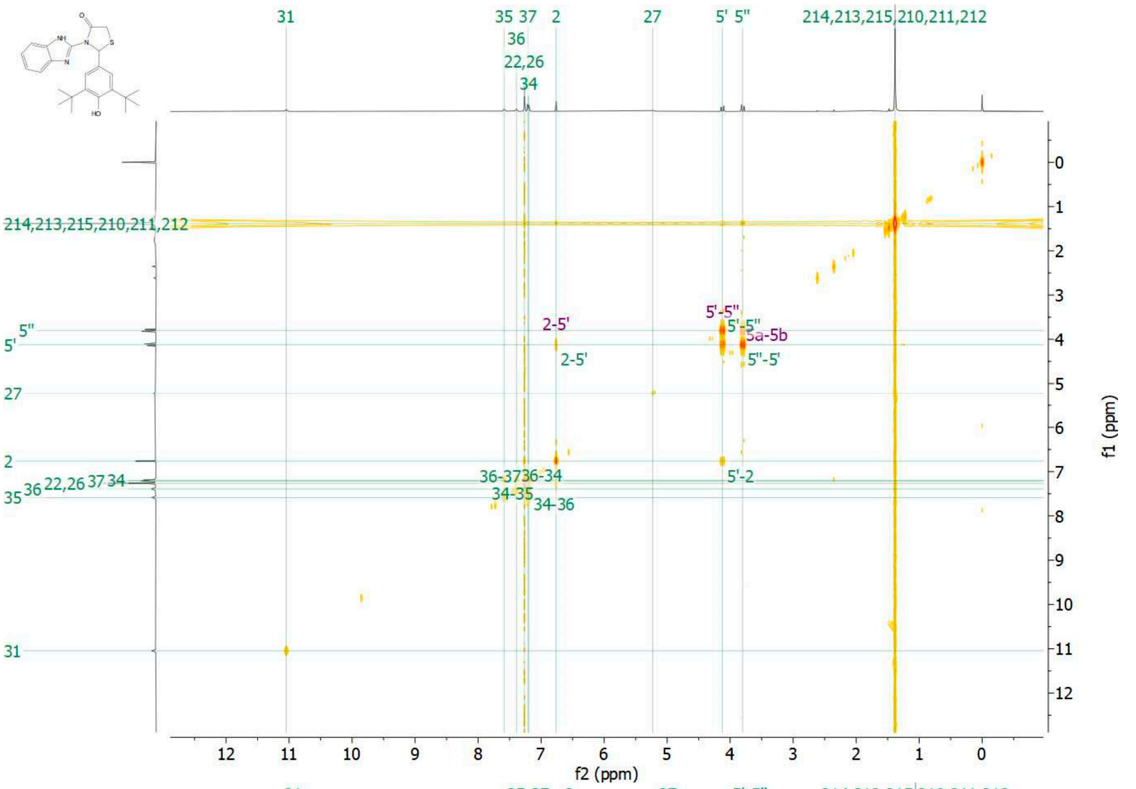

31
35 37 2
36
22,26
34
27
5' 5''
214,213,215,210,211,212
2-5'
5'-5''
5'-5''
5a-5b
5''-5'
2-5'
36-3736-34
34-35
34-36
5'-2
35 36 22,26 37 34
f2 (ppm)
f1 (ppm)

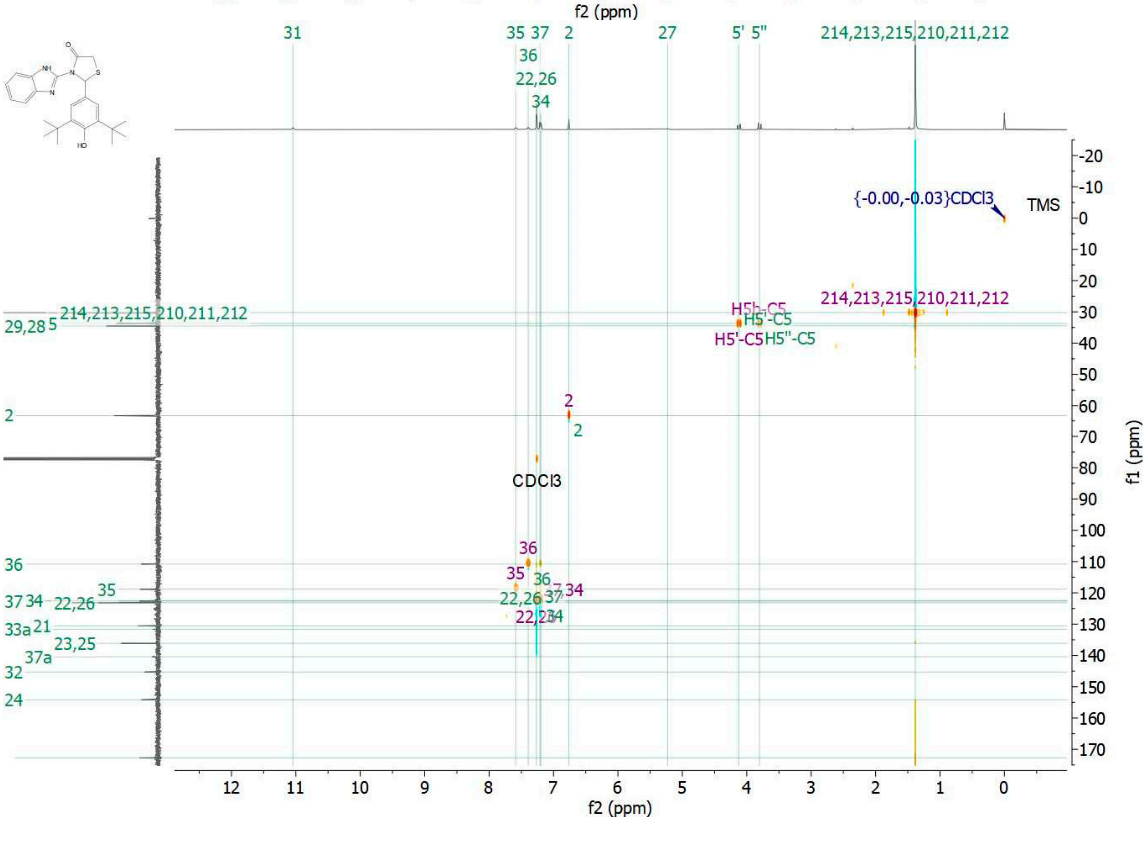

31
35 37 2
36
22,26
34
27
5' 5''
214,213,215,210,211,212
{-0.00,-0.03}CDCl3
TMS
H5b-C5
H5'-C5
H5'-C5 H5''-C5
29,28 5
CDCl3
36
35
22,26
37 34
33a 21
23,25
37a
32
24
f2 (ppm)
f1 (ppm)

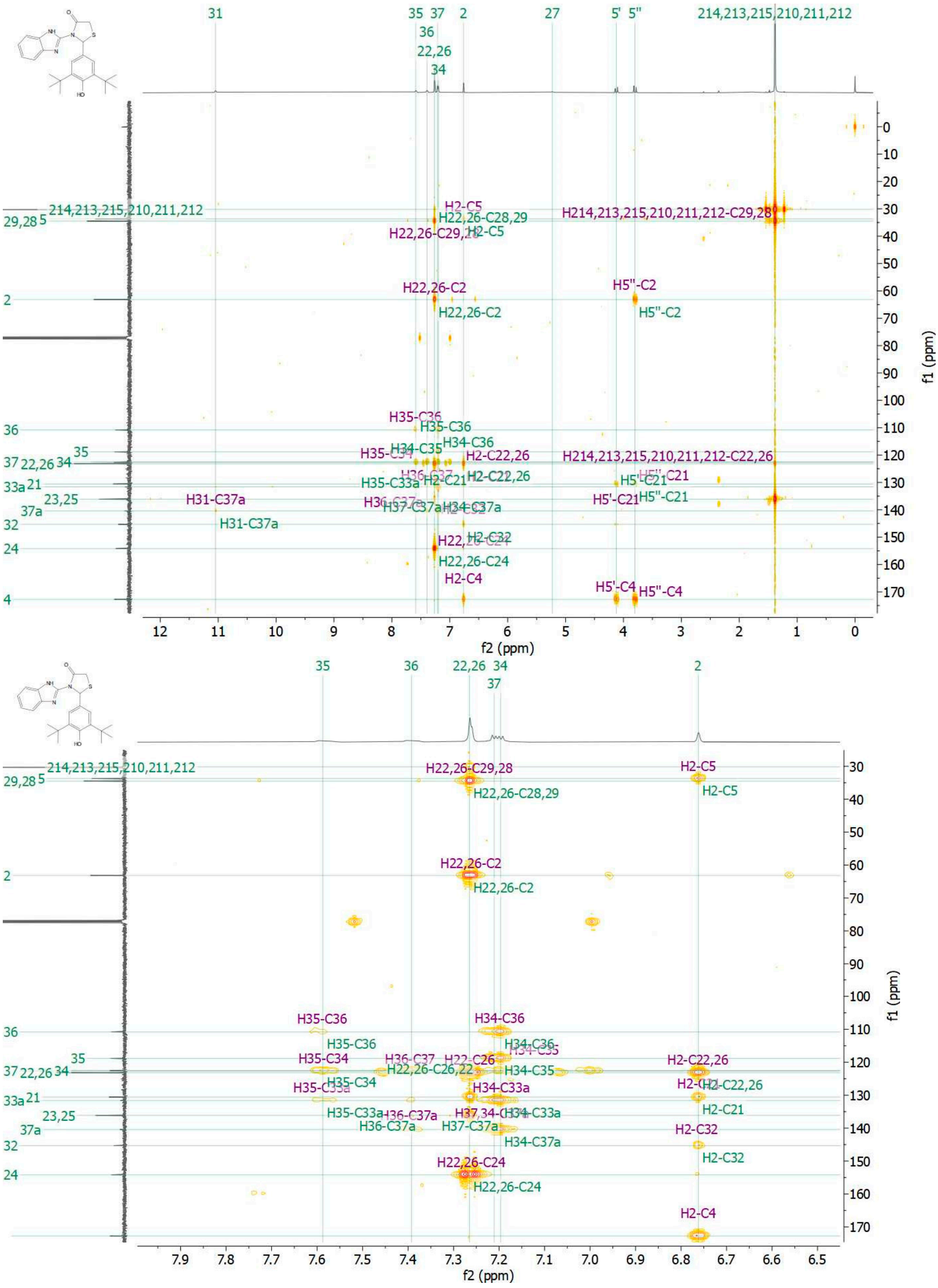

Figure S18. COSY, HSQC and HMBC NMR spectra of compound **4f**.

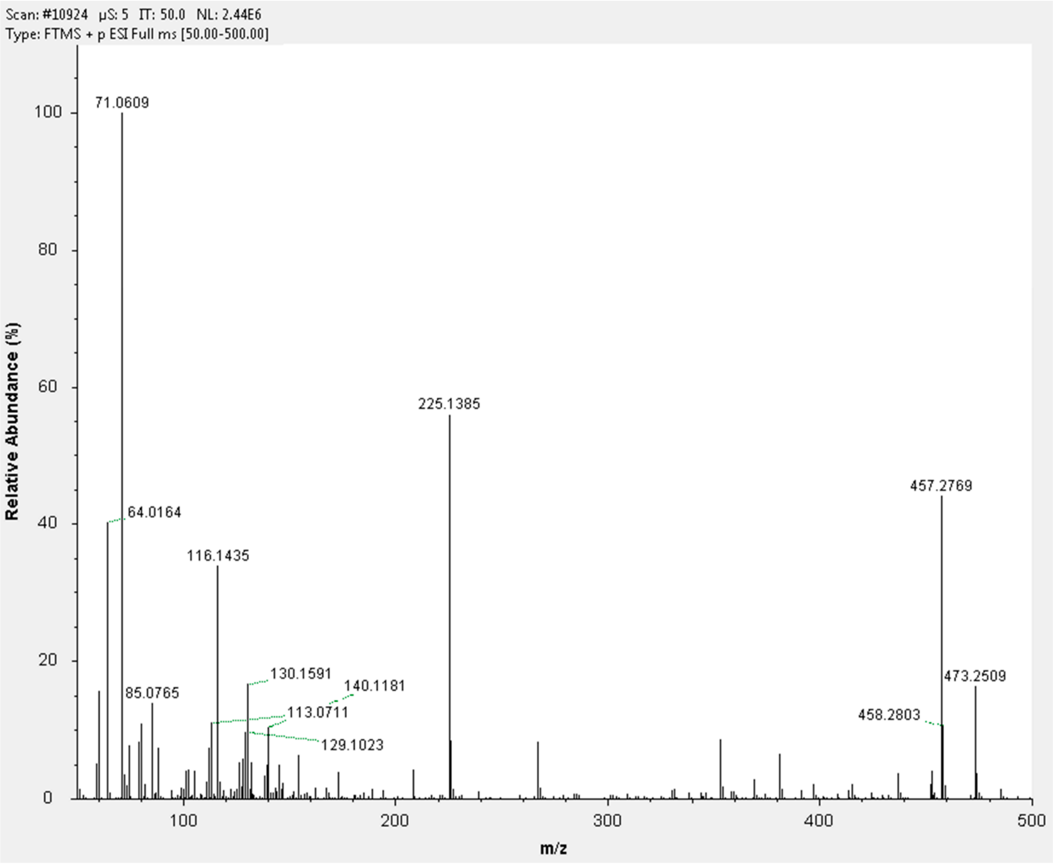

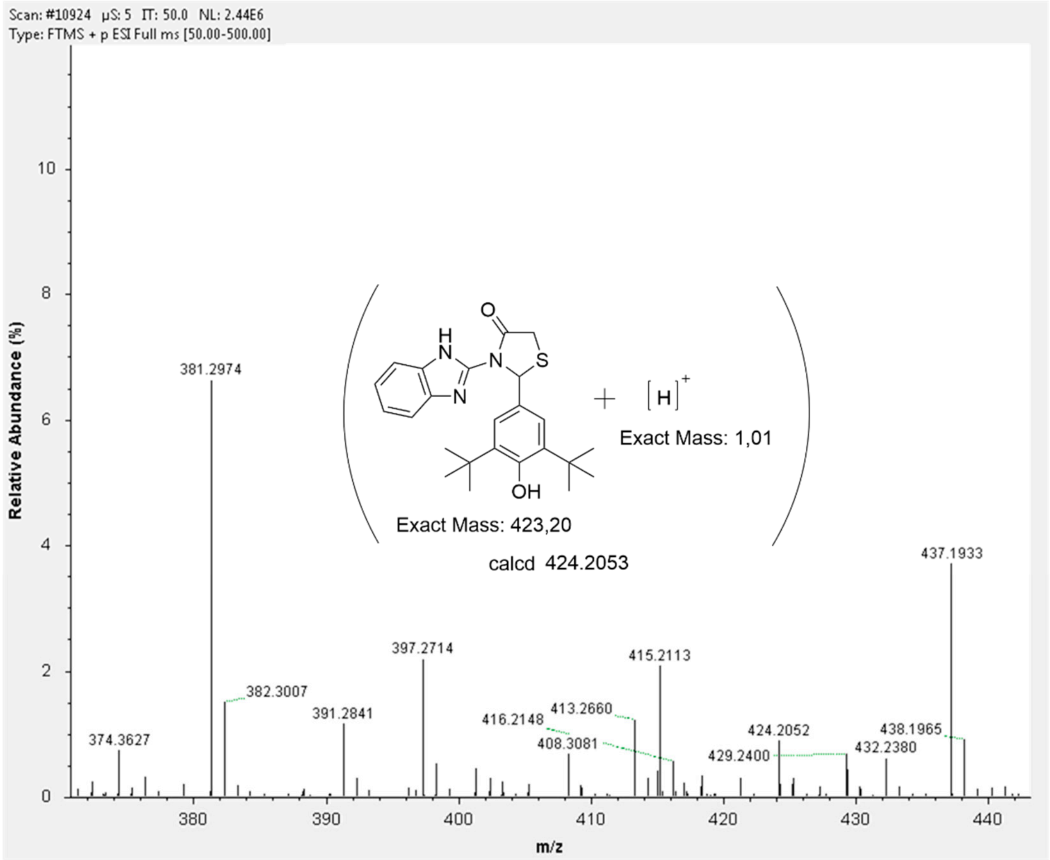

Figure S19. HRMS (ESI$^+$) of compound **4f** showing the $[M + H]^+$ ion and adducts from fragmentation.

1.7. 2-(3,5-di-*tert*-butyl-4-hydroxyphenyl)-3-(1,5-dimethyl-3-oxo-2-phenyl-2,3-dihydro-1H-pyrazol-4-yl)thiazolidin-4-one **4g**

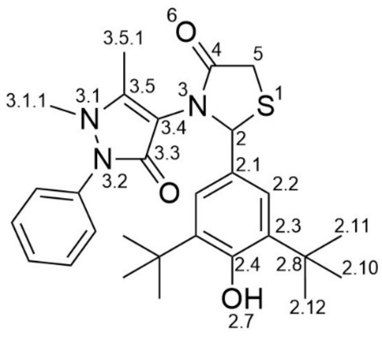

$C_{28}H_{35}N_3O_3S$, M.W.: 493.67 g/mol, CLogP: 4.03, light orange solid, yield: 0.4588 g (92%), m.p.: 223-225ºC.

TLC System: 7:3 Hexane:Ethyl Acetate. Rf: 0.0625

^{1}H NMR (400 MHz, $CDCl_3$): δ (ppm, J_{H-H} = Hz): 7.36 (t, ^3J = 7.80 Hz, 2H, H3.2.3, H3.2.5), 7.24 – 7.17 (m, 3H, H3.2.2, H3.2.4, H.3.2.6), 7.15 (s, 2H, H2.2, H2.6), 6.22 (s, 1H, H2), 5.21 (s, 1H, H2.7), 3.84 (d, ^2J = 15.71, 1H, H5b), 3.76 (dd, ^2J = 15.77, ^4J = 1.59 Hz, 1H, H5a), 2.87 (s, 3H, H3.1.1), 1.77 (s, 3H, H3.5.1), 1.30 (s, 18H, H2.10-215).

^{13}C NMR (100 MHz, $CDCl_3$) δ (ppm): 170.9 (C4), 161.4 (C3.3), 154.5 (C2.4), 153.6 (C3.5), 136.0 (2C, C2.3, C2.5), 134.7 (1C, aryl), 129.3 (2C, aryl), 128.9 (C2.1), 127.1 (1C, aryl), 125.4 (2C, aryl), 124.3 (2C, C2.2, C2.6), 107.7 (C3.4), 63.6 (C2), 35.9 (C3.1.1), 34.5 (2C, C2.8, C2.9), 33.4 (C5), 30.4 (6C, C2.10-C2.15), 11.1 (C3.5.1).

GC: T_R = 28,5 minutes.

MS (70 eV): m/z (%) = 494 (M^{+1}, 10), 493 (M^+, 29), 419 (11), 418 (23), 327 (33), 248 (10), 246 (10), 233 (37), 207 (23), 189 (18), 188 (56), 96 (14), 77(12), 57 (42), 56 (100).

HRMS (ESI) m/z: $[M + H]^+$ calculated exact mass (Trace Finder) for $C_{28}H_{35}N_3O_3S$ = 494.2471, found = 494.2470.

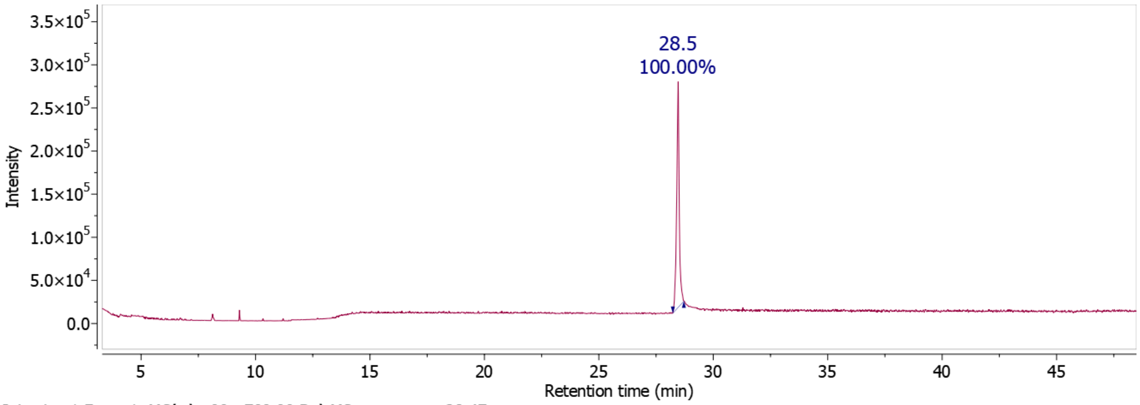

Injection 1 Event 1: MS(+)...00 - 700.00 Da) MS + spectrum 28.47

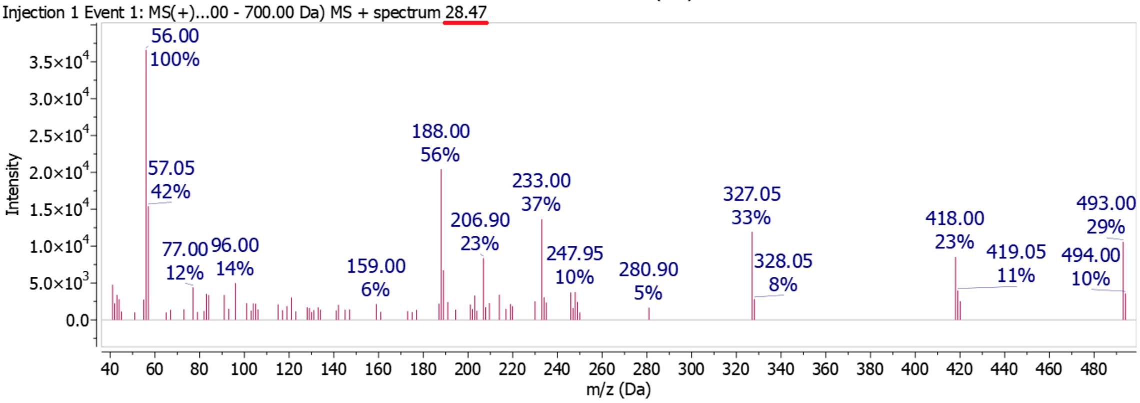

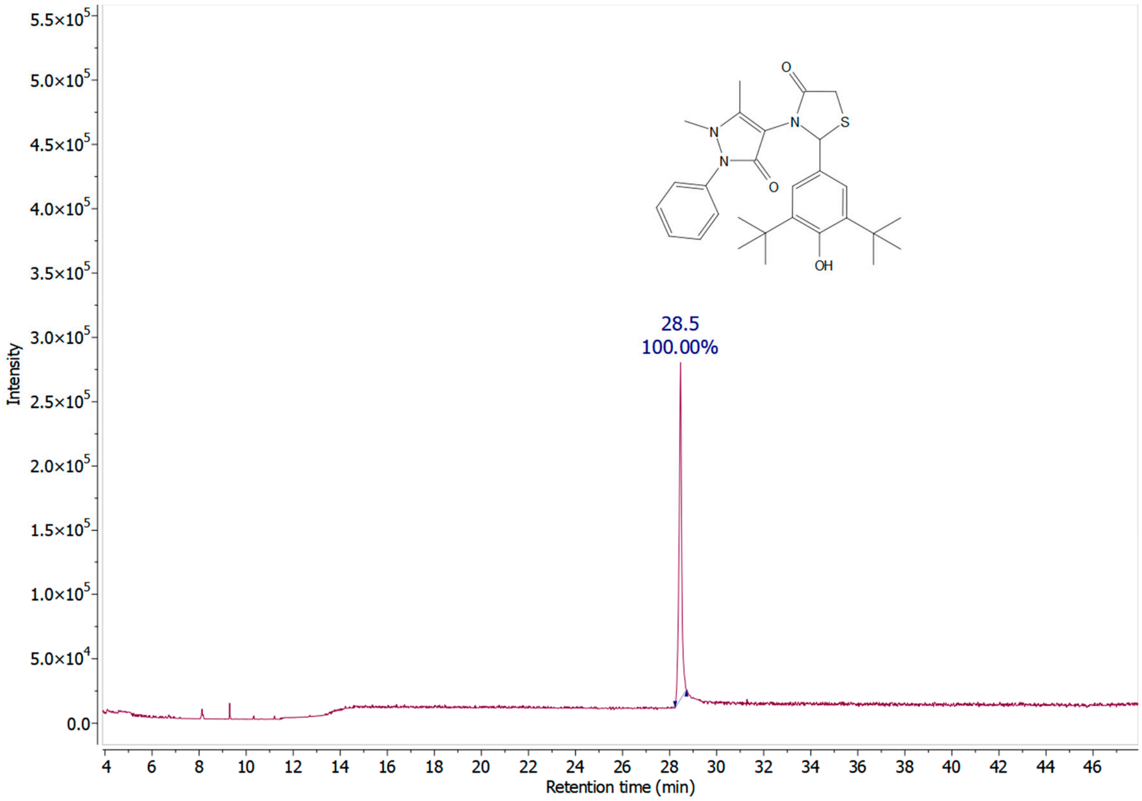

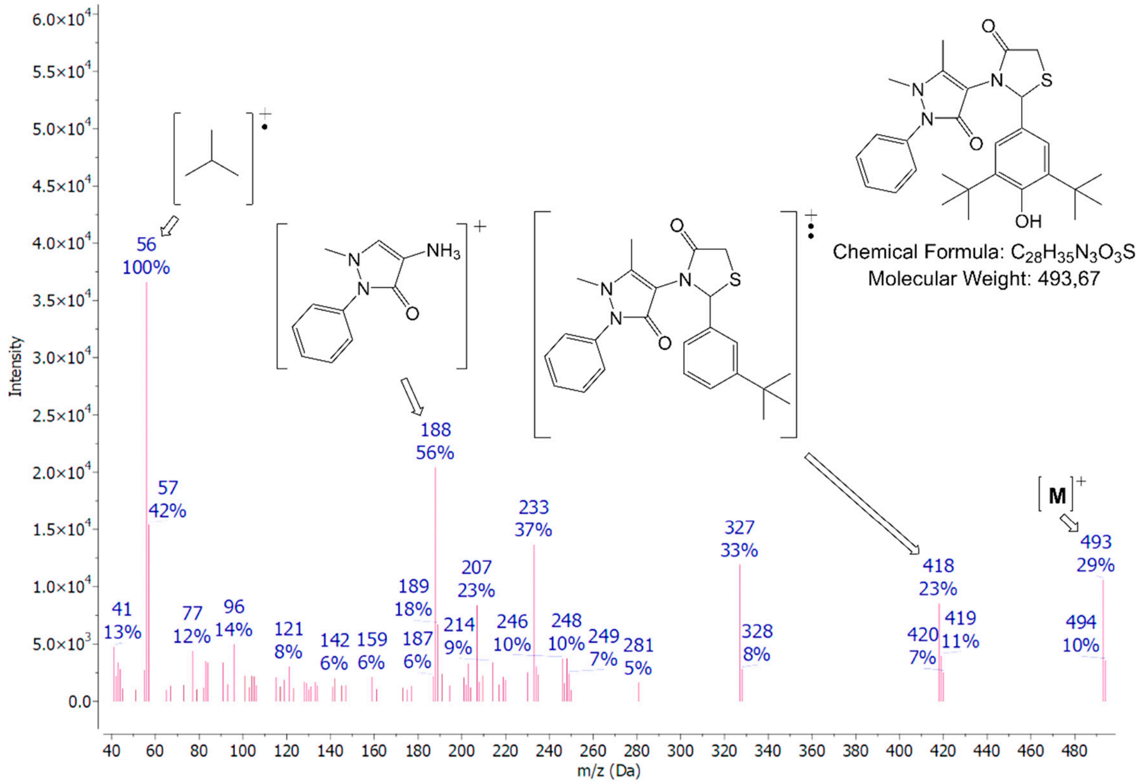

Figure S20. Chromatogram, mass spectra and fragmentation spectra of compound **4g**.

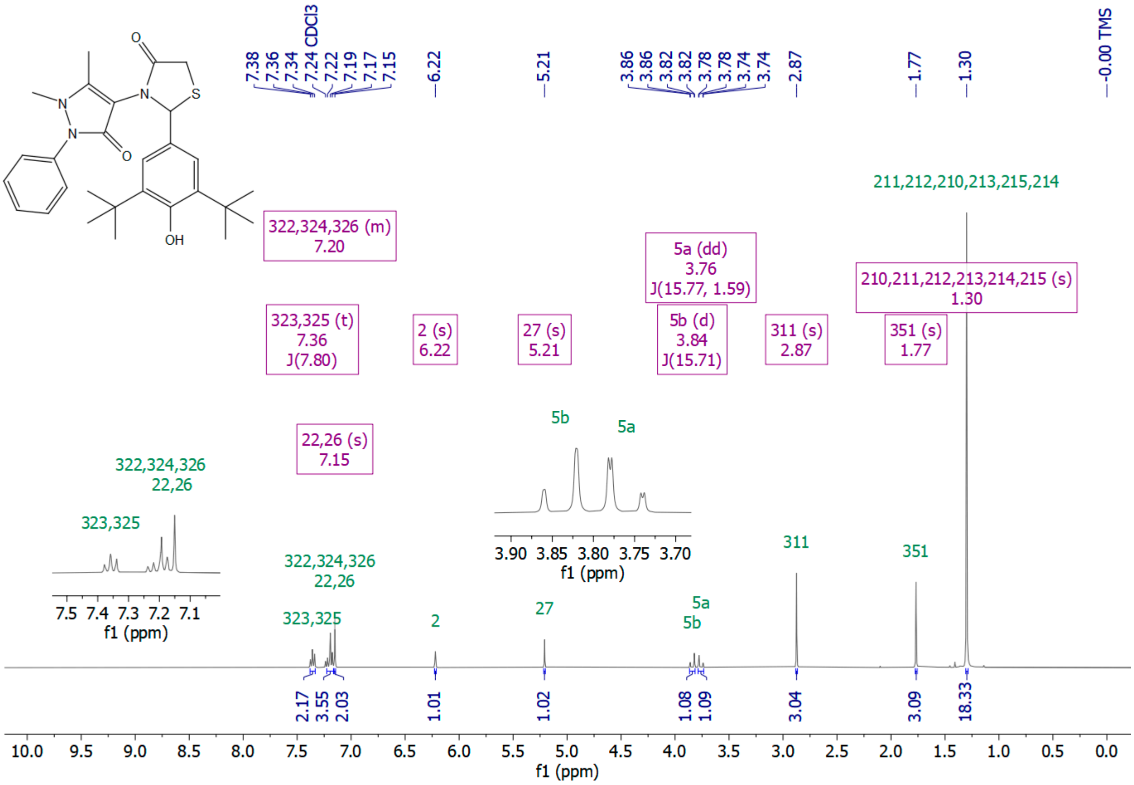

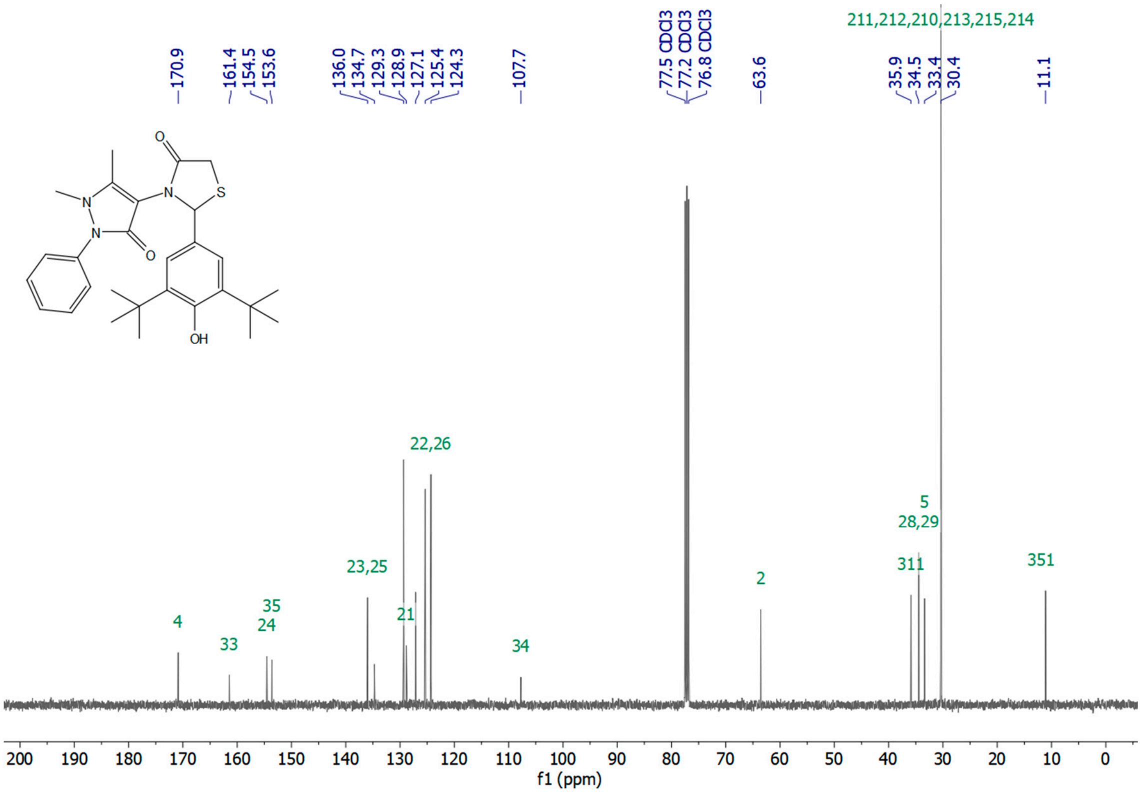

Figure S21. ^{1}H and ^{13}C NMR spectra of compound **4g**.

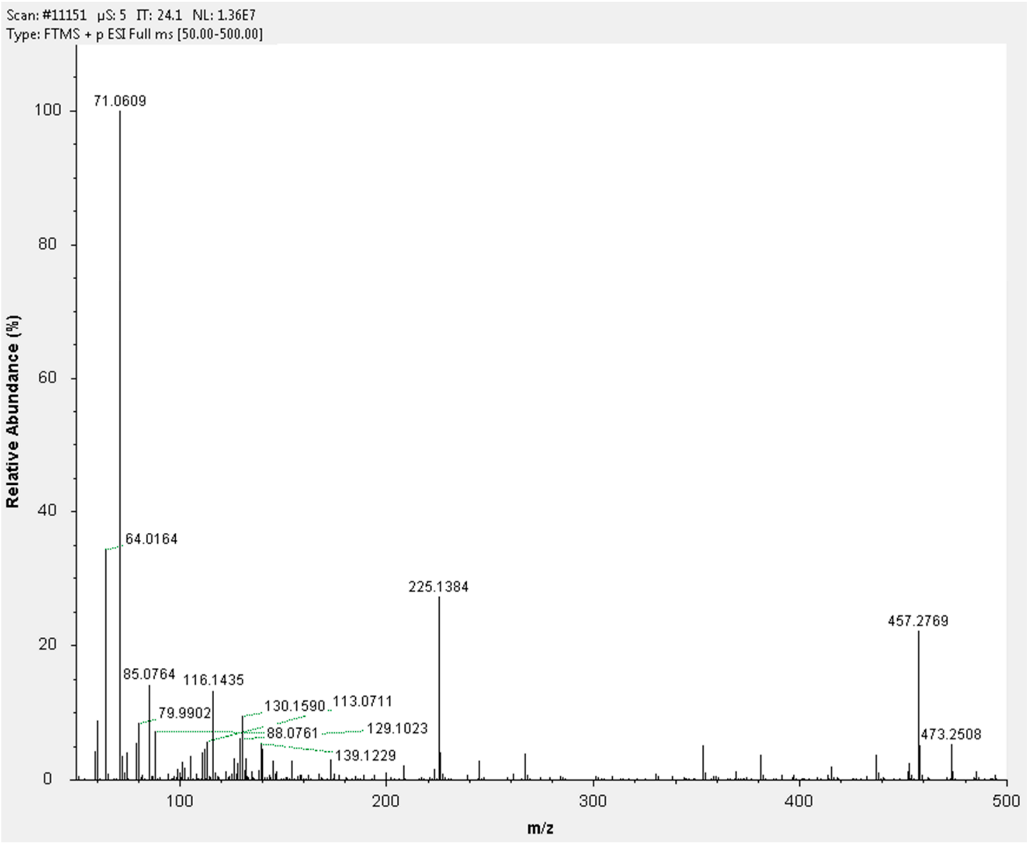

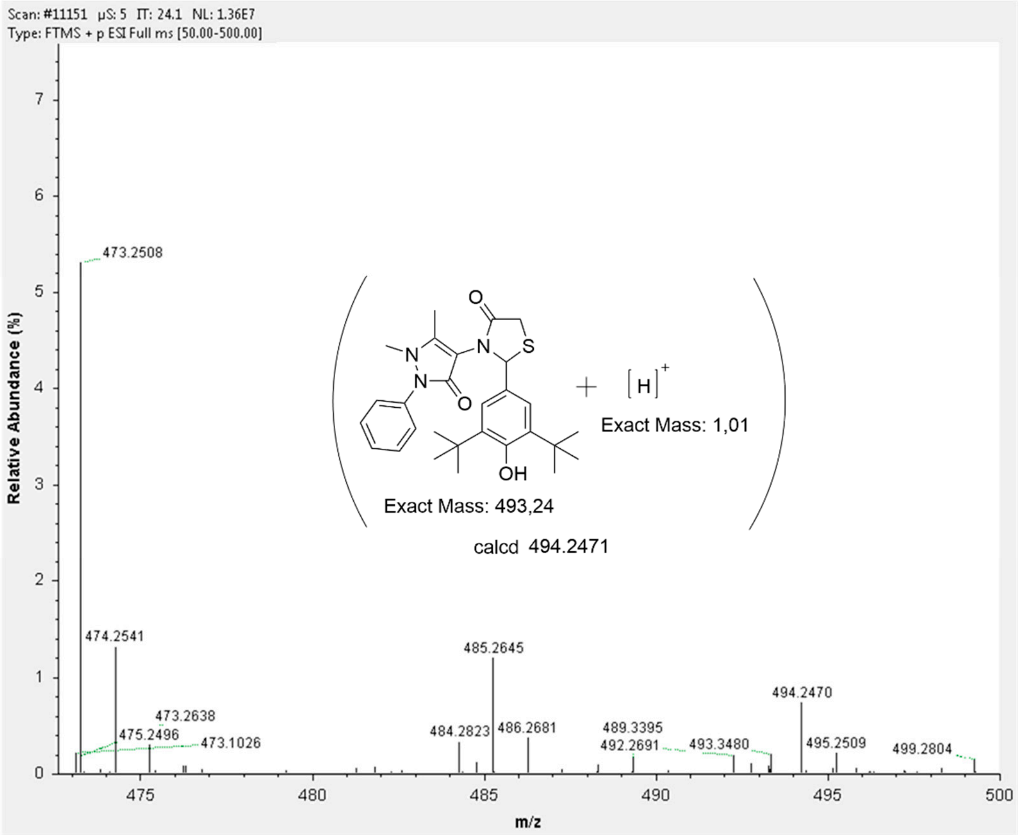

Figure S22. HRMS (ESI$^+$) of compound **4g** showing the $[M + H]^+$ ion and adducts from fragmentation.

Molecular docking

We selected several enzymes for molecular docking based on their documented biological relevance in trichomonads. Cysteine proteases, including cathepsin- and papain-like proteases, have been implicated in host protein degradation and parasite virulence in *Trichomonas* spp. (e.g., secretion and functional role of CPs such as TvCP4) [24]. Triosephosphate isomerase and lactate dehydrogenase are key glycolytic enzymes essential for anaerobic energy production in *Trichomonas*, with TPI being influenced by glucose levels and surface-associated functions described in the parasite [25] and LDH contributing to NAD^+ regeneration required for continuous glycolysis [26]. Methionine γ-lyase, absent in mammalian hosts, participates in sulfur amino acid metabolism and represents a distinctive metabolic enzyme in *T. vaginalis* [27]. Purine nucleoside phosphorylase is part of the essential purine salvage pathway, as *Trichomonas* cannot synthesize purines de novo [28]. Finally, thioredoxin reductase plays a central role in the parasite's redox defense and has been validated as an antitrichomonal target with inhibitors like auranofin showing efficacy *in vitro* [29]. These selections were thus grounded in well-characterized parasite biology and represent plausible targets for hypothesis-driven docking, while acknowledging that experimental validation remains necessary.

Receptor and ligand preparation utilized MGLTools with AutoDockTools [30]. Ligand file format conversion was performed with Open Babel. Molecular docking simulations were executed using AutoDock Vina [31]. Post-docking interaction analysis was conducted with the PLIP [32] and 2D interaction diagrams were generated using PoseView [33]. Data aggregation, parsing of interaction reports, and calculation of ligand efficiency were carried out using custom Python (v3.9) scripts employing the pandas and ElementTree libraries.
